# Integrated GIS-machine learning approach to irrigation water quality assessment in coastal aquifers

**DOI:** 10.1038/s41598-025-25461-y

**Published:** 2026-01-28

**Authors:** Loubna Nefla, Amira Bergal, Warda Boumaraf, Samira Gheid, Chahrazed Bouksiba, Hichem Khammar, Fulvio Celico, Hichem Nasri, Aissam Gaagai, Salah Elsayed, Mohamed S. Abd El-baki, Abdullah M. Attiah, András Székács, Omar Saeed, Mohamed Gad

**Affiliations:** 1 Laboratory of Biodiversity and Ecosystem Pollution, Faculty of Life and Nature Sciences, Chadli Bendjedid University, El-Tarf, BP 73, 36000 El Tarf, Algeria; 2 Laboratory of Environmental Sciences and Agroecology, Faculty of Natural and Life Sciences, Department of Biology, University Chadli Bendjedid El Tarf, BP 73, 36000 El Tarf, Algeria; 3 Laboratory of Molecular and Cellular Biology, Faculty of Natural and Life Sciences, Earth and the Universe, University of Guelma, Algeria, BP 401, 24000 Guelma, Algeria; 4 Laboratory of Functional Ecology and Environment, Department of Life and Nature Sciences, Faculty of Exact Sciences and Life and Nature Sciences, University of “Larbi Ben M’hidi”, 04000 Oum El Bouaghi, Algeria; 5 Department of Chemistry, Life Sciences, and Environmental Sustainability, University of Parma, Parco Area delleScienze 157/A, 43124 Parma, Italy; 6 Scientific and Technical Research Center on Arid Regions (CRSTRA), 07000 Biskra, Algeria; 7 Agricultural Engineering, Evaluation of Natural Resources Department, Environmental Studies and Research Institute, University of Sadat City, 32897 Minufiya, Egypt; 8 Agricultural Engineering Department, Faculty of Agriculture, Mansoura University, 35516 Mansoura, Egypt; 9 Central Laboratory for Elemental and Isotopic Analysis (CLEIA), Nuclear Research Center (NRC), EgyptianAtomic Energy Authority (EAEA), Inshas, Egypt; 10 Agro-Environmental Research Centre, Institute of Environmental Sciences, Hungarian University of Agriculture and Life Sciences, Páter Károly u. 1, 2100 Gödöllő, Hungary; 11 Doctoral School of Environmental Science, Hungarian University of Agriculture and Life Sciences (MATE), Páter Károly u. 1, 2100 Gödöllő, Hungary; 12 Hydrogeology, Evaluation of Natural Resources Department, Environmental Studies and Research Institute, University of Sadat City, 32897 Minufiya, Egypt

**Keywords:** Irrigation groundwater quality, Hydrogeochemical properties, Machine learning algorithms, GIS techniques, Algeria, Environmental sciences, Hydrology

## Abstract

Groundwater has become a vital and increasingly relied-upon resource, especially in semi-arid and arid regions. Thus, to ensure groundwater complies with standards before use, continuous monitoring and comprehensive quality assessment are essential. This study was conducted to assess the quality of groundwater (GW) in the Skikda aquifer, northeastern Algeria, for irrigation using irrigation water quality indices (IWQIs), multivariate statistical analysis, and machine learning algorithms (MLAs): Random Forest regression (RF), Extreme Gradient Boosting regression (XGBR), and Adaptive Boosting Regression (ABR), integrated with SHAP analysis. Forty-four groundwater samples were collected from the study area during summer and winter seasons and analysed for temperature, pH (6.25–9.29; mean 7.24), electrical conductivity (EC: 532–5830 µS/cm; mean 1798.22 µS/cm), turbidity, total dissolved solids (TDS: 258–3020 mg/L; mean 962.33 mg/L), and concentrations of calcium (Ca^2+^: 40–366 mg/L), magnesium (Mg^2+^: 10–167 mg/L), sodium (Na^+^: 62–510 mg/L), potassium (K^+^: 0.40–45.6 mg/L), chloride (Cl^–^: 30.9–1800 mg/L), bicarbonate (HCO_3_^–^: 198–695 mg/L), sulfate (SO_4_^2–^: 1–429 mg/L), and nitrate (NO_3_^–^: 0.12–2.99 mg/L).The dominating hydrochemical facies in the study area were Mg-Ca-SO_4_, accompanied by the Sodium-Chloride (Na-Cl).Principle Component Analysis (PCA) for summer and winter datasets identified four key components suggesting a strong correlation between variables and factors, with PCA indicating that geochemical processes, such as rock0water interaction and dissolution of evaporate minerals, control the groundwater’s chemical composition. Groundwater quality for irrigation varied across the samples, with most exhibiting moderate to high constraints based on IWQI (26.88–74.00; mean 54.56 in summer and 56.70 in winter). Sodium Adsorption Ratio (SAR: 1.13–7.10) and Permeability Index (PI: 33.06–83.39) suggested excellent to good water quality, while Sodium Percent (Na%: 19–69%),) and Soluble Sodium Percentage (SSP: 19–70%) indicate a small but significant fraction (9–16%) of in appropriate samples. Magnesium Hazard (MH: 22–62) and SSP indicated that most samples were safe. Compared to winter, summer samples showed slightly poorer quality (higher Na%, SSP, and lower IWQI), likely due to evaporative solute concentration. Random Forest (RF) model integrated with SHAP analysis showed superior predictive accuracy for all Water Quality Indices (WQIs), with strong validation results (R^2^ > 0.95; RMSE < 0.5) for both seasons. A comparative evaluation of RF, XGBR, and ABR further highlighted differences in predictive performance, with RF consistently providing the most reliable predictions of WQIs across both seasons (R^2^ = 0.73–0.94). These results highlight RF’s effectiveness in predicting WQIs and highlight the influence of seasonal geochemical processes on groundwater quality, requiring the development of management strategies for sustainable irrigation. This study presents a novel approach by combining machine learning algorithms with GIS-supported hydrogeochemical assessment to evaluate irrigation water quality in a semi-arid coastal aquifer of Algeria. By integrating advanced ML techniques (RF, XGBR, ABR) with water quality indices and SHAP analysis, it captures the complex interactions between natural geochemical processes and human activities. The approach offers a replicable framework for sustainable groundwater management in underexplored regions.

**Supplementary Information:**

The online version contains supplementary material available at 10.1038/s41598-025-25461-y.

## Introduction

 Water is considered a fundamental resource for life and health, faces increasing threats globally, necessitating both protection and quality improvement^1^. Representing about 99% of Earth’s liquid freshwater, groundwater is an essential global water resource and a key component of worldwide reserves. Groundwater is a vital component of the hydrological cycle^2^, which particularly vulnerable to pollution from urban expansion, agriculture, and industrial development^3^. Rapid socioeconomic growth has exacerbated environmental issues, including groundwater depletion and land subsidence in various regions^4,5^, that making the safeguarding water quality a global priority^6^.

Groundwater is especially crucial in arid and semi-arid regions, where limited availability and intensive use pose serious challenges^7,^^8^. In Algeria, groundwater represents nearly 65–70% of total freshwater withdrawals, with agriculture consuming more than 70% of this resource. In coastal low-lying areas, salinization threatens agricultural output, demanding urgent management strategies^9,10^. Salinization challenges in groundwater of low-lying coastal areas pose significant concerns, impacting agricultural production and necessitating a comprehensive review of management strategies^11^. These unsustainable practices have already contributed to falling water tables and deteriorating groundwater quality^12^.

In regions with scarce surface water, groundwater serves as the primary drinking water source and sustaining agriculture^13,14^. Algeria’s socioeconomic progress is significantly reliant on groundwater reserves, which face threats from both natural and human-induced hazards^15,16^. To better understand such dynamics, many global studies have applied water quality indices (WQIs)^17,18^.

Given this context, groundwater monitoring becomes critical for meeting growing water needs in terms of both availability and quality. The WQIs are essential tools that consolidate multiple physical and chemical parameters into a single numerical value, enabling an efficient assessment of water quality^19,20^. Extensive research has evaluated groundwater hydrochemistry and quality using WQIs, GIS, multivariate statistics, and machine learning (ML)^21,22^.

The Skikda region, in northeastern Algeria illustrates the combined challenges of water scarcity, agricultural expansion, and industrial development. Agriculturally, the cultivated area extends over approximately 193,179 ha, including 15,300 ha of irrigated land, with diversified fruit and vegetable production. Strawberries alone occupy 52 ha, yielding around 4,100 quintals annually^23^. The region relies heavily on groundwater for irrigation and domestic supply, as population growth and urbanization intensify demand. On the industrial side, Skikda hosts Algeria’s largest oil refinery (16.5 million tons/year), a condensate refinery, a major petrochemical complex, and a commercial port handling over 22 million tons of goods annually^24^. This overlap between intensive agriculture and large-scale industry significantly increases the vulnerability of groundwater resources.

Several studies have been conducted on groundwater in the Skikda region, focusing on its quality and hydrogeological characteristics. An evaluative study in the industrial zones of Skikda revealed significant groundwater contamination with heavy metals such as chromium, lead, and arsenic, with concentrations exceeding the thresholds set by the World Health Organization (WHO), highlighting the strong influence of industrial activities on groundwater quality^25^. Similarly, research on pollution caused by the abandoned Sidi Kambar mine indicated severe deterioration in both groundwater and surface water quality, including reduced suitability for drinking and potential health risks for local populations^26^. Hydrogeological assessments including the DRASTIC model were identified medium-to-high vulnerability zones near Skikda City^27^. Similarly, hydrochemical studies in the Fetzara basin found that 88% of samples were suitable for irrigation, and about 64% had elevated magnesium that could impair soil fertility^28^. However, these works largely relied on conventional hydrochemistry and classical modeling, without integrating advanced methods, such as machine learning (ML) capable of capturing complex hydrochemical–environmental interactions.

A novel approach using multivariate statistical techniques specifically cluster analysis (CA) and principal component analysis (PCA) has been implemented. This quantitative method categorizes groundwater samples to investigate physicochemical parameter interrelationships, assess sample similarities, and determine groundwater mineralization processes^29,30^. Groundwater quality is significantly influenced by physicochemical patterns arising from geological settings and human practices^31^. Indeed, recharge, aquifer properties, contact time, and geochemical processes like mineral dissolution, and ion exchange are main factors in controlling the groundwater geochemistry for irrigation^32,33^. Classical tools including, Piper, Gibbs, Chadha diagrams, and ionic ratios are used to assess chemical characteristics and controlling factors^34^. To evaluate irrigation suitability, the IWQIs based on parameters like Na%, SSP, SAR, and MH are commonly used^35,36^. Geographic Information Systems (GIS) provide a promising approach by integrating water quality assessment methods with spatial analysis, thereby enhancing the interpretation and presentation of research findings^37^.

Machine learning algorithms (MLAs) have become powerful tools for groundwater quality prediction. Approaches, such as Random Forest Regression (RF), Extreme Gradient Boosting Regression (XGBR), and Adaptive Boosting Regression (ABR) demonstrate exceptional accuracy in capturing complex, nonlinear relationships within water quality datasets^22,38–47^. These models offer significant advantages over traditional methods, including faster processing, cost-effectiveness, and superior handling of large-scale data^44,48^. More recently, MLAs have supported robust forecasting frameworks that strengthen decision-making in water resource management. In addition, this study conducts a comparative analysis of the selected machine learning algorithms (RF, XGBR, ABR) to evaluate their predictive performance for irrigation water quality indices. Stating this comparative approach upfront ensures consistency with the discussion and conclusion sections and guides the reader on what to expect from the study.

Despite these advances, semi-arid coastal aquifers in northeastern Algeria remain underexplored, particularly regarding the application of MLAs for predicting diverse WQIs and assessing the combined impacts of salinity and heavy metals. Addressing these gaps is crucial for sustainable water quality management in this sensitive and economically important region. This study investigates the hydrodynamic behavior and key geochemical controls on groundwater in the semi-arid Skikda region. Specifically, it assesses the influence of natural and anthropogenic factors (agriculture, urbanization) on quality of the groundwater for agricultural suitability, and the study is preliminary in establishing the use of Random Forest regression (RF), Extreme Gradient Boosting regression (XGBR), Adaptive Boosting Regression (ABR) integrated with SHAP analysis for assessing Algerian irrigation groundwater resources in the northeast coastal region.

The originality of this study stems from the integration of water quality indices (WQIs) with GIS-based machine learning techniques to assess irrigation suitability in the Skikda region, where such a combined approach has not yet been implemented. The main objectives of this study are therefore to (i) evaluate the influence of natural processes and anthropogenic pressures on groundwater quality in Skikda, (ii) assess irrigation water quality using IWQIs, and (iii) develop and test machine learning models (RF, XGBR, ABR) integrated with GIS and SHAP to predict and interpret irrigation suitability. The results of this study can guide local water management authorities in adopting sustainable irrigation practices, while reducing potential risks to human health and the surrounding ecosystems.

## Materials and methods

### Study area

**Fig. 1:**
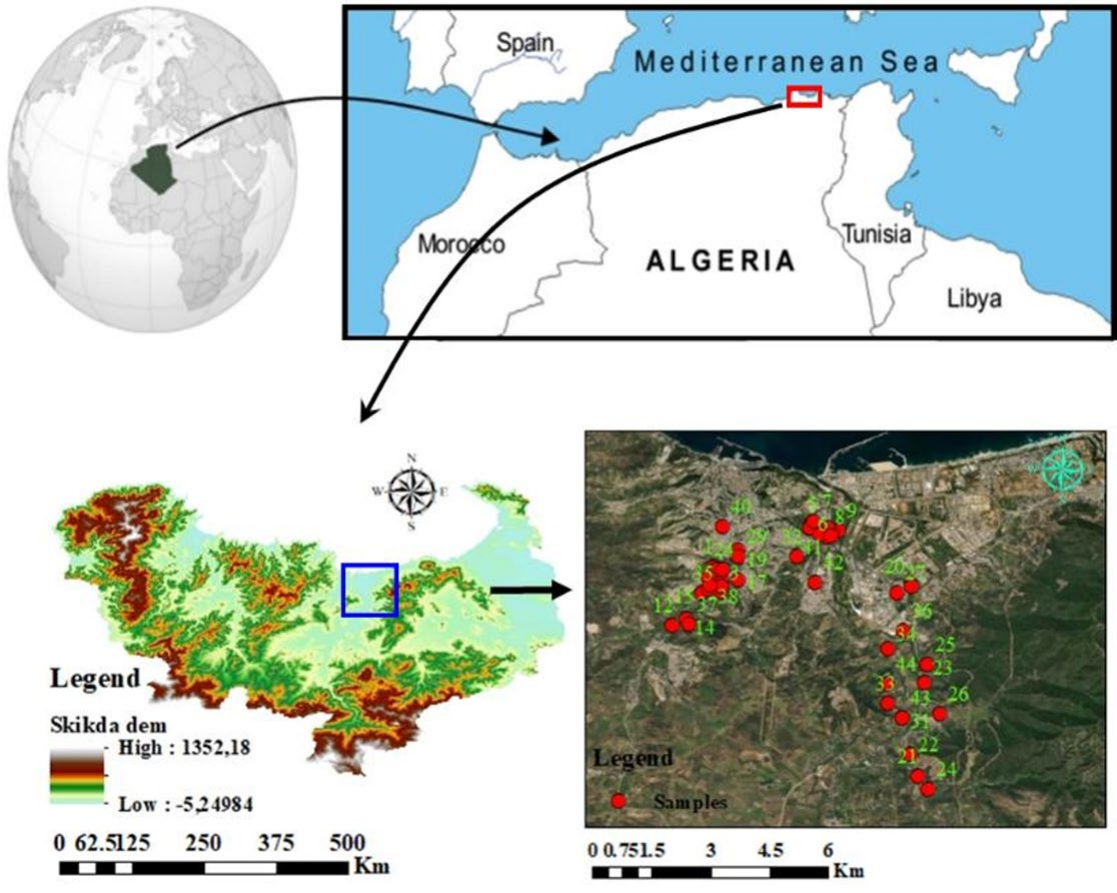
The Location map of the study area explains the geographical location, elevation and groundwater points. Map created using ArcGIS Pro 2.8.8 Software (Esri; https://www.esri.com/arcgis/about-arcgis).

The Skikda region, situated in northeastern Algeria and part of the small Kabylie region, is bordered to the north by the Mediterranean Sea and shares boundaries with the provinces of Annaba and Guelma (east), Constantine and Mila (south), and Jijel (west). The Skikda region covering an area of 55.82 km^2^ with a population of approximately 890,000^49^, which has a population density of 192 people per km^2^. The climate of the Skikda region is temperate sub-humid to humid, influenced by the Mediterranean Sea, and characterized by two distinct seasons: a cooler and wetter period from October to April, with a mean temperature of about 14 °C, and a warmer and drier period from May to September, with a mean temperature of 22 °C. The overall annual average temperature is around 18 °C, with an annual precipitation of 746.9 mm. The diverse topography of Skikda encompasses both rugged mountains and level plains, where altitudes vary between 300 m and 1000 m above sea level, which significant influencing groundwater distribution and accessibility. Groundwater in mountainous areas is typically found at greater depths, complicating extraction, while plain areas have shallower levels, making access easier for irrigation and drinking. The varied topography of Skikda plays a significant role in the distribution and accessibility of groundwater across the region. The study area includes several representative sectors of Skikda, spanning its central, northwestern, and eastern parts. The central sector covers urban zones such as Cité Frère Ayachi P40, Cité Zéramna P32, and Hamadi Kerouma P42. The northwestern sector includes areas with substantial agricultural, industrial, and residential activity, such as industrial zones P5, P6, P7, P8, P9, P10, P11, P39, P41. The southwest sector comprises localities including Hadaik P1, P2, P4, P12, P13, P14, P15, P16, P30, P38, P37, P42, and Zefzef P3, P17, P18, P19, P35, P29. The southeast areas such as Beni Bechir P23, P25, P26, and west areas like Bissi P21, P22, P24, P28, and Bougaren P31, P33, P34, P44, and Cité Hamrouch Hamoudi P20, P43, P27 (Fig. 1).This geographical diversity, encompassing distinct land-use zones, facilitates a thorough understanding the effect of industrial, agricultural, and residential activities on groundwater quality within these regions^50^.The use of multivariate analysis further enabled a clearer understanding of the relationships among physicochemical parameters, which is essential for identifying potential contamination sources and conducting a comprehensive assessment of water quality^51^.

### Hydrogeology of the investigated region

**Fig. 2:**
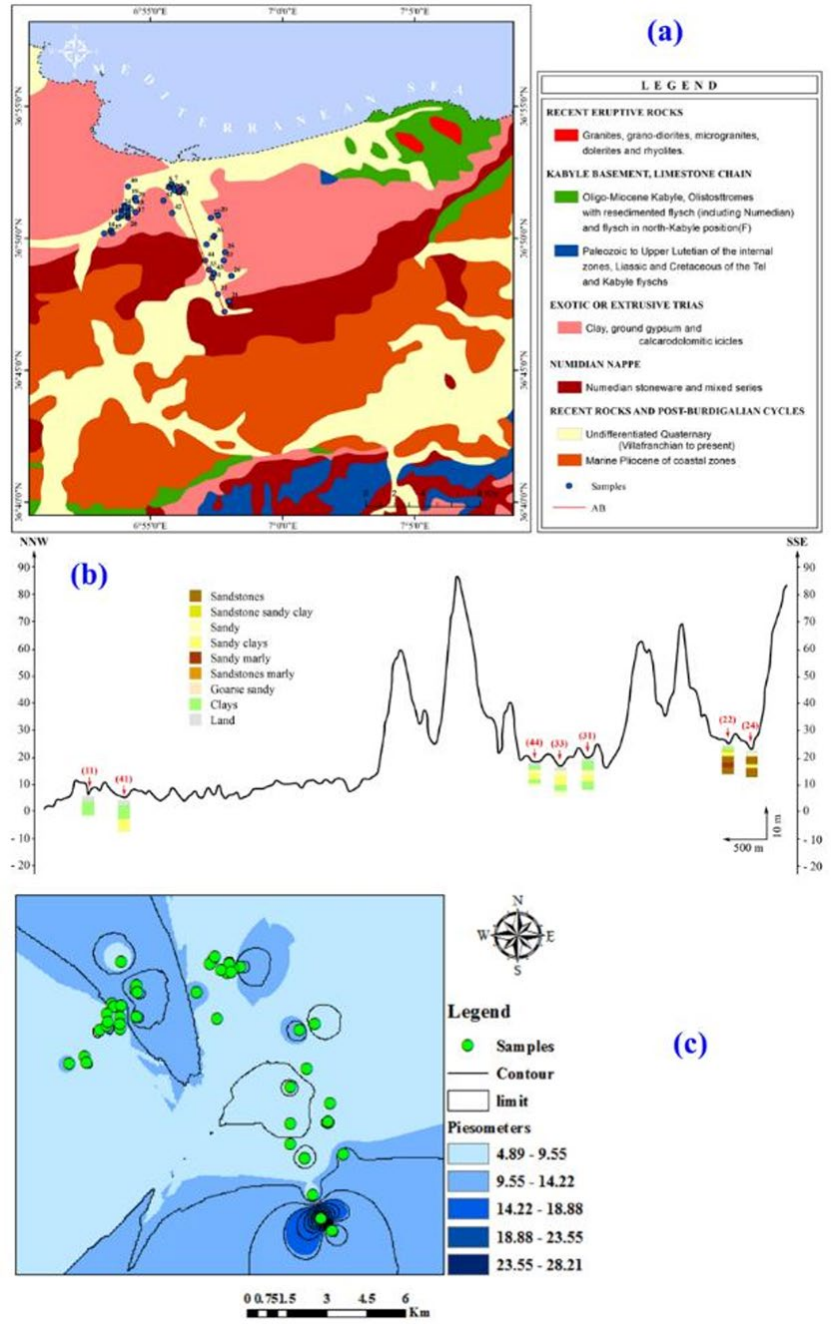
General map including: (a) geological categories, (b) cross section in the study region, and (c) piezometric map. Map 2 (c) created using ArcGIS Pro 2.8.8 Software (Esri; https://www.esri.com/arcgis/about-arcgis).

The Skikda region is located along the northeastern coast of Algeria, which presents a complex and diversified geological framework that governs its groundwater behaviour (Fig. 2). The general geological map (Fig. 2a) reveals that the coastal plains are predominantly covered by Quaternary alluvial and marine formations, corresponding to Villafranchian to recent deposits. These are primarily composed of sandy and clayey materials, providing high porosity and permeability favourable for the development of shallow aquifers^52^. These water-bearing zones are understood to receive water recharge from rainfall infiltration and also obtain water input from adjacent drainage areas.

Moving inland, the geology transitions into the Kabyle basement, featuring Oligo-Miocene flyschs and older Paleozoic to Mesozoic sedimentary sequences, relics of the Maghrebian orogenic chain^53^. These more consolidated rocks generally host semi-confined to confined aquifers, with groundwater flow highly dependent on fractures and structural discontinuities.

The southern part of the study area is dominated by the Numidian nappe, which consists of thick sandstone sequences intercalated with marls and clays. These formations form important aquifer reservoirs, offering high storage capacity but with variable transmissivity, depending on the degree of consolidation and interbedding^54^. Additionally, localized occurrences of Triassic evaporitic formations, rich in gypsum and clays, act as impermeable barriers, locally restricting groundwater flow and creating heterogeneous hydrogeological conditions.

The geological cross-section (Fig. 2b) along the NNW–SSE axis (AB line) provides a detailed visualization of the vertical and lateral variation in lithology. This section highlights a multilayered aquifer system, with alternating permeable units (sandstones, sandy marls, sandy clays) and low-permeability units (clays and marls), a structure that controls groundwater movement and storage^55,56^. The permeable units serve as principal aquifers, enabling significant groundwater flow and storage, while the impermeable or semi-permeable layers act as aquitards or aquicludes, restricting vertical water movement and compartmentalizing the groundwater into semi-confined or confined horizons^55,56^.

Topographically, the region features a gentle elevation gradient, from low-lying coastal zones (0–30 m) to more elevated inland areas (up to 90 m above sea level). This gradient promotes natural groundwater flow from recharge areas inland toward the discharge areas along the coast. The placement of water sampling points (in red) along different geological units further illustrates the hydrogeological heterogeneity, as the interaction between lithology and topography generates variable water quality across the study area.

In essence, the behaviour and availability of groundwater in the Skikda region are fundamentally dictated by its intricate subsurface arrangement, which features a juxtaposition of young, porous sediments, the water-bearing Numidian sandstones, and low-permeability clay layers that act as confining units. A comprehensive grasp of this multifaceted geological framework is therefore indispensable for implementing sound groundwater management strategies and ensuring the long-term viability of this vital resource in the region.

### Collecting of samples and chemical measurements

A total of 44 groundwater samples were collected during two distinct campaigns: one in March 2024 and another in September 2024. In each campaign, all 44 sampling locations were visited, and a sample was taken. Prior to sampling, groundwater was pumped from each well for 15 min to ensure the acquisition of water representative of the aquifer’s actual characteristics, thereby eliminating any stagnant water potentially contaminated or influenced by surface conditions. The collected samples were then filtered in situ using 0.45 μm diameter membranes. The geographical coordinates of each well were recorded using GPS. All sampling procedures were conducted under sterile conditions to minimize the risk of external contamination.

Groundwater samples were collected in pre-cleaned polypropylene (PP) bottles, following the standard guidelines of the American Public Health Association (APHA)^57^. To eliminate potential residues, the bottles underwent a thorough rinsing with distilled water. During fieldwork, initial physicochemical parameters, including temperature, pH, electrical conductivity (EC), turbidity, and total dissolved solids (TDS), were measured in the field using a multiparameter probe (Hach). At each well, the piezometric level was measured using a piezometric probe. The samples were immediately placed in portable coolers to maintain their temperature and prevent chemical reactions or degradation. Subsequently, it stored in a laboratory refrigerator at 4 °C until analysis according to APHA methods^57^.

For detailed chemical analysis in the laboratory, the following techniques were employed: Ca^2+^ and Mg^2+^ were quantified by complexometric titration with EDTA, using a suitable indicator for accurate equivalence point determination. The Cl^–^ and HCO_3_^–^ ions were analysed using conventional volumetric titration methods. Concentrations of Na^+^ and K^+^ were determined by flame photometry, measuring the emitted light intensity of ions exposed to a flame against a calibration curve. The SO_4_^2–^ and NO_3_^–^ ions were quantified using a spectrophotometer, based on light absorption at specific wavelengths after reaction with dedicated chemical reagents, with concentrations determined against a calibration curve. The methodology involved a highly precise execution of each step. For accurate measurement derivation, each sample was subjected to three testing cycles. All analytical results were converted to milliequivalents per litter (meq/L), maintaining an analytical error margin not exceeding 5%.

### Multivariate statistical methods

In this study, multivariate analysis techniques, such as Principal Component Analysis, were used to select the most relevant water quality parameters for developing a reliable index, in line with similar methods used for water quality evaluation.

#### Principal component analysis (PCA)

The PCA is a powerful statistical technique designed to examine and interpret the complex relationships among a large set of variables by reducing dimensionality and highlighting underlying patterns within the data. In this study, PCA was effectively employed to condense numerous water quality parameters into a smaller set of the most influential ones, providing a cost-efficient and robust foundation for the development of a novel WQIs^40–43^. The usefulness of PCA in maintaining the essential variance while simplifying hydrochemical data structures has been broadly recognized in water quality studies of surface and groundwater systems. As a multivariate method, PCA is extensively applied across scientific disciplines to minimize redundancy, extract the most relevant information, and reduce multicollinearity, thereby offering robust insights into complex data structures^54–56^.

Over the past decades, numerous studies have showcased the application of PCA due to its ability to minimize data redundancy, extract the most relevant information, and reduce multicollinearity among variables. As a result, it offers robust insights into complex data structures across various scientific disciplines^58–61^. To determine the possible origins of groundwater constituents and explore the connections between various hydrochemical indicators, PCA was utilized^62^, enabling scientists to penetrate complex datasets and effectively identify the dominant variables and trends that drive the overarching structure of the data.It achieves this by converting a group of related variables into a smaller, uncorrelated set called principal components (PCs). By simplifying datasets, PCA not only clarifies interpretation but also facilitates the design of WQIs tailored to irrigation requirements^63,64^.

Significant relationships between pairs of parameters, often resulting from processes like mineral dissolution or precipitation, were explored using Pearson correlation analysis, offering important understanding of the hydrogeochemical dynamics occurring within the aquifer system^65^. Additionally, PCA is applied to detect and highlight both similarities and differences among samples, while also grouping them based on their hydrochemical characteristics^46^. In groundwater monitoring, such clustering is particularly useful for differentiating natural hydrochemical patterns from those arising due to human activities^11^.

To address the main geochemical processes controlling the chemical composition of groundwater and its quality in Skikda region, meaningful insights were extracted through the application of PCA to the hydrochemical data collected during this study. Comparable approaches in coastal aquifers have demonstrated PCA’s ability to reveal the influence of seawater intrusion and groundwater salinization dynamics, supporting its relevance to the present case study.

#### Cluster analysis (CA)

The CA primarily aims to identify relatively homogeneous groups of objects by assessing their similarities and differences^40–43^. This clustering process involves a step-by-step merging of variables based on their similarity level, culminating in a single cluster, and is visually represented by a dendrogram for intuitive understanding of variable relationships. The fundamental purpose of CA is to establish uniform groupings by analysing object differences and similarities^66^, ensuring items within the same class are grouped by shared traits, a recognized method^67^. Initially, objects sharing common features form small groups, which progressively merge into a single cluster as their similarity increases, effectively distinguishing patterns in groundwater chemistry by accurately grouping samples with similar hydrochemical characteristics into distinct clusters. Ward’s method is the specific cluster analysis technique used here, distinguished by its ANOVA-based quantification of cluster dissimilarity through the sum of squared errors from data points to their cluster centers^68^ to provide a robust approach to cluster analysis and enhance the comprehension of distinct groupings within the water chemistry data of the Skikda region.

### Indexing methods

#### Ions exchanges

The indexing approach is a useful analytical method for tracing the sources and relationships among major ions in groundwater. It employs diagrams such as (Ca + Mg) – (Na + K) versus (HCO_3_ – (SO_4_ + Cl)), (Ca + Mg) versus (HCO_3_), (Ca + Mg) versus (Cl + SO_4_), (Na) versus (Cl), and (Mg/Na) versus (Mg/Ca) to reveal key geochemical interactions. These graphical analyses assist in interpreting the processes that shape the chemical composition of groundwater.

### Irrigation water quality indices (IWQIs)

The IWQIs, initially introduced by^69^ were specifically designed to assess water quality for irrigation and provide a consolidated metric by integrating various physicochemical and biological parameters into a unified quantitative score.

This method was primarily intended to evaluate whether water meets the safety standards before use in farming practices, based on multiple relevant quality indicators^70^. It integrates various physical and chemical water quality indicators into one comprehensive index score, offering a straightforward evaluation of how the water might affect plant development and soil health^71^. Despite their usefulness, WQIs face challenges related to standardization, selection of parameters, and regional adaptation^19^. The reliability of water quality indices largely depends on the weighting of selected parameters, as inappropriate weight allocation may introduce bias into the final index. Conventional methods of weight assignment are typically based on subjective judgment, while factor analysis offers a more rigorous statistical basis for determining objective weights^63^.

Irrigation water can affect soil properties and agricultural productivity, largely driven by the combined influence of multiple water parameters. In this study, key indices including (IWQI, SAR, Na%, PI, MH, and SSP) were determined using the physicochemical properties of the groundwater to assess its quality for irrigation. These indices were calculated using the formulas and classification criteria outlined in Table 1, ensuring a consistent and accurate assessment of the quality of groundwater for irrigation.

**Table 1:** Assessment of groundwater suitability for irrigation purposes.

Water quality indices (WQIs)	Equation	Citation
(IWQI) Irrigation water quality index	${\text{IWQI }} = \mathop \sum \limits_{{{\mathrm{i}} = 1}}^{{\mathrm{n}}} {\mathrm{Q}}_{{\mathrm{i}}} \times {\mathrm{~W}}_{{\mathrm{i}}}$	^72^
(SAR) Sodium adsorption ratio	${\text{SAR }} = \left( {\frac{{{\mathrm{Na}}^{ + } }}{{\sqrt {\frac{{{\mathrm{Ca}}^{{2 + }} + {\mathrm{Mg}}^{{2 + }} }}{2}} }}} \right) \times {\mathrm{~}}100$	^72^
(Na%) Sodium Percent	$\% {\text{Na }} = ~\frac{{\left( {{\mathrm{Na}}^{ + } + {\mathrm{~}}\sqrt {{\mathrm{HCO}}^{{3 - }} } } \right)}}{{\left( {{\mathrm{Ca}}^{{2 + }} + {\mathrm{~Mg}}^{{2 + }} + {\mathrm{~Na}}^{ + } } \right)}} \times {\mathrm{~}}100$	^73^
(MH) Magnesium hazard	${\text{MH }} = \left( {\frac{{{\mathrm{Mg}}^{{2 + }} }}{{{\mathrm{Ca}}^{{2 + }} + {\mathrm{~Mg}}^{{2 + }} }}} \right) \times {\mathrm{~}}100$	^74^
(PI) Permeability index	${\mathrm{PI}}=\left( {\frac{{{\mathrm{N}}{{\mathrm{a}}^+}+\sqrt {{\mathrm{HCO}}_{3}^{ - }} }}{{\left( {{\mathrm{C}}{{\mathrm{a}}^{2+}}+{\mathrm{M}}{{\mathrm{g}}^{2+}}+{\mathrm{N}}{{\mathrm{a}}^+}} \right)}}} \right) \times 100$	^60,71^
(SSP) Soluble sodium percentage	${\text{SSP }} = \frac{{{\mathrm{Na}}^{ + } }}{{{\mathrm{Ca}}^{{2 + }} + {\mathrm{~Mg}}^{{2 + }} + {\mathrm{~Na}}^{ + } }} \times {\mathrm{~}}100$	^75^

The IWQI is established via a formula that uses a non-dimensional score that ranges 0 to 100 in order to evaluate interactions among variables. such as EC, SAR, Na^+^, HCO_3_^−^, and Cl^–^^72^ (Table 2).

1 $${\mathrm{IWQI}}={\mathrm{~}}\mathop \sum \limits_{{{\mathrm{i}}=1}}^{{\mathrm{n}}} {{\mathrm{Q}}_{\mathrm{i}}} \times {\mathrm{~}}{{\mathrm{W}}_{\mathrm{i}}}$$

Q_i_: quality measurement indicating whether it falls within the specified tolerance limits. Conversely, Wi denotes the weight assigned to each variable in the assessment, reflecting its relative significance in the overall evaluation.

2 $${{\mathrm{Q}}_{\mathrm{i}}}={{\mathrm{Q}}_{{\mathrm{max}}}} - \left( {\frac{{[({{\mathrm{X}}_{{\mathrm{ij}}}} - {{\mathrm{X}}_{{\mathrm{inf}}}}) \times {{\mathrm{Q}}_{{\mathrm{imap~}}}}]}}{{{{\mathrm{X}}_{{\mathrm{amp}}}}}}} \right)$$

Where;

X_inf_: lower boundary class. X_ij_: observed variable. Q_imap_: class range and X_amp_: class range in which the variable is positioned.

3 $${{\mathrm{W}}_{\mathrm{i}}}={\mathrm{~}}\frac{{\mathop \sum \nolimits_{{{\mathrm{j}}=1}}^{{\mathrm{K}}} {{\mathrm{F}}_{\mathrm{j}}}{{\mathrm{A}}_{{\mathrm{ij}}}}}}{{\mathop \sum \nolimits_{{{\mathrm{j}}=1}}^{{\mathrm{K}}} \mathop \sum \nolimits_{{{\mathrm{i}}=1}}^{{\mathrm{n}}} {{\mathrm{F}}_{\mathrm{j}}}{{\mathrm{A}}_{{\mathrm{ij}}}}}}$$

Where;

I: physicochemical factors quantity and changing from 1 to n. J: elements number from 1 to k. F: eigenvalue of component. A: extent of parameter i influenced by factor j.

**Table 2:** The predefined range of values for the elements utilized in quality assessment calculation (Q_i_).

Q_i_	EC (µs/cm)	HCO_3_^−^ (meq/L)	Cl^−^ (meq/L)	Na^+^ (meq/L)	SAR
0–35	EC < 200 or EC ≥ 3000	HCO_3_ < 1 or HCO_3_ ≥ 8.5	Cl < 1 or Cl ≥ 10	Na < 2 or Na ≥ 9	SAR > 2 or SAR ≥ 12
35–60	1500 ≤ EC < 3000	4.5 ≤ HCO_3_ < 8.5	7 ≤ Cl < 10	6 ≤ Na < 9	6 ≤ SAR < 12
60–85	750 ≤ EC < 1500	1.5 ≤ HCO_3_ < 4.5	4 ≤ Cl < 7	3 ≤ Na < 6	3 ≤ SAR < 6
85–100	200 ≤ EC < 750	1 ≤ HCO_3_ < 1.5	1 ≤ Cl < 4	2 ≤ Na < 3	2 ≤ SAR < 3

### Machine learning methods for assessing the water quality indices

Conventional WQIs, such as IWQI, SAR, Na%, MR, PI, and SSP, suffer from a significant drawback: their calculation requires expert weighting of variables, often leading to ambiguous results^38^. These indices aggregate diverse physicochemical parameters into a single value representing water suitability. To reduce subjectivity, researchers have incorporated entropy-based critical ion weights, enhancing measurement accuracy. Nevertheless, traditional estimation methods relying on mathematical equations require extensive data collection, laboratory analysis, administration, and testing, making them inherently time-consuming and labor intensive. On the other hand, machine learning algorithms (MLAs) offer a more efficient alternative^76^.

This study developed three MLAs, specifically Random Forest Regression (RF), Extreme Gradient Boosting Regression (XGBR), and Adaptive Boosting Regression (ABR) using Python’s scikit-learn package within the Spyder environment. These models predict WQIs using selected physiochemical parameters as input features. Crucially, model hyper-parameters settings predetermined before training and not learned from the data play a vital role in determining model performance^77–79^.

#### Random forest regression (RF)

Random Forest (RF) is an ensemble MLA widely used for regression and classification tasks. It operates by constructing multiple decision trees during training and aggregating its predictions to improve accuracy and robustness. Each tree is trained on a random subset of the data (bootstrap aggregation or bagging). In this study, two key hyperparameters were optimized to mitigate overfitting, a critical risk given the limited sample size: the number of trees in the forest (tested range: 1 to 20) and the maximum depth allowed for any individual tree (tested range: 1 to 10 levels). The mean squared error (MSE) was used as the criterion to evaluate the quality of splits within each tree. As established in the literature^80^, the final RF prediction for regression is obtained by averaging the predictions of all individual trees. This ensemble approach significantly enhances predictive performance and generalization capability on unseen data.

#### Adaptive boosting regression (ABR)

ABR is a sequential ensemble technique adapted from the AdaBoost algorithm, originally designed to enhance classification and regression trees^81^. Its core principle involves iteratively training a sequence of weak learners (typically simple models) on adaptively reweighted versions of the training data. The algorithm proceeds as follows: Each training instance is assigned an initial weight indicating its relative importance. In each iteration, a weak learner is trained on a dataset derived by resampling the original data according to the current instance weights. The learner’s error is computed, heavily influenced by mis-predicted instances with high weights. Instance weights are then updated: weights for instances poorly regressed by the current learner are increased, focusing subsequent iterations on the most challenging cases^82^. Adapting AdaBoost to regression requires handling real-valued errors instead of binary classification outcomes. ABR achieves by: Comparing prediction errors against a threshold to define an “error” for weight update purposes, similar to the classification approach. Modifying resampling probabilities based on the magnitude of the error; instances with larger errors have a higher probability of selection in the next iteration. Combining the predictions of all weak learners in the sequence using either a weighted average or the median to produce the final regressor^83,84^. For this study, ABR hyperparameters were optimized: number of sequential weak learners (tested range: 1 to 20) and maximum depth of the base decision tree learners (tested range: 1 to 10 levels).

#### Extreme gradient boosting regression (XGBR)

XGBR is a highly optimized and scalable implementation of the gradient boosting framework. Gradient boosting is an ensemble technique that builds models sequentially; each new decision tree is trained to correct the residual errors of the preceding ensemble. XGBR specifically enhances this process by utilizing gradient descent optimization to minimize a specified differentiable loss function. The model constructs an ensemble of decision trees acting as base learners. A core principle is that these learners are designed to make uncorrelated errors on different data subsets. When their individual predictions are combined, these errors compensate for one another, resulting in significantly improved overall prediction accuracy^77,85,86^. To optimize the XGBR model’s performance for this study, the following key hyperparameters were systematically tuned to mitigate overfitting, a critical risk given the limited sample size: (i) learning rate controls the contribution of each tree to the ensemble (tested values: 0.1, 0.01, 0.001). (ii) The number of boosting rounds (i.e., decision trees) in the sequence (tested range: 1 to 20). (iii) The maximum depth permitted for each individual decision tree, crucial for managing model complexity and preventing overfitting (tested range: 1 to 20 levels). This comprehensive exploration of hyperparameter space aimed to identify the optimal configuration maximizing predictive performance.

#### SHAP implementation

While conventional MLAs identify which input features influence outputs, it often fail to elucidate how these features contribute to specific predictions^87^. To address this interpretability gap, advanced techniques like SHAP analysis has emerged, leveraging game-theoretic principles to explain complex “black-box” models^88,89^. SHAP provides a unified framework for model interpretation based on three key properties: (i) local Accuracy, the explanation matches the model’s output for the specific instance being explained. (ii) missingness, features not present in the instance contribute nothing. (iii) consistency, if a feature’s impact increases in any model, its SHAP value does not decrease.

This framework quantifies the exact contribution (Shapley value) of each feature to an individual prediction by treating features as cooperative players in a game^90^. Consequently, SHAP analysis not only ranks global feature importance but also reveals the directionality (positive or negative) and magnitude of each feature’s influence on every prediction, significantly enhancing model transparency^91^.

#### Implementation of the planned approaches

**Fig. 3:**
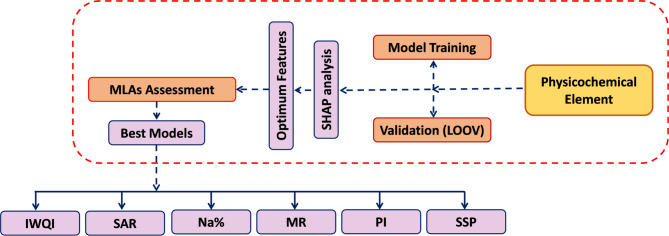
Flowchart for predicting WQIs, such as IWQI, SAR, Na%, MR, PI, and SSP using different MLAs based on various physicochemical element.

This study proposes the use of MLAs specifically RF, ABR, and XGBR to enhance the monitoring of WQIs. As outlined in Fig. 3, our methodology as following: (a) Various physicochemical parameters are used as model inputs. (b) The MLAs are trained using Leave-One-Out Cross-Validation (LOOCV), with their hyperparameters systematically optimized. (c) SHAP analysis is applied to interpret the impact of each parameter on the predicted WQIs and to identify the top five most predictive features. (d) The models are then retrained using only these top five features and their previously identified optimal hyperparameters to produce the final, best-performing versions. The python code used to implement this scheme is provided in the Supplementary materials (Table S1).

#### Datasets and software for data analysis

The location of the collected groundwater samples (Fig. 1) and the spatial distribution of the calculated water quality indices (WQIs) were visualized on maps (Figs. 1 and 2 (c), 8, and 9) to demonstrate the extremely risky and safe regions in terms of water quality. These maps were produced with the ArcGIS Pro 2.8.8 software (Esri; https://www.esri.com/arcgis/about-arcgis). Moreover, leave-one-out cross-validation (LOOCV) method was applied for enhancing the model robustness and minimizing the overfitting. In each iteration, LOOCV designates a single sample as the validation set and calibrates the model on the remaining data. This method leverages nearly the entire dataset for calibration each time, providing a rigorous assessment of predictive performance^92,93^. For hyperparameter optimization, GridSearchCV was used to systematically evaluate parameter combinations, selecting the best-performing model based on the highest R² and lowest RMSE. The optimized hyperparameters for each model are detailed in Table 3. To ensure reproducibility and control randomness, the dataset was partitioned using a fixed random state. This controlled approach maintains consistency in node splitting, reduces sampling variability, and enhances result reliability.

**Table 3:** Applied models and their hyperparameters based on summer and winter data.

WQIs	Models	Hyperparameter’s summar	Hyperparameter’s winter
IWQI	RF	D: 5, T: 13	D: 2, T: 19
	ABR	α: 0.1, T: 12	α: 0.1, T: 12
	XGBR	α: 0.05, D: 3, T: 20	α: 0.05, D: 3, T: 20
SAR	RF	D: 8, T: 13	D: 6, T: 16
	ABR	α: 0.1, T: 7	α: 0.1, T: 19
	XGBR	α: 0.05, D: 2, T: 20	α: 0.05, D: 8, T: 20
Na%	RF	D: 8, T: 9	D: 7, T: 5
	ABR	α: 0.01, T: 3	α: 0.01, T: 4
	XGBR	α: 0.05, D: 4, T: 20	α: 0.05, D: 3, T: 20
MR	RF	D: 7, T: 9	D: 5, T: 14
	ABR	α: 0.01, T: 10	α: 0.01, T: 18
	XGBR	α: 0.05, D: 4, T: 20	α: 0.05, D: 3, T: 20
PI	RF	D: 7, T: 19	D: 7, T: 3
	ABR	α: 0.1, T: 8	α: 0.1, T: 20
	XGBR	α: 0.05, D: 3, T: 20	α: 0.05, D: 4, T: 20
SSP	RF	D: 9, T: 15	D: 7, T: 9
	ABR	α: 0.1, T: 12	α: 0.01, T: 13
	XGBR	α: 0.05, D: 5, T: 20	α: 0.05, D: 6, T: 20

D, T, and α represent the maximum depth of individual trees, the number of trees in the forest, learning rate.

#### Models evaluation

Regression models were evaluated the performance using the following statistical indicators: root mean square error (RMSE), mean absolute error (MAE), Nash-Sutcliffe efficiency (NSE), Willmott index (WI), and the coefficient of determination (R^2^) (Eqs. 4–18). The optimal model was selected based on maximizing NSE, WI, and R^2^ while minimizing MAE and RMSE. The parameters are defined as: Y_a_ is the actual value, Y_p_ is the predicted value, Ȳ is the mean of actual values and N is the number of data points.

4 $${\mathrm{MAE}}=\frac{1}{{\mathrm{N}}}\mathop \sum \limits_{{{\mathrm{i}}=1}}^{{\mathrm{N}}} \left| {{{\mathrm{Y}}_{\mathrm{a}}} - {{\mathrm{Y}}_{\mathrm{p}}}} \right|$$

5 $${\mathrm{RMSE}}=\sqrt {\frac{1}{{\mathrm{N}}}\mathop \sum \limits_{{{\mathrm{i}}=1}}^{{\mathrm{N}}} {{\left( {{{\mathrm{Y}}_{\mathrm{a}}} - {{\mathrm{Y}}_{\mathrm{p}}}} \right)}^2}}$$

6 $${\mathrm{NSE}} = 1 - \left[ {\frac{{\mathop \sum \nolimits_{{i = 1}}^{N} \left( {{\mathrm{Y}}_{{\mathrm{a}}} - {\mathrm{Y}}_{{\mathrm{p}}} } \right)^{2} }}{{\mathop \sum \nolimits_{{i = 1}}^{N} \left( {{\mathrm{Y}}_{a} - \bar{Y}} \right)^{2} }}} \right]$$

7 $${\mathrm{WI}} = 1 - \left[ {\frac{{\mathop \sum \nolimits_{{i = 1}}^{N} \left( {{\mathrm{Y}}_{{\mathrm{a}}} - {\mathrm{Y}}_{{\mathrm{p}}} } \right)^{2} }}{{\mathop \sum \nolimits_{{i = 1}}^{N} \left( {\left| {{\mathrm{Y}}_{{\mathrm{a}}} - \bar{Y}} \right| + \left| {{\mathrm{Y}}_{{\mathrm{p}}} - \bar{Y}} \right|} \right)^{2} }}} \right]$$

8 $${\mathrm{R}}^{2} = \frac{{\sum \left( {{\mathrm{Y}}_{{\mathrm{a}}} - {\mathrm{Y}}_{{\mathrm{p}}} } \right)^{2} }}{{\sum \left( {{\mathrm{Y}}_{{\mathrm{a}}} - \bar{Y}} \right)^{2} }}$$

## Results and discussion

### Hydrochemical features

The evaluation of water quality parameters in the Skikda region, assessed against established irrigation standards^94^, revealed significant seasonal shifts in the groundwater’s hydrochemical characteristics between the summer (dry period) and winter (wet period) (Table 4). The pH levels exhibited relative stability, with a mean of 7.40 (range 6.52–7.86) during the dry period and a slightly lower mean of 7.07 (range 6.25–9.29) during the wet period. These values generally fall within the acceptable FAO range of 6.0 to 8.5, considered favourable for most crops89. However, the recorded maximum pH of 9.29 during the wet season suggests the presence of localized alkalinity, potentially stemming from intensified water-rock interactions or the enhanced dissolution of carbonate minerals due to increased water flow during recharge events. This alkalinity, if persistent, could affect the availability of certain nutrients to plants.

In contrast to the relatively stable pH, Electrical Conductivity (EC) showed consistently high average values in both periods: 1880.91 µS/cm (range 532.00–5830.00 µS/cm) during the dry season and 1715.52 µS/cm (range 730.00–4074.00 µS/cm) during the wet season. Alarmingly, the maximum EC values in both periods surpassed the FAO threshold of 3000 µS/cm, unequivocally indicating significant salinity issues that could severely restrict the types of crops suitable for irrigation. This elevated salinity is likely exacerbated by the intensification of agricultural activities and subsequent irrigation practices, leading to the leaching of accumulated salts from the soil profile into the underlying groundwater^90,91^. Furthermore, natural processes such as prolonged water-rock interaction, particularly with salt-bearing geological formations, and anthropogenic influences, including industrial and agricultural practices introducing dissolved solids, likely contribute to this salinity^92–94^. Our results confirm that salinization is a complex process influenced by hydrogeological mechanisms, as documented in studies modeling this phenomenon^95^, and it represents a major challenge for agriculture in coastal regions .

Similarly, Total Dissolved Solids (TDS) mirrored the EC trends, averaging 973.84 mg/L (range 258.00–3020.00 mg/L) in the dry period and 950.82 mg/L (range 393.00–2360.00 mg/L) in the wet period, with maximum concentrations exceeding the FAO limit of 2000 mg/L in several samples, further reinforcing the substantial salinity concern and its potential to cause osmotic stress in plants, hindering water uptake. Similar salinity-related challenges have been observed in other Algerian semi-arid regions, particularly in the Dzira agricultural district of the Ksour Mountains, where EC values ranged from 1520 to 3620 µS/cm and TDS levels frequently exceeded 2000 mg/L, making these parameters the primary constraints on irrigation suitability Meziani et al. (2024). This situation closely mirrors our results in Skikda, where groundwater salinization similarly represents a dominant limiting factor for agricultural use, reinforcing the notion that salinity is a pervasive issue across Algerian aquifers and a critical challenge for sustainable water resource management.

Chloride (Cl^–^) concentrations displayed a notable seasonal variation. During the dry period, the average concentration was 288.72 mg/L (range 30.90–1280.00 mg/L); however, the persistence of maximum values exceeding FAO limits suggests the continued presence of localized saline sources, possibly linked to the interaction of groundwater with evaporitic formations and the seepage of industrial and urban wastewater into the water system .Conversely, the average chloride concentration increased to 876.00 mg/L (range 73.00–1280.00 mg/L) during the wet period, with maximum values exceeding the FAO guideline of 1036.00 mg/L, potentially attributable to the leaching of naturally occurring saline soils due to increased recharge, flushing accumulated salts into the groundwater, or the influence of the nearby coastal areas if the aquifer system exhibits hydraulic connectivity with seawater, leading to saline intrusion. These results indicate that seawater intrusion is an important factor influencing groundwater dynamics, as demonstrated by studies that have evaluated this phenomenon in coastal aquifers^96^, consistent with recent stochastic modeling approaches that emphasize the variability of saline groundwater in coastal aquifers^97^. These elevated chloride levels can be toxic to sensitive crops, causing leaf burn and reduced yields.

Sulfate (SO_4_^2–^) concentrations remained generally within the acceptable FAO limit of 960 mg/L in both seasons, averaging 128.39 mg/L (range 1.00–345.00 mg/L) in the dry period and 140.05 mg/L (range 4.20–429.15 mg/L) in the wet period, with sulfate often originating from the oxidation of sulfides and the weathering of evaporitic rocks such as gypsum^98^.

Nitrate (NO_2_^–^) levels were consistently low across both seasons, averaging 0.81 mg/L (range 0.18–2.99 mg/L) in the dry period and 0.79 mg/L (range 0.03–6.60 mg/L) in the wet period, suggesting minimal anthropogenic nitrate contamination, with the presence of nitrate generally attributed to agricultural (nitrogen fertilizers) and domestic activities^99^ (FAO limit: 10 mg/L – within safe range).This finding reinforces the notion of minimal anthropogenic influence, in line with multivariate assessments that have traced potential pollution sources.

Bicarbonate (HCO_2_^–^) concentrations, contributing to the alkalinity of the groundwater, averaged 370.40 mg/L (range 198.00–695.00 mg/L) in the dry period and 341.73 mg/L (range 201.00–610.00 mg/L) in the wet period, likely influenced by the carbonate dissolution; high bicarbonate levels may affect the soil pH and nutrient availability (FAO limit: 610 mg/L – some exceedances observed).

The average concentrations of major cations, including Calcium (Ca^2+^) (dry: 100.23 mg/L, wet: 104.52 mg/L), Magnesium (Mg^2+^) (dry: 52.09 mg/L, wet: 115.00 mg/L), Sodium (Na^+^) (dry: 172.70 mg/L, wet: 191.73 mg/L), and Potassium (K^+^) (dry: 6.10 mg/L, wet: 9.16 mg/L), were generally within the typical ranges observed in groundwater and reflect the dominant cation composition likely derived from the weathering of the local geological formations(FAO limits: Ca^2+^ 400 mg/L, Mg^2+^ 200 mg/L, Na^+^ 919 mg/L, K^+^ 400 mg/L – all within safe range). Notably, the dry period exhibited higher coefficients of variation for several ions, indicating greater spatial heterogeneity in water quality, suggesting that different sources and processes become more dominant under low flow conditions. The lower magnesium levels observed during the dry period could potentially be linked to the precipitation of carbonate minerals as alkalinity increases, potentially affecting the availability of this essential plant nutrient^100^. Sodium and potassium concentrations are often attributed to the natural dissolution of feldspars present in the aquifer matrix, as well as anthropogenic intrusions, particularly the use of agricultural fertilizers and the discharge of wastewater runoff^101^ (FAO guideline confirms values are acceptable, though SAR must be considered). Elevated sodium levels, even at these averages, can pose a risk to soil structure over time, especially if the Sodium Adsorption Ratio (SAR) is high, which requires consideration of calcium and magnesium concentrations.

Skikda’s groundwater exhibits a dynamic hydrochemical system shaped by seasonal changes, natural processes, and human influences. Consistently high salinity and elevated chloride, particularly in the wet season, significantly limit its direct use for irrigation. Effective management, including salt-tolerant crops and soil leaching, is crucial. The dry season’s increased spatial variability necessitates site-specific water quality assessments for sustainable agriculture.

**Table 4:** Physiochemical analysis of groundwater samples in the wet and dry period (2024).

	pH	EC	TDS	Mg^2+^	Ca^2+^	K^+^	Na^+^	Cl^−^	SO_4_^2−^	HCO_3_^−^	NO_3_^−^
Summer period (*n* = 44)											
Min	6.52	532	258	10	40	0.66	62.2	30.9	1	198	0.18
Mean	7.4	1880.91	973.84	52.09	103.25	6.1	172.7	288.72	128.39	370.4	0.81
Max	7.86	5830	3020	167	366	23.32	510.54	1280	345	695	2.99
ST Dev	0.36	933.8	479.7	31.84	59.92	6.01	170.8	243.72	61.13	123.84	0.53
CV (%)	4.86	49.65	49.26	61.12	58.03	98.52	98.90	84.41	47.61	33.43	65.43
FAO Limit	8.5	3000	2000	2	919	6	400	1036	960	610	10
Winter period (*n* = 44)											
Min	6.25	730	393	18.2	51	0.4	69.82	73	4.2	201	0.12
Mean	7.07	1715.52	950.82	115	104.52	9.16	191.73	876	140.05	341.73	0.79
Max	9.29	4074	2360	167	232	45.6	438	1800	429.15	610	2.34
ST Dev	0.54	624.19	375.51	18.65	37.84	9.9	206.26	186.6	82.01	94.97	0.88
CV (%)	7.64	36.38	39.49	16.22	36.20	108.08	107.58	21.30	58.56	27.79	111.39
Data across two period (*n* = 88)											
Min	6.25	532.00	258.00	10.00	40.00	0.40	62.20	30.90	1.00	198.00	0.12
Mean	7.24	1798.22	962.33	83.55	103.89	7.63	182.22	582.36	134.22	356.07	0.80
Max	9.29	5830.00	3020.00	167.00	366.00	45.60	510.54	1800.00	429.15	695.00	2.99
ST Dev	0.13	218.93	73.67	9.33	15.61	2.75	25.07	40.39	14.76	20.41	0.25
CV (%)	1.76	12.17	7.66	11.16	15.03	36.05	13.76	6.94	11.00	5.73	30.94

#### Groundwater facies

**Fig. 4:**
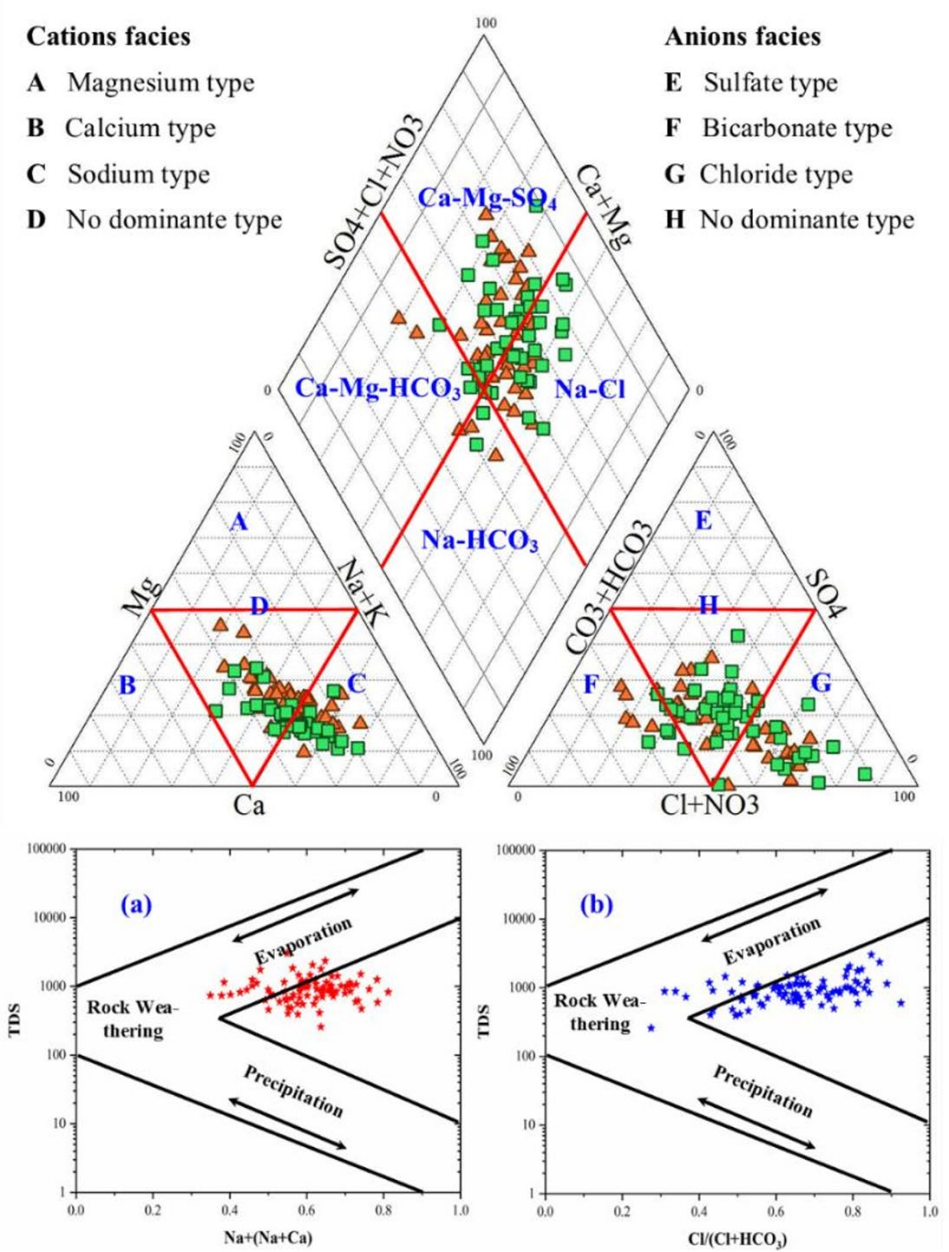
Groundwater evolution in Morang based on (a) Piper diagram and (b) Gibbs plots.

Groundwater in the Skikda region was systematically classified into hydrochemical facies by ranking major ions. This research utilized various charts to visualize complex datasets, clarify spatial and temporal variations in key hydrochemical parameters, and comprehensive assessment of groundwater quality. Analysing the Skikda region’s groundwater using the Piper diagram^102^ (Fig. 4) reveals two principal chemical water facies: Calcium-Magnesium-Sulfate (Ca-Mg-SO_4_) and Sodium-Chloride (Na-Cl). The distinction between these facies is primarily based on the dominant anions as the following: (a) Ca-Mg-SO_4_ type which is characterized by a higher relative abundance of sulfate, and (b) Na-Cl type which showed a greater proportion of chloride. Notably, across both facies, sodium tends to be the most prevalent cation in the majority of the analysed groundwater samples. The occurrence of certain samples exhibiting a mixed water-type suggests a complex aquifer system where waters with differing geochemical histories or compositions are undergoing mixing processes^12^. This blending could occur due to varying flow paths within the aquifer or potential hydraulic connections between different zones.

Further investigation using the Gibbs diagram (Fig. 4a and b) revealed the effect of evaporation processes as the primary mechanism governing the overall chemical composition of the Skikda groundwater. Evaporation in aquatic systems, such as rivers and estuaries, acts as a concentrating mechanism, which leads to a progressive increase in dissolved salt concentrations and consequently enhances salinity over time^6^. The concentration of the majority of samples within the evaporation dominance field indicates that the loss of water through evaporation, leading to an increase in the concentration of dissolved salts in the remaining water that subsequently infiltrates into the ground, which is a key factor shaping the groundwater’s chemistry. This process effectively concentrates ions that were initially present in lower amounts. However, the presence of a few samples exhibiting a slight trend towards the rock weathering field suggests that the dissolution of minerals composing the aquifer matrix also contributes to the suite of dissolved ions present in the groundwater, although its influence appears to be secondary to the concentrating effect of evaporation^103^. This mineralization process driven by the dissolution of carbonates, gypsum, and other evaporitic minerals, has been recognized as a major source of calcium, magnesium, sulfate, and bicarbonate in groundwater^11,95^. This indicates a dual control on groundwater chemistry, with evaporation being the dominant process and mineralization providing an additional baseline contribution of dissolved constituents. The combined influence of evaporation and secondary rock weathering on groundwater chemistry is consistent with stochastic modeling studies that highlight the interaction between climatic and geological factors in saline groundwater formation^97^. This indicates a dual control on the groundwater chemistry, with evaporation being the more dominant process in determining the overall ionic strength and composition, while water-rock interactions contribute a baseline level of dissolved constituents. Comparable hydrochemical patterns have also been observed in the watershed of Fetzara Lake in Northeast Algeria, where Na–Cl, Ca–Mg–Cl, and Ca–Mg–HCO₃ facies dominate surface waters depending on their origin. In that system, evaporation and silicate weathering were likewise identified as the primary processes controlling mineralization, with cation exchange playing a secondary role^104^. These findings mirror the Skikda aquifer, where Na–Cl and Ca–Mg–SO₄ facies dominate and evaporation emerges as the principal driver of groundwater chemistry, while mineral dissolution and ion exchange provide complementary contributions. Such parallels emphasize that in both coastal and inland hydro systems of Northeast Algeria, water quality is strongly shaped by the interplay of evaporation and rock–water interactions.

### Ion exchange processes

The hydrochemical investigation and the interpretation of multiple graphical representations revealed a nuanced and intricate characterization of groundwater quality in the Skikda region, which located within Skikda Province, Algeria. The varied dispersion of sampling points across these diagrams highlights the influence of multiple controlling factors on the ionic composition of the groundwater. These include complex interactions between water and geological formations, hydrogeological dynamics, and possible anthropogenic inputs. The region’s coastal proximity suggests that climatic conditions and marine influences, including potential seawater intrusion, may also play a significant role in shaping the hydrochemical profile. Intensive extraction of groundwater from coastal aquifers lowers the freshwater pressure, enabling seawater to encroach into the aquifer, a phenomenon known as saltwater intrusion^105^. The Skikda area, such mechanisms are likely to affect groundwater composition, particularly in locations adjacent to the Mediterranean coastline.

**Fig. 5:**
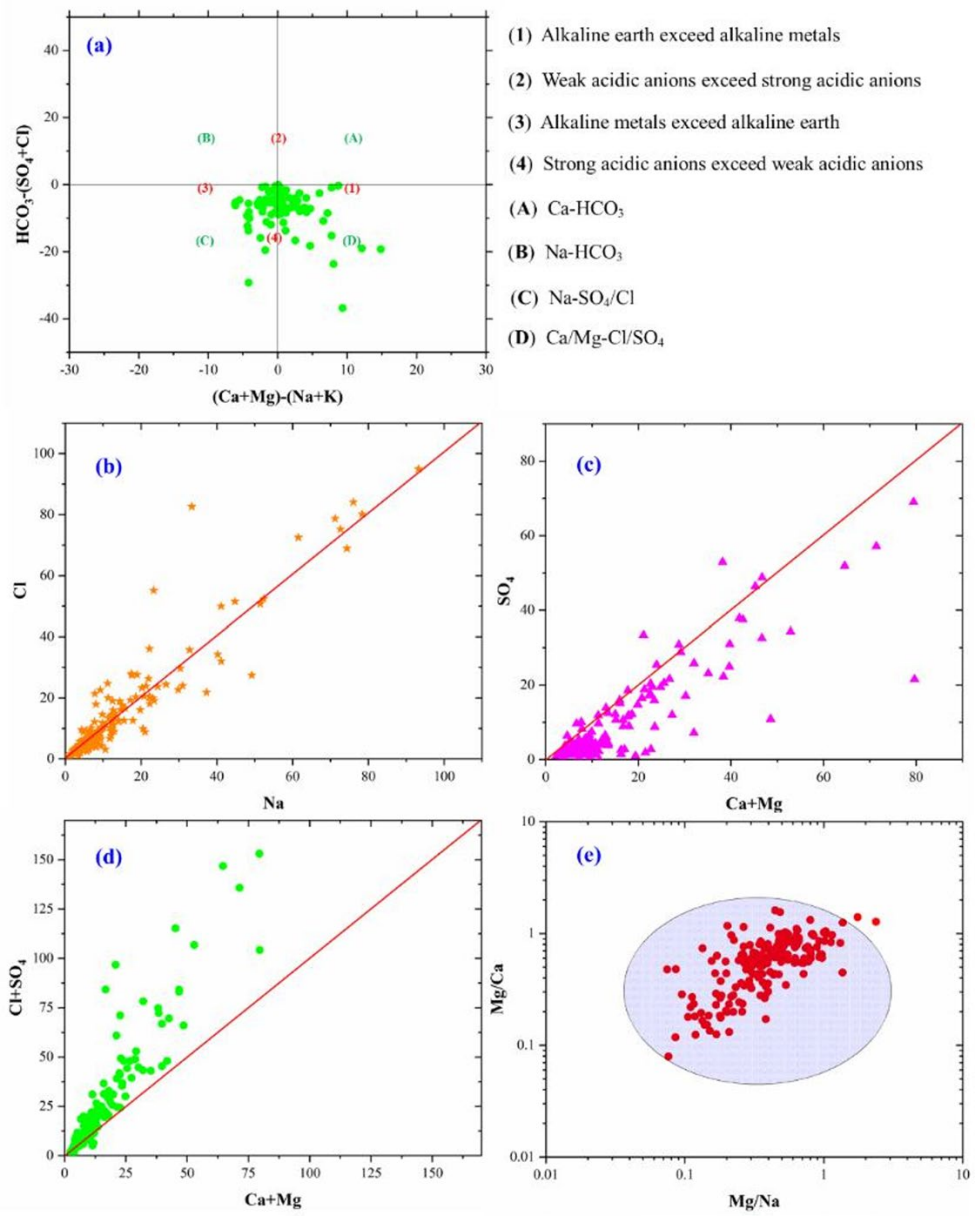
Mechanism controlling groundwater chemistry in Morang aquifer system based on ionic ratios: (a) (Ca + Mg) – (Na + K) vs. (HCO_3_) – (SO_4_ + Cl), (b) Na vs. Cl, (c) (Ca + Mg) vs. SO_4_ (d) (Ca + Mg) vs. (Cl + SO_4_), and (e) Mg/Na vs. Mg/Ca.

The complex hydrochemical properties of groundwater are comprehensively illustrated through the Durov diagram (Fig. 5a) and a series of informative scatter plots (Fig. 5b-e). The Durov diagram distinctly reveals that a significant majority of the groundwater samples are situated within field (1), a domain signifying that the concentration of alkaline earth metals (specifically Calcium (Ca^2+^) and Magnesium (Mg^2+^)) surpasses that of alkali metals (Sodium (Na^+^) and Potassium (K^+^)), and simultaneously, the concentration of bicarbonate (HCO_2_^–^) exceeds that of strong acidic anions (Sulfate (SO_4_^2–^) and Chloride (Cl^–^).

However, the Durov diagram also reveals a noteworthy presence of groundwater samples plotting within field (2). This field represents water where alkaline earth metals still dominate over alkali metals, but crucially, the strong acidic anions (Sulfate and Chloride) exceed bicarbonate. The presence of these samples indicates the existence of mixed water types within the aquifer, where the influence of other geochemical processes, beyond simple carbonate dissolution, is significant. Mixed groundwater types can result from gypsum and anhydrite dissolution, cation exchange processes, and anthropogenic influences such as agricultural and industrial activities^6,51^. These mixed waters likely reflect contributions from different geological sources or the impact of specific hydrogeochemical reactions that enrich the water in sulfate and chloride ions.

The accompanying scatter plots provide further granular insights into the relationships and potential sources of these key ions. Figures (5b) displays the correlation between Sodium (Na^+^) and Chloride (Cl^–^). The observed general positive trend suggests a potential contribution from the dissolution of halite (common salt) or the influence of saline water intrusion into parts of the aquifer. However, the considerable scatter around this trend indicates that other processes, i.e., silicates weathering or anthropogenic inputs, e.g., agricultural runoff, wastewater discharge, or industrial activities, also contribute to the overall Sodium and Chloride concentrations^47,106^.

Figure 5c examines the relationship between the sum of alkaline earth metals (Ca^2+^ + Mg^2+^) and Sulfate (SO_4_^2–^). The general positive trend here hints at a possible contribution from the dissolution of sulfate-bearing minerals like gypsum or anhydrite within the aquifer matrix. Nevertheless, the noticeable dispersion of data points suggests that the sources and behaviour of Calcium, Magnesium, and Sulfate are not solely controlled by this single process, and other factors, such as cation exchange or the dissolution of other mineral phases, likely play a role.

Figure 5d, plotting the sum of alkaline earth metals (Ca^2+^ + Mg^2+^) against the sum of strong acidic anions (Cl^–^ + SO_4_^2–^), reinforces the presence of mixed water types. The broader positive correlation indicates that as the concentration of alkaline earth metals increases, so too does the concentration of the stronger anions, highlighting the interplay of different geochemical processes contributing these ions to the groundwater. The absence of a tight linear trend suggests overlapping sources and variable geochemical pathways contributing to groundwater composition in the Skikda region.

Finally, Fig. 5e, the cross-plot of Mg/Ca versus Mg/Na ratios, offers valuable information regarding the hydrochemical evolution of the groundwater. The tight clustering of a significant portion of the data points within a defined region suggests a common evolutionary pathway for these waters. The relatively low Mg/Na ratios, in conjunction with the more variable Mg/Ca ratios, could be indicative of dominant carbonate dissolution (which typically releases more Calcium than Magnesium) coupled with potential cation exchange processes, where Calcium in the water might be exchanged for Magnesium or Sodium on the aquifer materials. Supporting the interpretation of natural versus human-induced influences in the Skikda aquifer. In the case of the Skikda region, the noticeable clustering of most samples within or near the highlighted blue oval indicates a shared geochemical origin or process influencing ion proportions in a majority of groundwater samples.

### Statistical analysis for ion source detection

#### Clustering approach

**Fig. 6:**
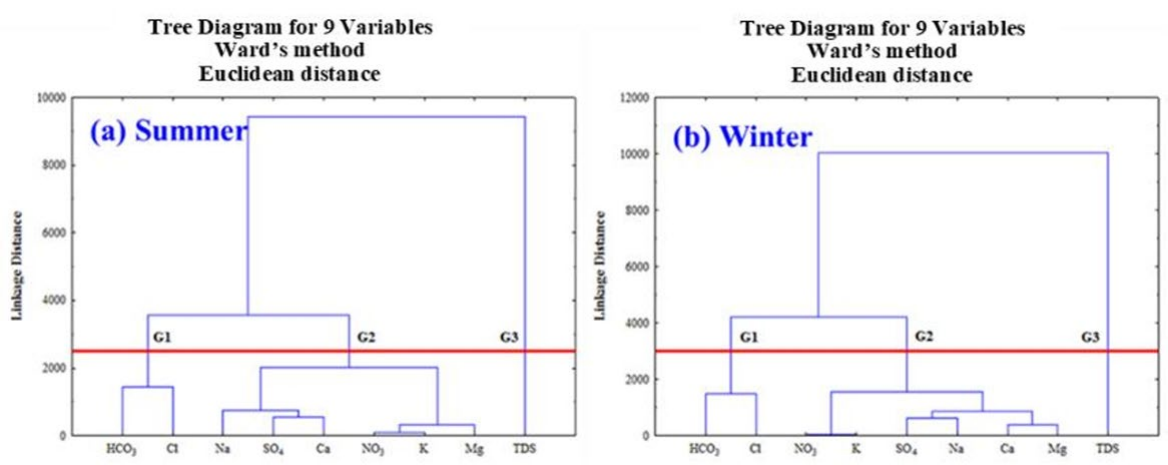
The number of clusters or groups extracted from dendrogram branches.

Hierarchical Cluster Analysis (HCA) in Skikda revealed distinct seasonal associations among groundwater quality parameters, forming three clusters in both summer and, winter indicating a dynamic hydrochemical system. Similarly, dendrogram or Cluster Analysis (CA) in Mornag, considering physicochemical factors (Mg^2+^, Ca^2+^, K^+^, Na^+^, Cl^–^, HCO_3_^–^, NO_3_^–^, SO_4_^2–^, TDS), also identified three distinct groupings for sample classification and correlation understanding (Fig. 6). This similarity with previous studies suggests that seasonal clustering of groundwater parameters is a reproducible feature in aquifer systems, reflecting common hydrogeochemical controls, recharge patterns, and anthropogenic influences (1, 2, 6, 8 12). In summer, Cluster G1 again linked HCO_3_^–^ (Bicarbonate) and Cl^–^ (Chloride). Cluster G2 included Na⁺ (Sodium), SO_4_^2–^ (Sulfate), Ca^2+^ (Calcium), and NO_3_^–^ (Nitrate), suggesting different co-variation patterns compared to winter, potentially due to increased evaporation or altered anthropogenic influences^107^. Cluster G3 grouped K^+^ (Potassium), Mg^2+^ (Magnesium), and TDS (Total Dissolved Solids), indicating a closer relationship between these parameters during the drier, warmer period, possibly driven by overconcentration or specific mineral dissolution (Fig. 6a).

In winter, Cluster G1 grouped HCO_3_^–^ (Bicarbonate) and Cl^–^ (Chloride), suggesting shared influences on their concentrations. Cluster G2 comprised NO_3_^–^ (Nitrate), K^+^ (Potassium), SO_4_^2–^ (Sulfate), Na^+^ (Sodium), Ca^2+^ (Calcium), and Mg^2+^ (Magnesium), indicating common hydrogeochemical controls during the wetter period. TDS (Total Dissolved Solids) formed its own distinct Cluster G3, implying its overall variation is influenced by a broader set of factors than specific ionic associations (Fig. 6b).

These seasonal shifts in variable clustering underscore the evolving hydrogeochemical processes within the Skikda aquifer, highlighting the importance of considering seasonal dynamics for effective water resource management and targeted monitoring strategies. The consistent association of Bicarbonate and Chloride suggests a stable underlying relationship, while the changing composition of other clusters reflects the influence of varying recharge, water-rock interaction, and anthropogenic activities across seasons.

#### Principal component method

Earlier studies have shown that Principal Component Analysis (PCA) serves as an effective tool for condensing numerous physicochemical parameters into a smaller set of dominant variables^64^ .This method underscores the value of PCA in simplifying complex datasets while preserving the most significant information, thus offering a more objective and economical approach to water quality evaluation. In the present study, PCA was applied to groundwater in the Skikda region to identify the main processes influencing water chemistry and to distinguish between natural and anthropogenic sources of variability. Earlier applications of PCA in water quality studies have demonstrated its usefulness for disentangling natural from anthropogenic influences. This approach has been shown to highlight the combined impacts of agricultural inputs, industrial discharges, and domestic wastewater on water quality dynamics. Integrating statistical and computational methods enabled the determination of the key natural and human-induced factors affecting groundwater contamination^18^, providing a robust framework for interpreting the main drivers of water quality variability in the Skikda region. Such findings emphasize the ability of PCA not only to reduce dimensionality but also to trace pollution sources, a perspective that is directly relevant to groundwater assessment in the Skikda region.

**Fig. 7:**
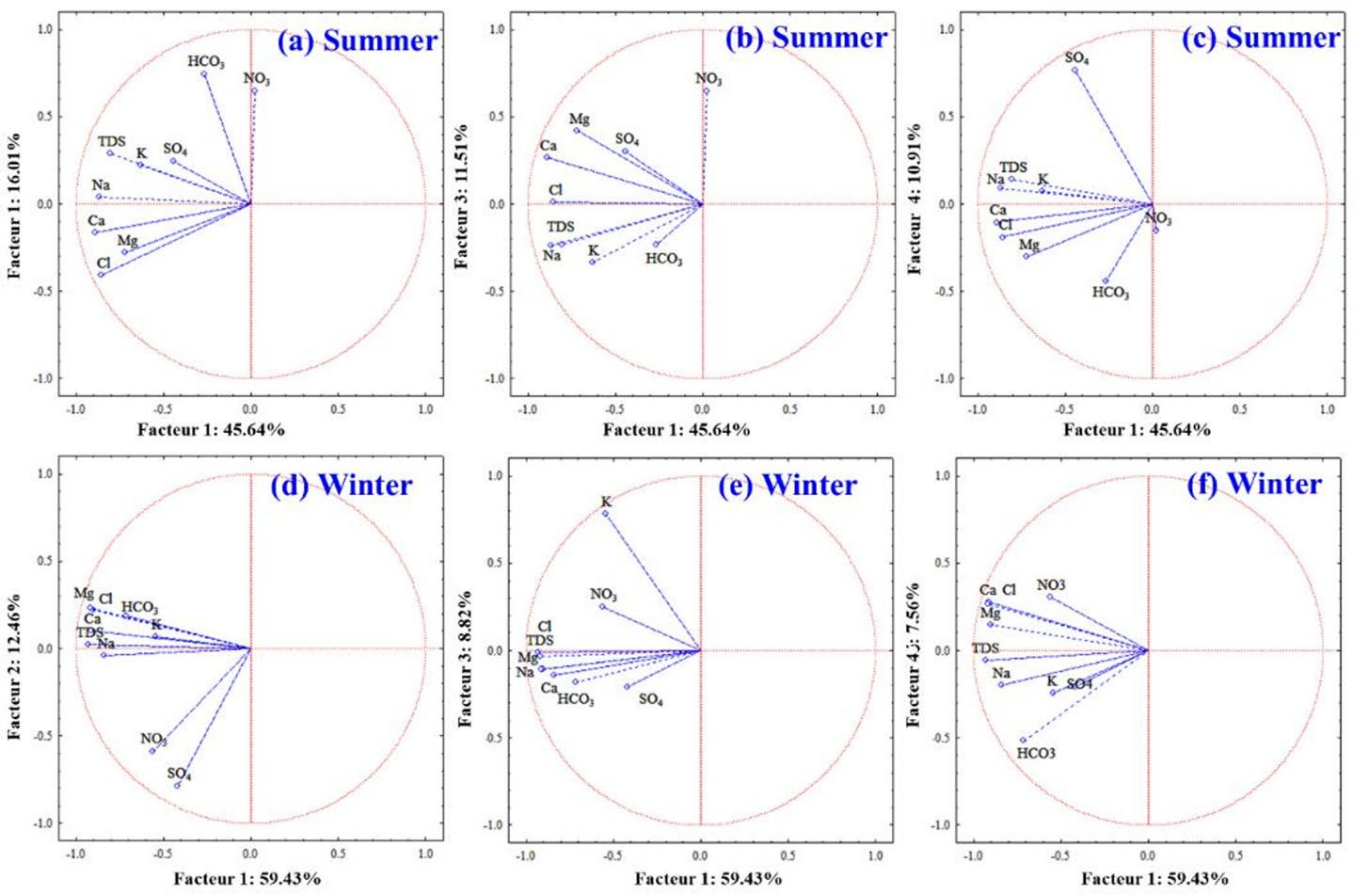
Visualization of the three factors or components extracted according to eigenvalue.

The following tables (Table 5for summer and Table 6for winter) display the correlation coefficients resulting from a Principal Component Analysis (PCA). These coefficients quantify the strength and direction of the linear relationship between each measured hydrochemical parameter (TDS, Mg, Ca, K, Na, Cl, SO₄, HCO₃, NO₃) and the four principal components (Factors 1, 2, 3, and 4) identified by the PCA for both seasonal datasets (visualized in Fig. 7).

Summer PCA reveals that Factor 1 (Horizontal Axis, 45.64% variance) represents overall mineralization, with negative loadings on TDS and major ions, likely exacerbated by evaporation concentrating salts (Fig. 7a-c; Table 5). Factor 2 (Vertical Axis in Figs. 7d and 16.01% variance) is characterized by strong positive correlations with bicarbonate and nitrate, suggesting inputs from organic matter decomposition or surface runoff carrying these constituents. Factor 3 (Vertical Axis in Fig. 7b and 11.51% variance) shows a positive association between magnesium and nitrate, potentially indicating specific mineral dissolution or anthropogenic sources contributing both. Finally, Factor 4 (Vertical Axis in Fig. 7c and 10.01% variance) is strongly positively correlated with sulfate, pointing towards industrial discharge or the dissolution of sulfate-bearing geological formations as significant sources during the summer.

In winter, PCA identified four key factors explaining the variability in Skikda groundwater quality (Fig. 7d-f; Table 6). Factor 1 (Horizontal Axis, 59.43% variance) represents overall mineralization, with strong negative correlations to major ions (TDS, Mg, Ca, Na, Cl), suggesting dilution effects. Factor 2 (Vertical Axis in Figs. 7d and 12.46% variance) is associated with lower sulfate and nitrate levels, potentially indicating limited anthropogenic or geological contributions of these ions during winter. Factor 3 (Vertical Axis in Figs. 7e and 8.82% variance) highlights elevated potassium and nitrate concentrations, possibly pointing to localized sources like fertilizer leaching or sewage. Factor 4 (Vertical Axis in Fig. 7f, 7.56% variance) shows moderate positive correlations with calcium, chloride, and nitrate, hinting at influences from domestic/agricultural activities or specific water-rock interactions releasing these constituents95.

PCA results across both seasons highlight Factor 1 (mineralization/salinity) as the main driver of groundwater variability in the Skikda region (Fig. 7; Tables 5 and 6)). In these studies, specific variables—such as nitrate, sulfate, and potassium—were linked to seasonal agricultural practices, industrial discharges, or hydrological changes, highlighting the effectiveness of multivariate approaches in identifying the primary drivers of water quality variability For example, the sulfate–nitrate coupling in winter (Factor 2) suggests potential agricultural or industrial influence during periods of higher recharge, while the bicarbonate–nitrate pattern in summer (Factor 2) points to enhanced biochemical activity or fertilizer leaching during dry conditions. The dominance of potassium in winter (Factor 3) and sulfate in summer (Factor 4) further emphasizes seasonal controls on water quality, influenced by both natural (e.g., evaporation, geology) and human factors (e.g., agriculture, wastewater).

**Table 5:** The + ve and - ve correlation values extracted for each factor (summer).

	Factor 1	Factor 2	Factor 3	Factor 4
TDS	**-0.805**	0.288	-0.235	0.144
Mg	**-0.717**	-0.280	0.421	-0.300
Ca	**-0.892**	-0.166	0.269	-0.104
K	**-0.630**	0.221	-0.336	0.077
Na	**-0.869**	0.040	-0.238	0.091
Cl	**-0.853**	-0.410	0.009	-0.189
SO_4_	-0.444	0.245	0.302	**0.768**
HCO_3_	-0.266	**0.746**	-0.233	-0.444
NO_3_	0.023	**0.646**	**0.646**	-0.151
Eigenvalue	4.108	1.441	1.036	0.982
% Total	45.643	16.013	11.511	10.909
Cumulative Eigenvalue	4.108	5.549	6.585	7.567
Cumulative %	45.643	61.656	73.167	84.076

Significant values are in [bold].

**Table 6:** The + ve and - ve correlation values extracted for each factor (winter).

	Factor 1	Factor 2	Factor 3	Factor 4
TDS	**-0.931**	0.022	-0.009	-0.056
Mg	**-0.899**	0.220	-0.107	0.150
Ca	**-0.915**	0.102	-0.107	0.277
K	-0.544	0.067	**0.781**	-0.242
Na	**-0.838**	-0.038	-0.139	-0.200
Cl	**-0.916**	0.231	-0.033	0.268
SO_4_	-0.419	**-0.788**	-0.212	-0.209
HCO_3_	**-0.711**	0.187	-0.180	**-0.518**
NO_3_	-0.562	**-0.589**	0.252	0.308
Eigenvalue	5.348	1.121	0.794	0.681
% Total	59.426	12.461	8.822	7.563
Cumulative Eigenvalue	5.348	6.470	7.264	7.945
Cumulative %	59.426	71.887	80.709	88.272

Significant values are in [bold].

### Irrigation index approach

**Fig. 8:**
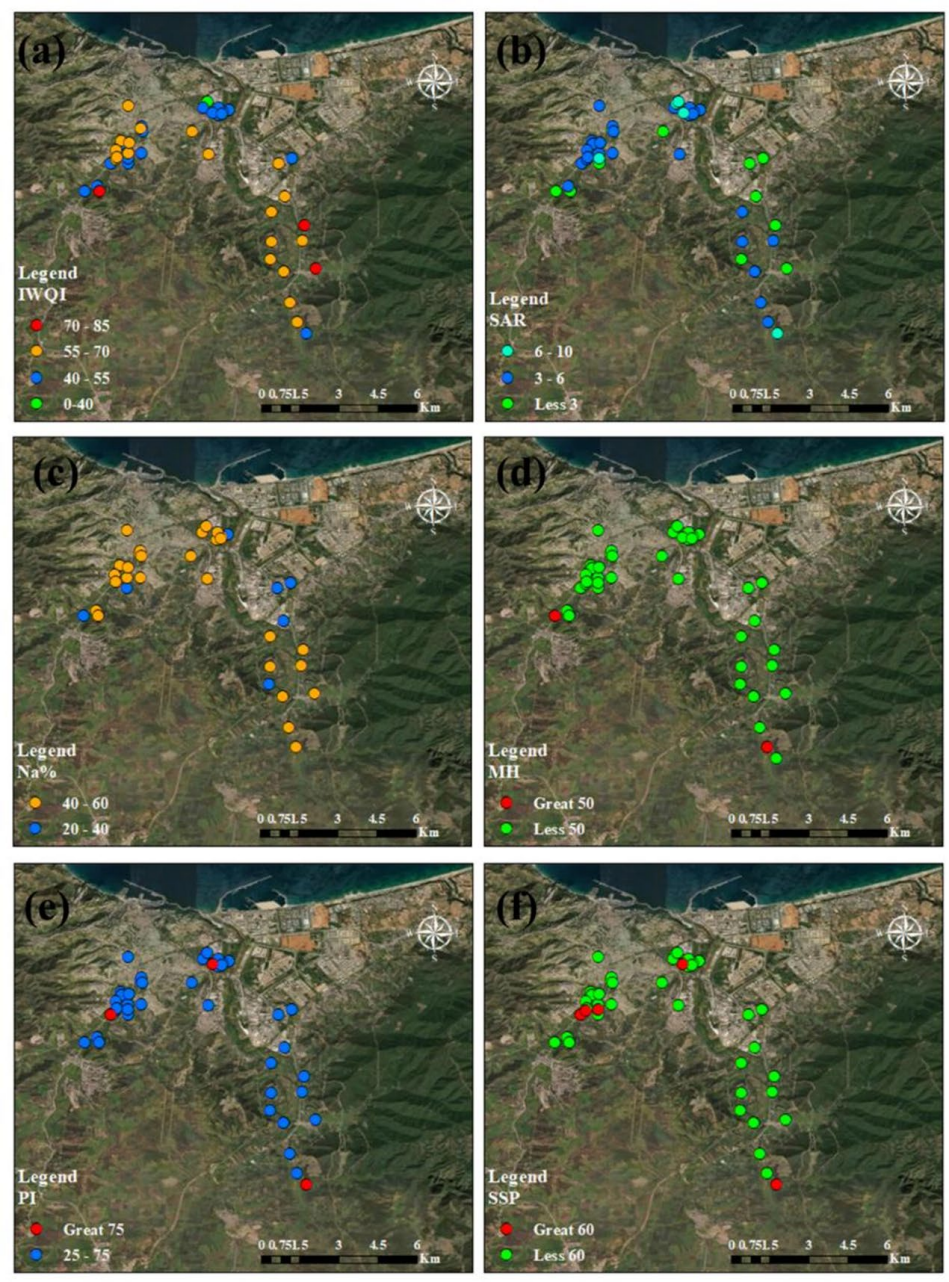
Application of GIS interpolation to detect the spatial classes of different indices and contaminated locations for summer period. Map created using ArcGIS Pro 2.8.8 Software (Esri; https://www.esri.com/arcgis/about-arcgis).

**Fig. 9:**
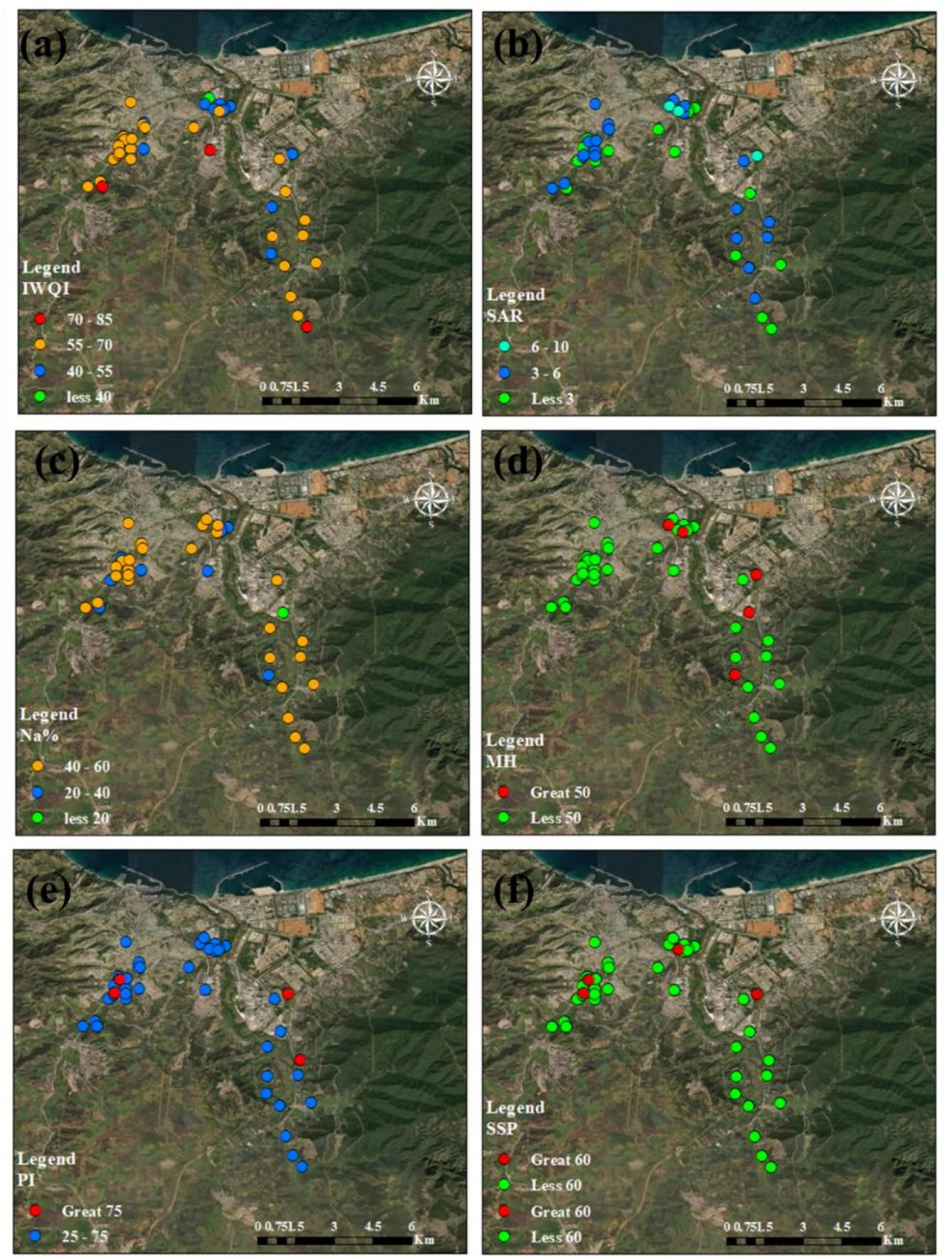
Application of GIS interpolation to detect the spatial classes of different indices and contaminated locations for winter period. Map created using ArcGIS Pro 2.8.8 Software (Esri; https://www.esri.com/arcgis/about-arcgis).

The study aims to evaluate the quality of 44 groundwater samples for irrigation purposes using multiple indices: Irrigation Water Quality Index (IWQI), Sodium Adsorption Ratio (SAR), and Sodium Percentage (Na %), Magnesium Hazard (MH), Permeability Index (PI), and Soluble Sodium Percentage (SSP). These indices assess the suitability of groundwater for irrigation based on chemical composition, focusing on sodium, magnesium, and salinity-related parameters (Tables 7 and 8; Figs. 8 and 9).

#### Irrigation water quality index (IWQI)

IWQI integrates multiple hydrochemical parameters (e.g., electrical conductivity, sodium, chloride, and bicarbonate) to provide a composite score reflecting overall irrigation water quality^71,108^. Higher IWQI values indicate better suitability for irrigation, with specific ranges defining restriction levels. As shown in (Figs. 8a and 9a), the geospatial analysis points out regions with water quality that could affect soil and crop health negatively. In summer, IWQI ranged from 30.36 to 74.00 (mean: 54.56, SD: 9.89). Samples were distributed as 43.2% of water samples in the area has moderate restriction from the northeastern of the study area, 45.5% of water samples in the area has high restriction from the south of the study area, 6.8% low restriction from the northeastern in the southeastern of the study area, and 4.5% severe restriction. In winter, IWQI ranges from 26.88 to 71.32 (mean: 56.70, SD: 10.44). Most samples (59.2%) fall in the moderate restriction category from the south of the study area, 29.5% in high restriction from the northeastern, 6.8% in low restriction, and 4.5% in severe restriction from the southeaster of study area. The IWQI results indicate that the groundwater is generally of moderate to high restriction for irrigation, with no samples achieving “no restriction” (85–100). The slightly lower mean IWQI in summer (54.56 vs. 56.70 in winter) suggests a marginal decline in overall water quality, possibly due to increased evaporation or concentration of solutes during warmer months. The standard deviation (10.44 in winter, 9.89 in summer) reflects moderate variability, indicating spatial heterogeneity in groundwater quality. The dominance of moderate and high restriction categories suggests that most samples are marginally suitable for irrigation, requiring careful management to avoid soil degradation, especially for salt-sensitive crops^46^.

#### Sodium adsorption ratio (SAR)

SAR quantifies the relative proportion of sodium to calcium and magnesium ions, indicating the potential for sodium-induced soil sodicity, which can reduce soil permeability^71,109^. Lower SAR values indicate better water quality for irrigation. In summer, SAR ranged from 1.60 to 7.10 (mean: 4.10, SD: 1.38). All samples (100%) were also excellent (Table 7; Fig. 8b). In winter, SAR ranged from 1.13 to 6.96 (mean: 3.60, SD: 1.24). All samples (100%) were classified as excellent (Table 8; Fig. 9b). The SAR values are consistently low, indicating excellent irrigation water quality with minimal risk of sodium accumulation in soils. The slightly higher mean SAR in summer (4.10 vs. 3.60 in winter) may reflect seasonal concentration effects, but the values remain well below the threshold for good quality. The low standard deviations (1.24 in winter, 1.38 in summer) suggest uniform sodium dynamics across the sampled sites. These results indicate that sodium-related sodicity is not a concern for these groundwater samples, making them highly suitable for irrigation in terms of SAR as shown in (Figs. 8b and 9b). These results showed that irrigated water had no impact on crop output or soil infiltration^45^.

#### Sodium percentage (Na %)

Na% measures the proportion of sodium relative to total cations, influencing soil structure and permeability. High Na% can lead to soil dispersion and reduced water infiltration^71^. In summer, Na% ranged from 28.94 to 69.90 (mean: 49.92, SD: 9.39). Most samples (68.2%) of water samples in the area were “permissible,” 15.9% were good in the geographic centre of the region (Table 7; Fig. 8c), 15.9% were unsuitable, and none are excellent. In winter, Na% ranged from 19.19 to 66.89 (mean: 46.28, SD: 10.36). Most samples (68.2%) of water samples in the area were permissible, 20.4% were good, 9.1% were unsuitable, and 2.3% were excellent from the northeastern of the study areas shown in (Table 8; Fig. 9c). The Na% results indicate that most groundwater samples are within the permissible range for irrigation, though a notable fraction (15.9% in summer, 9.1% in winter) are unsuitable due to high sodium content. The higher mean Na% in summer (49.92 vs. 46.28 in winter) suggests a seasonal increase in sodium dominance, potentially due to evaporative concentration or leaching. The standard deviation (10.36 in winter, 9.39 in summer) indicates moderate variability, reflecting differences in local hydrogeochemical conditions. While the majority of samples are suitable, the presence of unsuitable samples highlights the need for site-specific monitoring to prevent sodium-induced soil degradation.

#### Magnesium hazard (MH)

MH assesses the risk of magnesium dominance over calcium, which can adversely affect soil structure and hydraulic conductivity. Managing magnesium levels is therefore crucial for maintaining soil health and ensuring optimal agricultural productivity^71,110^. MH values < 50 are considered safe for irrigation. In summer, MH ranged from 30.12 to 60.82 (mean: 37.69, SD: 5.78). Most samples (95.5%) were safe, with 4.5% unsafe from extreme south (Table 7; Fig. 8d). In winter, MH ranged from 22.44 to 61.71 (mean: 45.31, SD: 6.76). Most samples (88.6%) of the samples water were safe, while 11.4% were unsafe from the northeastern of the study area as shown in (Table 8; Fig. 9d). The MH results suggest that the groundwater is predominantly safe for irrigation, with a lower proportion of unsafe samples in summer (4.5% vs. 11.4% in winter). The lower mean MH in summer (37.69 vs. 45.31 in winter) indicates a seasonal improvement, possibly due to dilution or changes in ion ratios. The standard deviations (6.76 in winter, 5.78 in summer) reflect low to moderate variability, suggesting relatively consistent magnesium dynamics. The small fraction of unsafe samples indicates a minor risk of magnesium-induced soil issues, but overall, MH is not a significant limiting factor for irrigation^45,111^.

#### Permeability index (PI)

PI evaluates the suitability of water for irrigation based on its effect on soil permeability, considering sodium, calcium, magnesium, and bicarbonate concentrations. Higher PI values indicate better suitability. Class I (PI > 75%) is considered as suitable for irrigation and class II (PI = 25–75%) is considered as reasonably suitable, Class III (PI < 25%) is unacceptable^71,110,112^. In summer, PI ranged from 38.92 to 83.39 (mean: 61.66, SD: 9.52). Most samples (93.2%) fell into suitable – Class II, 6.8% fell into suitable – Class I, and none are Unsuitable – Class III as shown in (Table 7; Fig. 8e). In winter, PI ranged from 33.06 to 81.40 (mean: 58.82, SD: 11.76). Most samples (90.9%) fell into reasonably suitable – Class II, 9.1% fell into suitable – Class I, and none were Unsuitable – Class III as shown in (Table 8; Fig. 9e). The PI results indicate that the groundwater is highly suitable for irrigation, with nearly all samples falling in the “Good” categories. The slightly higher mean PI in summer (61.66 vs. 58.82 in winter) suggests improved permeability characteristics, possibly due to changes in bicarbonate or cation ratios. The standard deviations (9.52 in summer, 11.76 in winter) reflect moderate variability, indicating some spatial differences in water chemistry. The absence of unsuitable samples underscores the favourable permeability characteristics of these groundwater samples, making them broadly suitable for sustaining soil hydraulic properties. So, these results confirm that the water quality of the drains is appropriate for irrigation purposes^111^.

#### Soluble sodium percentage (SSP)

SSP measures the proportion of soluble sodium relative to total cations, similar to Na%, and is used to assess sodium-related risks to soil structure. SSP values < 60 is considered safe^71,73^.

In summer, SSP ranged from 28.83 to 69.88 (mean: 49.32, SD: 9.40). Most samples (88.6%) fell into safe, with 11.4% of the samples water are unsafe which found in the southeastern of study area (Table 7; Fig. 8f). In winter, SSP ranged from 19.10 to 66.80 (mean: 45.82, SD: 10.45). Most samples (93.2%) of the samples water fell into safe, with 6.8% fell into unsafe which found in the centre of the study area ((Table 8; Fig. 9f). The SSP results mirror Na% trends, with most samples being safe for irrigation. The higher mean SSP in summer (49.32 vs. 45.82 in winter) and increased proportion of unsafe samples (11.4% vs. 6.8%) suggest a seasonal increase in sodium dominance, consistent with evaporative concentration. The standard deviations (10.45 in winter, 9.40 in summer) indicate moderate variability, reflecting site-specific geochemical differences. While the majority of samples are safe, the presence of unsafe samples in both seasons highlights the need for careful irrigation management to mitigate sodium accumulation in soils.

**Table 7:** Statistical analyses and classification of the different irrigation water quality indices (IWQIs) in Summer.

Irrigation water quality indices (IWQIs)	Sample range				Range	Water category	Number of samples (%)
	Min.	Max.	Mean	SD			
Irrigation water quality index (IWQI)	30.36	74	54.56	9.89	85–100	No restriction	4.50%
					70–85	Low restriction	45.50%
					55–70	Moderate restriction	43.20%
					40–55	High restriction	6.80%
					0–40	Serve restriction	0%
Sodium adsorption ratio (SAR)	1.6	7.1	4.1	1.38	< 10	Excellent	100%
					10–18	Good	0%
					18–26	Doubtful or Fairly Poor	0%
					> 26	Unsuitable	0%
Sodium percentage (Na %)	28.94	69.9	49.92	9.39	< 20	Excellent	0%
					20–40	Good	15.90%
					40–60	Permissible	68.20%
					> 60	Unsuitable	15.90%
Magnesium Hazard (MH)	30.12	60.82	37.69	5.78	< 50	Safe	95.50%
					> 50	Unsafe	4.50%
Permeability Index (PI)	38.92	83.39	61.66	9.52	> 75	Good – Class I	6.80%
					25–75	Good – Class II	93.2
					< 25	Unsuitable – Class III	0%
Soluble sodium percentage (SSP)	28.83	69.88	49.32	9.4	< 60	Safe	88.60%
					> 60	Unsafe	11.40%

**Table 8:** Statistical analyses and classification of the different irrigation water quality indices (IWQIs) in winter.

Irrigation water quality indices (IWQIs)	Sample range				Range	Water category	Number of samples (%)
	Min.	Max.	Mean	SD			
Irrigation water quality index (IWQI)	26.88	71.32	56.7	10.44	85–100	No restriction	4.50%
					70–85	Low restriction	29.50%
					55–70	Moderate restriction	59.20%
					40–55	High restriction	6.80%
					0–40	Serve restriction	0%
Sodium adsorption ratio (SAR)	1.13	6.96	3.6	1.24	< 10	Excellent	100%
					10–18	Good	0%
					18–26	Doubtful or Fairly Poor	0%
					> 26	Unsuitable	0%
Sodium percent (Na %)	19.19	66.89	46.28	10.36	< 20	Excellent	2.30%
					20–40	Good	20.40%
					40–60	Permissible	68.20%
					> 60	Unsuitable	9.10%
Magnesium hazard (MH)	22.44	61.71	45.31	6.76	< 50	Safe	88.60%
					> 50	Unsafe	11.40%
Permeability index (PI)	33.06	81.4	58.82	11.76	> 75	Good – Class I	9.10%
					25–75	Good – Class II	90.90%
					< 25	Unsuitable – Class III	0%
Soluble sodium percentage (SSP)	19.1	66.8	45.82	10.45	< 60	Safe	93.20%
					> 60	Unsafe	6.80%

### The efficiency of machine learning algorithms in predicting WQIs

This study employed MLAs such as RF, ABR, and XGBR integrated with SHAP analysis to enhance WQI monitoring. Eleven physicochemical parameters (pH, EC, TDS, Mg, Ca, K, Na, Cl, SO_4_, HCO_3_, NO_3_) were analyzed using SHAP to identify the top five most predictive features as model inputs. Model performance was evaluated using RMSE, MAE, NSE, WI, and R^2^.

Applied to summer data, SHAP analysis identified the five most significant features influencing MLA predictions (Table 9). Three MLAs including, RF, ABR, and XGBR were assessed for their ability to predict six WQIs: IWQI, SAR, Na%, MR, PI, and SSP (see Table 10). The comparative analysis revealed that XGBR consistently underperformed across all indices, for instance in IWQI validation (RMSE = 4.95, MAE = 3.93, R^2^ = 0.74), indicating challenges in modeling intricate nonlinear relationships. On the other hand, RF showcased superior accuracy across all WQIs, with strong validation outcomes: such as IWQI (RMSE = 2.41, MAE = 1.83, NSE = 0.94, WI = 0.88, R^2^ = 0.94), SAR (RMSE = 0.37, MAE = 0.28, NSE = 0.92, WI = 0.86, R^2^ = 0.92), Na% (RMSE = 3.36, MAE = 2.35, NSE = 0.87, WI = 0.81, R^2^ = 087), MR (RMSE = 2.99, MAE = 1.90, NSE = 0.73, WI = 0.69, R^2^ = 0.73), PI (RMSE = 2.99, MAE = 2.20, NSE = 0.90, WI = 0.82, R^2^ = 0.90), and SSP (RMSE = 2.95, MAE = 2.17, NSE = 0.90, WI = 0.83, R^2^ = 0.90), highlighting its precision in predicting these WQIs.

While ABR displayed varying predictive efficacy, it performed well in IWQI validation (RMSE = 3.53, MAE = 2.54, NSE = 0.87, WI = 0.82, R^2^ = 0.87), SAR validation (RMSE = 5.04, MAE = 3.59, NSE = 0.71, WI = 0.70, R^2^ = 0.71), PI validation (RMSE = 4.19, MAE = 2.94, NSE = 0.80, WI = 0.75, R^2^ = 0.80), and SSP validation (RMSE = 3.00, MAE = 2.29, NSE = 0.90, WI = 0.82, R^2^ = 0.90). However, it struggled with MR prediction, as indicated by MR validation results (RMSE = 5.26, MAE = 3.45, NSE = 0.67, WI = 0.62, R^2^ = 0.67), pointing towards its limitations in capturing complex nonlinear relationships.

**Table 9:** SHAP analysis for summer data feature selection.

WQIs	Models	pH	EC	TDS	Mg	Ca	K	Na	Cl	SO_4_	HCO_3_	NO_3_
IWQI	RF	0.27	**0.97**	**0.41**	0.18	0.14	0.13	**4.11**	**2.7**	0.31	**0.52**	0.15
	ABR	0.03	**0.39**	**0.72**	**1.1**	0.25	0.02	**5.1**	**1.53**	0.10	0.04	0.05
	XGBR	0.02	**0.44**	**0.61**	**0.68**	0.03	0.01	**2.86**	**1.31**	0.0	0.0	0.0
SAR	RF	0.03	0.03	**0.06**	**0.05**	**0.07**	**0.06**	**1.1**	0.02	0.015	0.015	0.02
	ABR	**0.01**	0.04	0.0	0.01	**0.11**	**0.03**	**0.96**	0.0	0.0	0.0	**0.03**
	XGBR	**0.02**	0.0	0.0	**0.01**	0.0	0.0	**0.64**	0.0	**0.04**	0.0	**0.01**
Na%	RF	0.14	**0.29**	0.12	**1.84**	**1.77**	**0.36**	**5.81**	0.06	0.07	0.13	0.19
	ABR	0.06	0.03	**0.45**	**1.53**	**2.26**	0.03	**5.28**	0.01	0.10	0.01	**0.22**
	XGBR	**0.06**	0.05	**0.09**	**0.85**	**1.56**	0.04	**3.28**	0.0	0.0	0.02	0.02
MR	RF	**0.40**	**0.32**	0.05	**1.56**	**0.85**	0.31	0.24	0.16	**0.33**	0.22	0.05
	ABR	**0.10**	0.08	0.02	**1.13**	**1.08**	0.0	**0.15**	0.0	**0.25**	0.02	0.07
	XGBR	0.04	**0.10**	**0.05**	**1.31**	**0.47**	**0.43**	0.0	0.0	0.0	0.0	0.0
PI	RF	0.14	0.26	**0.32**	**2.08**	**3.39**	0.15	**3.47**	0.13	0.24	**0.39**	0.24
	ABR	0.01	0.13	**0.55**	**0.96**	**5.57**	0.01	**3.32**	0.03	0.09	**0.28**	0.12
	XGBR	0.07	**0.25**	**0.96**	**0.46**	**3.60**	0.0	**1.99**	0.0	0.0	0.16	0.0
SSP	RF	0.22	0.21	0.07	**1.25**	**1.93**	**0.46**	**5.37**	0.16	0.06	0.06	**0.50**
	ABR	**0.23**	0.0	**0.15**	**1.86**	**1.71**	0.05	**5.53**	0.0	0.09	0.0	0.13
	XGBR	0.03	0.05	**0.14**	**1.05**	**1.53**	**0.08**	**3.33**	0.05	0.04	0.04	0.01

Significant values are in [bold].

**Table 10:** Performance of mlas in predicting WQIs based on summer data.

WQIs	Models	Optimal features	Calibration					Validation				
			RMSE	MAE	NSE	WI	R^2^	RMSE	MAE	NSE	WI	R^2^
IWQI	RF	EC, TDS, Na, Cl, HCO_3_	2.24	1.59	0.95	0.89	0.95	2.41	1.83	0.94	0.88	0.94
	ABR	pH, EC, TDS, Na, Cl	2.20	1.58	0.95	0.90	0.95	3.53	2.54	0.87	0.82	0.87
	XGBR	EC, TDS, Mg, Na, Cl	4.64	3.60	0.78	0.71	0.78	4.95	3.93	0.74	0.68	0.74
SAR	RF	TDS, Mg, Ca, K, Na	0.24	0.17	0.97	0.92	0.97	0.37	0.28	0.92	0.86	0.92
	ABR	pH, Ca, K, Na, NO_3_	0.40	0.29	0.92	0.86	0.92	0.50	0.36	0.87	0.83	0.87
	XGBR	pH, Mg, Na, SO_4_, NO_3_	0.73	0.54	0.72	0.69	0.72	0.78	0.59	0.67	0.65	0.67
Na%	RF	EC, Mg, Ca, K, Na	2.63	1.75	0.92	0.87	0.92	3.36	2.35	0.87	0.81	0.87
	ABR	TDS, Mg, Ca, Na, NO_3_	3.81	2.64	0.83	0.79	0.83	5.04	3.59	0.71	0.70	0.71
	XGBR	pH, TDS, Mg, Ca, Na	4.65	3.54	0.75	0.68	0.75	5.15	3.85	0.69	0.64	0.69
MR	RF	pH, EC, Mg, Ca, SO_4_	2.51	1.47	0.81	0.78	0.81	2.99	1.90	0.73	0.69	0.73
	ABR	pH, Mg, Ca, Na, SO_4_	2.62	2.06	0.79	0.66	0.79	5.26	3.45	0.67	0.62	0.67
	XGBR	EC, TDS, Mg, Ca, K	3.17	2.10	0.69	0.63	0.70	3.63	2.30	0.60	0.58	0.60
PI	RF	TDS, Mg, Ca, Na, HCO_3_	2.30	1.69	0.94	0.87	0.94	2.99	2.20	0.90	0.82	0.90
	ABR	TDS, Mg, Ca, Na, HCO_3_	2.86	2.10	0.91	0.83	0.91	4.19	2.94	0.80	0.75	0.80
	XGBR	EC, TDS, Mg, Ca, Na	4.94	3.69	0.72	0.65	0.72	5.11	3.91	0.71	0.62	0.71
SSP	RF	Mg, Ca, K, Na, NO_3_	2.33	1.74	0.94	0.87	0.94	2.95	2.17	0.90	0.83	0.90
	ABR	pH, TDS, Mg, Ca, Na	2.71	2.07	0.92	0.84	0.92	3.00	2.29	0.90	0.82	0.90
	XGBR	TDS, Mg, Ca, K, Na	4.59	3.34	0.76	0.69	0.76	4.81	3.62	0.74	0.66	0.74

Applied to winter data, SHAP analysis identified the five most significant features influencing MLA predictions (Table 11). Three MLAs including, RF, ABR, and XGBR were assessed for predicting six WQIs: IWQI, SAR, Na%, MR, PI, and SSP (refer to Table 12). The analysis highlighted that XGBR consistently underperformed across all indices, such as IWQI (RMSE = 5.45, MAE = 4.14, NSE = 0.72, WI = 0.67, R^2^ = 0.72), indicating challenges in modeling complex nonlinear relationships. On the contrary, RF exhibited superior accuracy for all WQIs with strong validation results: for instance, IWQI (RMSE = 3.96, MAE = 2.82, NSE = 0.85, WI = 0.81, R^2^ = 0.85), SAR (RMSE = 0.37, MAE = 0.27, NSE = 0.91, WI = 0.85, R^2^ = 0.91), Na% (RMSE = 4.42, MAE = 3.15, NSE = 0.81, WI = 0.80, R^2^ = 0.81), MR (RMSE = 2.62, MAE = 1.92, NSE = 0.85, WI = 0.78, R^2^ = 0.85), PI (RMSE = 3.89, MAE = 2.79, NSE = 0.89, WI = 0.85, R^2^ = 0.89), and SSP (RMSE = 3.49, MAE = 2.58, NSE = 0.89, WI = 0.83, R^2^ = 0.89), showcasing its proficiency in precise prediction of these WQIs.

While ABR demonstrated varying efficacy in prediction, it performed well in IWQI validation (RMSE = 2.95, MAE = 1.66, NSE = 0.92, WI = 0.90, R^2^ = 0.92), SAR validation (RMSE = 0.40, MAE = 0.31, NSE = 0.90, WI = 0.82, R²=0.90), MR validation (RMSE = 2.77, MAE = 1.99, NSE = 0.83, WI = 0.76, R^2^ = 0.83), PI validation (RMSE = 4.52, MAE = 3.20, NSE = 0.85, WI = 0.81, R²=0.85), and SSP validation (RMSE = 4.23, MAE = 3.08, NSE = 0.83, WI = 0.78, R^2^ = 0.83). However, it faced challenges in Na% prediction, as seen in the Na% validation results (RMSE = 5.03, MAE = 3.70, NSE = 0.76, WI = 0.75, R^2^ = 0.76), indicating limitations in capturing complex nonlinear relationships.

**Table 11:** SHAP analysis for winter data feature selection.

WQIs	Models	pH	EC	TDS	Mg	Ca	K	Na	Cl	SO_4_	HCO_3_	NO_3_
IWQI	RF	**0.29**	**0.85**	0.51	**0.90**	0.28	0.17	**5.36**	0.17	0.47	**1.17**	0.27
	ABR	**0.40**	0.27	0.32	**1.08**	0.32	0.10	**4.12**	**2.33**	0.16	**0.68**	0.13
	XGBR	0.15	**0.26**	0.0	**0.76**	0.0	**0.40**	**2.65**	**1.45**	0.0	0.09	0.0
SAR	RF	0.005	0.03	0.005	**0.09**	**0.09**	**0.04**	**0.83**	**0.05**	0.02	0.03	0.03
	ABR	0.0	0.0	0.0	**0.33**	0.0	0.0	**0.89**	0.0	**0.11**	**0.01**	**0.03**
	XGBR	0.005	0.02	0.02	**0.06**	**0.09**	0.02	**0.51**	**0.03**	0.005	0.01	**0.03**
Na%	RF	0.45	0.52	0.46	0.33	**7.47**	0.23	**2.05**	0.42	**0.82**	**1.26**	**0.55**
	ABR	0.18	0.12	**0.44**	**0.84**	**5.91**	**0.36**	**2.72**	0.33	0.04	0.21	0.05
	XGBR	0.18	0.12	**0.44**	**0.84**	**5.91**	**0.36**	**2.72**	0.33	0.03	0.21	0.06
MR	RF	0.50	0.04	0.35	**4.75**	**2.71**	0.05	**0.68**	**0.55**	0.13	**0.65**	0.36
	ABR	0.10	0.21	0.0	**3.66**	**1.91**	**0.35**	**0.80**	**0.34**	0.03	0.0	0.03
	XGBR	0.0	0.0	0.0	**1.78**	0.0	0.0	0.0	**0.01**	**0.01**	**0.01**	**0.01**
PI	RF	0.41	0.13	0.23	**2.37**	**6.72**	**0.43**	**1.43**	0.24	0.10	**1.23**	0.35
	ABR	0.17	0.15	0.35	**2.55**	**7.05**	**0.60**	**1.23**	0.09	0.18	**0.75**	0.12
	XGBR	0.0	0.0	0.0	**1.17**	**4.01**	0.0	**0.78**	0.0	**0.02**	0.0	**0.01**
SSP	RF	0.25	0.15	**0.44**	**1.81**	**4.52**	0.15	**3.32**	0.19	0.26	**0.62**	0.24
	ABR	0.07	0.07	0.14	**0.70**	**5.19**	0.24	**2.78**	0.15	**0.24**	**0.39**	0.14
	XGBR	0.07	0.0	0.05	**0.69**	**3.37**	0.0	**2.19**	0.0	**0.08**	0.04	**0.14**

Significant values are in [bold].

**Table 12:** Performance of mlas in predicting WQIs based on winter data.

WQIs	Models	Optimal features	Calibration					Validation				
			RMSE	MAE	NSE	WI	R^2^	RMSE	MAE	NSE	WI	R^2^
IWQI	RF	pH, EC, Mg, Na, HCO_3_	3.76	2.71	0.87	0.82	0.87	3.96	2.82	0.85	0.81	0.85
	ABR	pH, Mg, Na, Cl, HCO_3_	1.55	1.18	0.98	0.93	0.98	2.95	1.66	0.92	0.90	0.92
	XGBR	EC, Mg, K, Na, Cl	4.96	3.67	0.77	0.72	0.77	5.45	4.14	0.72	0.67	0.72
SAR	RF	Mg, Ca, K, Na, Cl	0.21	0.16	0.97	0.91	0.97	0.37	0.27	0.91	0.85	0.91
	ABR	Mg, Na, SO_4_, HCO_3_, NO_3_	0.34	0.27	0.92	0.85	0.92	0.40	0.31	0.90	0.82	0.90
	XGBR	Mg, Ca, Na, Cl, NO3	0.58	0.43	0.78	0.71	0.78	0.67	0.46	0.70	0.69	0.70
Na%	RF	Ca, Na, SO_4_, HCO_3_, NO_3_	3.94	2.58	0.85	0.84	0.85	4.42	3.15	0.81	0.80	0.81
	ABR	TDS, Mg, Ca, K, Na	4.52	3.36	0.81	0.78	0.81	5.03	3.70	0.76	0.75	0.76
	XGBR	TDS, Mg, Ca, K, Na	5.30	3.99	0.73	0.68	0.73	5.85	4.47	0.67	0.64	0.67
MR	RF	Mg, Ca, Na, Cl, HCO_3_	2.20	1.6	0.89	0.82	0.89	2.62	1.92	0.85	0.78	0.85
	ABR	Mg, Ca, K, Na, Cl	1.94	1.52	0.92	0.83	0.92	2.77	1.99	0.83	0.76	0.83
	XGBR	Mg, Cl, SO_4_, HCO_3_, NO_3_	4.57	3.09	0.69	0.51	0.69	4.89	3.67	0.61	0.58	0.61
PI	RF	Mg, Ca, K, Na, HCO_3_	3.35	2.26	0.92	0.88	0.92	3.89	2.79	0.89	0.85	0.89
	ABR	Mg, Ca, K, Na, HCO_3_	2.85	2.32	0.94	0.87	0.94	4.52	3.20	0.85	0.81	0.85
	XGBR	Mg, Ca, Na, SO_4_, NO_3_	5.45	4.27	0.78	0.71	0.78	6.06	4.67	0.73	0.69	0.73
SSP	RF	TDS, Mg, Ca, Na, HCO_3_	2.68	1.89	0.93	0.88	0.93	3.49	2.58	0.89	0.83	0.89
	ABR	Mg, Ca, Na, SO_4_, HCO_3_	3.36	2.41	0.89	0.84	0.89	4.23	3.08	0.83	0.78	0.83
	XGBR	Mg, Ca, Na, SO_4_, NO_3_	4.97	3.69	0.77	0.71	0.77	5.39	4.07	0.73	0.68	0.73

Our results indicate that RF models generally were more effective in forecasting WQIs than both ABR and XGBR models. To ensure that our model did not learn unduly from the data, LOOCV was used to search for hyper-parameter optimization and SHAP analysis for optimal feature selection. The resulting RF models (Tables 11 and 12) exhibit only small gaps between calibration and validation performance (R^2^ and RMSE), making significant overfitting unlikely. Accurately forecasting water quality trends requires modeling complex, nonlinear interactions among degradation factors. Advanced multivariate regression techniques have emerged as powerful alternatives to conventional methods, offering enhanced predictive accuracy and operational efficiency^113–115^. Studies demonstrate the particular efficacy of MLAs (e.g., RF, ABR, and XGBR) for predicting WQIs^115–117^. Hassan et al. 2021 achieved exceptional accuracy (98.99%) using RF with trace components to predict WQIs, while Khoi et al., 2022 confirmed XGBR’s precision (R^2^ = 0.989, RMSE = 0.107), ABR’s precision (R^2^ = 0.973, RMSE = 0.175), and RF’s precision (R^2^ = 0.986, RMSE = 0.121). Similarly, El Bilali et al., 2021 reported robust performance for RF (R^2^ = 0.92) using physical parameters, while Bui et al., 2020 achieved R^2^ values of 0.93 using RF model with physicochemical parameters. The study demonstrates that MLAs not only simplify WQI computation but also clarify the importance of input factors, making them a valuable tool for water quality assessments. These models offer a reliable, efficient alternative to traditional methods, which often involve complex calculations and sub-index formulas. Given their comprehensive and consistent performance, the study encourages water quality monitoring agencies to adopt MLAs-based approaches for more effective and time-efficient resource management.

## The limitation and future outlook

The study is geographically concentrated on the Skikda aquifer, potentially restricting the direct application of the created models to other places with distinct geological and climatic characteristics without further calibration. The sample size, although enough for the used models, is somewhat limited (44 samples each season), and an expanded dataset might enhance the robustness and accuracy of the machine learning predictions.

In addition, the study focuses on physicochemical factors rather than analyzing particular contaminants in depth, such as toxic heavy metals, which might offer a more comprehensive understanding of anthropogenic pressure. While SHAP analysis improves interpretability, machine learning models remain “black boxes” to some extent, and their predictions are influenced by the quality and breadth of incoming data. Finally, while the study finds seasonal fluctuations, it is based on data from a single year; long-term monitoring is required to understand the implications of climate variability and continuous anthropogenic activities on groundwater quality trends.

## Conclusion

The irrigation suitability of groundwater in Algeria’s Skikda coastal aquifer was analyzed using a combination of hydrogeochemical characteristics, GIS, and machine learning (ML). The study revealed that groundwater is mostly composed of Ca-Mg-SO_4_ and Na-Cl, with chemistry influenced by evaporation, rock-water interactions, and human activity. Despite most samples being moderately to severely limited for irrigation due to salinity hazards, critical indices such as SAR and PI showed good quality with minimal sodicity hazards. The IWQI revealed that most samples have moderate to high restrictions, limiting their suitability for salt-sensitive crops. The SAR and PI indices indicate excellent to good water quality, with minimal risk of sodium-induced sodicity and favorable soil permeability characteristics. The MH and SSP results revealed that most samples are safe, with low risks of magnesium or sodium-related soil degradation. The Na% and SSP showed unsuitable samples for irrigation, particularly in summer, indicating localized sodium risks. Summer samples generally showed a slightly poor quality (higher Na%, SSP, and lower IWQI) compared to winter, likely due to evaporative concentration of solutes. However, MH and PI improve marginally in summer, suggesting some seasonal buffering in magnesium and permeability dynamics.

The groundwater is marginally suitable for irrigation but requires careful management, particularly to mitigate salinity risks. Strategies such as blending with lower-salinity water, selecting salt-tolerant crops, and implementing efficient irrigation practices (e.g., drip irrigation) are recommended. Site-specific monitoring is essential, given the variability in Na%, and SSP, to prevent long-term soil degradation. In summary, while the groundwater is broadly suitable for irrigation based on SAR, PI, MH, and SSP, and moderate IWQI restrictions necessitate proactive management to ensure sustainable agricultural use. Further hydrogeochemical studies and soil monitoring are recommended to refine irrigation strategies and protect soil health.

Among the ML models tested—Random Forest (RF), Extreme Gradient Boosting (XGBR), and Adaptive Boosting (ABR)—the RF model consistently demonstrated superior performance in predicting irrigation water quality indices (IWQIs) for both seasons, proving to be a highly accurate and reliable tool. Comparative analysis reveals indicate that RF models generally were more effective in forecasting WQIs than both ABR and XGBR models. The results found that RF exhibited superior accuracy for all WQIs with strong validation results: IWQI (R^2^ = 0.85 and 0.94), SAR (R^2^ = 0.91 and 0.92), Na% (R^2^ = 0.81 and 0.87), MR (R^2^ = 0.85 and 0.73), PI (R^2^ = 0.89 and 0.90), and SSP (R^2^ = 0.89 and 0.90). These results highlight RF’s capability for accurately predicting WQIs for both summer and winter datasets. Finally, the integration of GIS with the RF model offers a powerful, replicable framework for efficient groundwater quality forecast and gives a clear picture for water quality management and sustainable irrigation.

## Supplementary Information

Below is the link to the electronic supplementary material.

Supplementary Material 1

Supplementary Material 2

Supplementary Material 3

Supplementary Material 4

Supplementary Material 5

Supplementary Material 6

Supplementary Material 7

Supplementary Material 8

Supplementary Material 9

Supplementary Material 10

Supplementary Material 11

Supplementary Material 12

Supplementary Material 13

Supplementary Material 14

Supplementary Material 15

Supplementary Material 16

Supplementary Material 17

Supplementary Material 18

Supplementary Material 19

Supplementary Material 20

## Data Availability

All data are provided as tables and figures in the manuscript and supplementary material.
