# Supplementary figures and images for: Integrated GIS-machine learning approach to irrigation water quality assessment in coastal aquifers
Source: Sci Rep. 2026 Jan 28;16:3894. doi: 10.1038/s41598-025-25461-y (PMC12855904; doi:10.1038/s41598-025-25461-y)

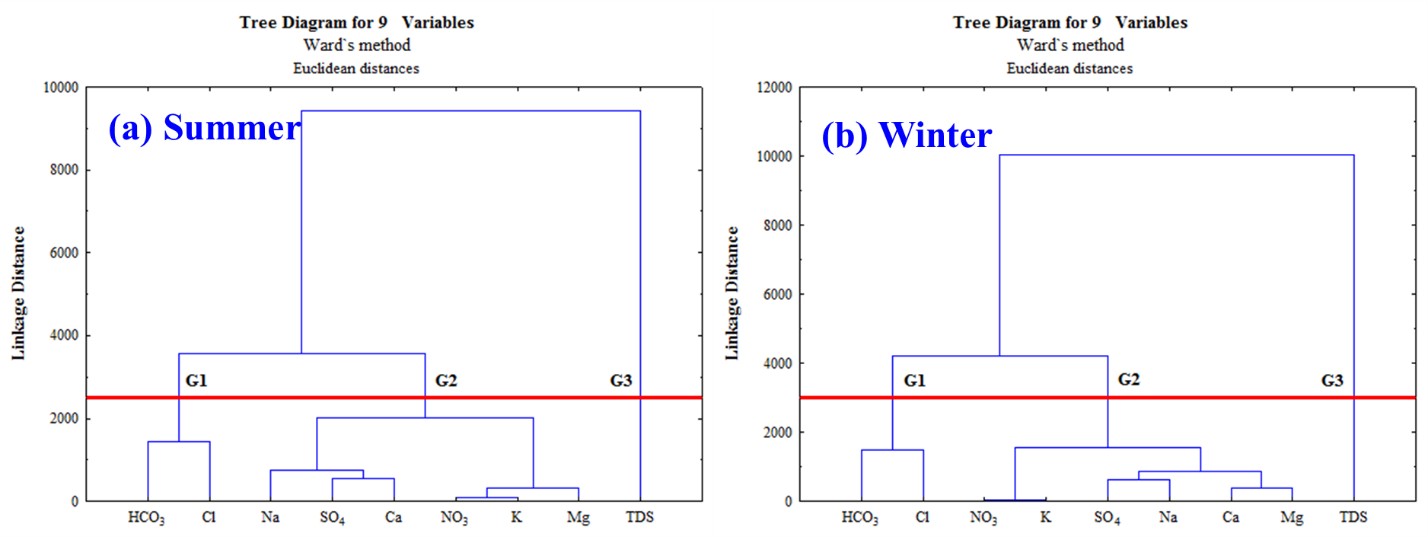

Supplement: Supplementary file 1 — Supplementary Material 1 [file 41598_2025_25461_MOESM1_ESM.jpg]

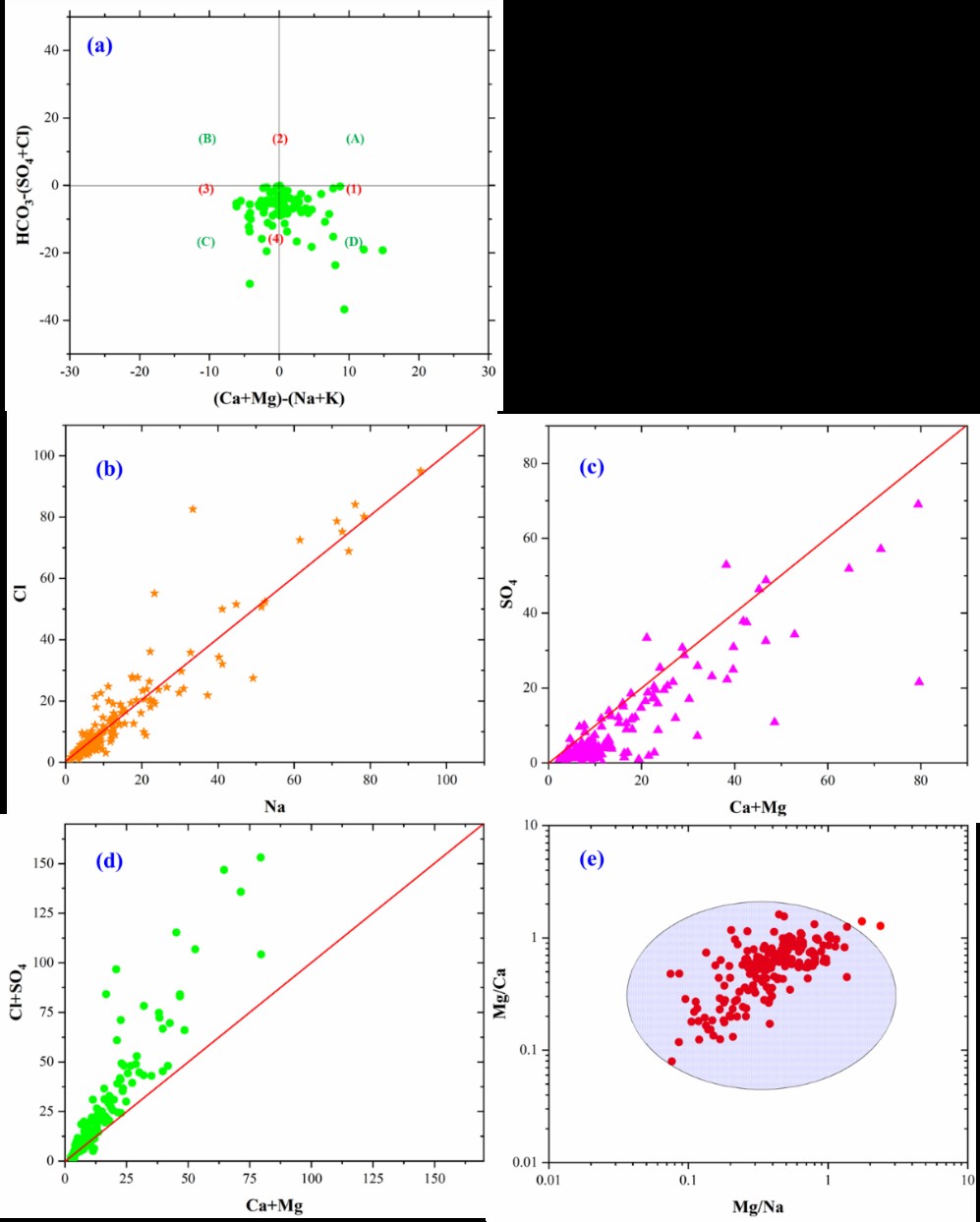

Supplement: Supplementary file 2 — Supplementary Material 2 [file 41598_2025_25461_MOESM2_ESM.jpg]

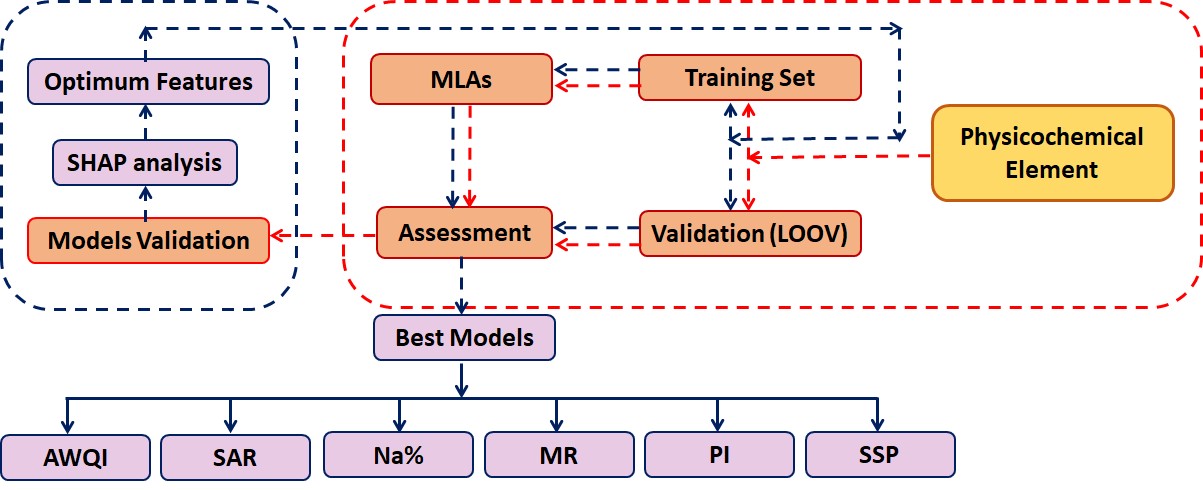

Supplement: Supplementary file 3 — Supplementary Material 3 [file 41598_2025_25461_MOESM3_ESM.jpg]

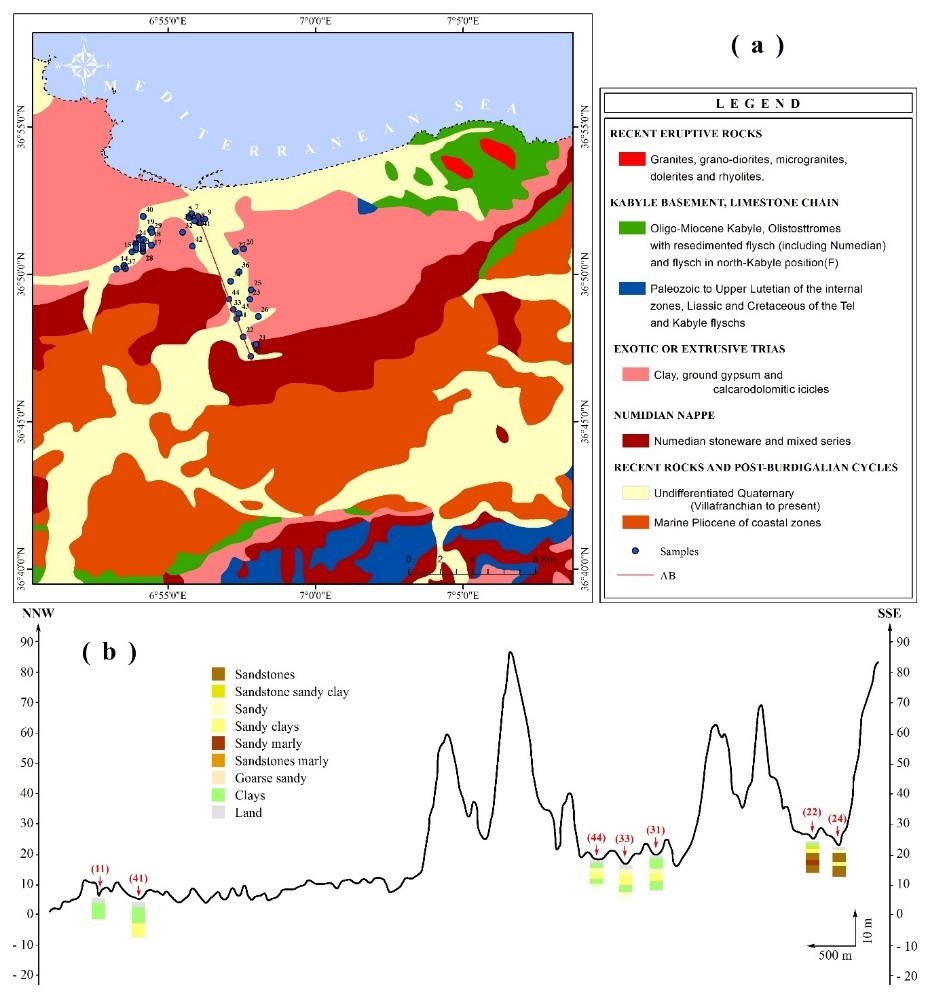

Supplement: Supplementary file 4 — Supplementary Material 4 [file 41598_2025_25461_MOESM4_ESM.jpg]

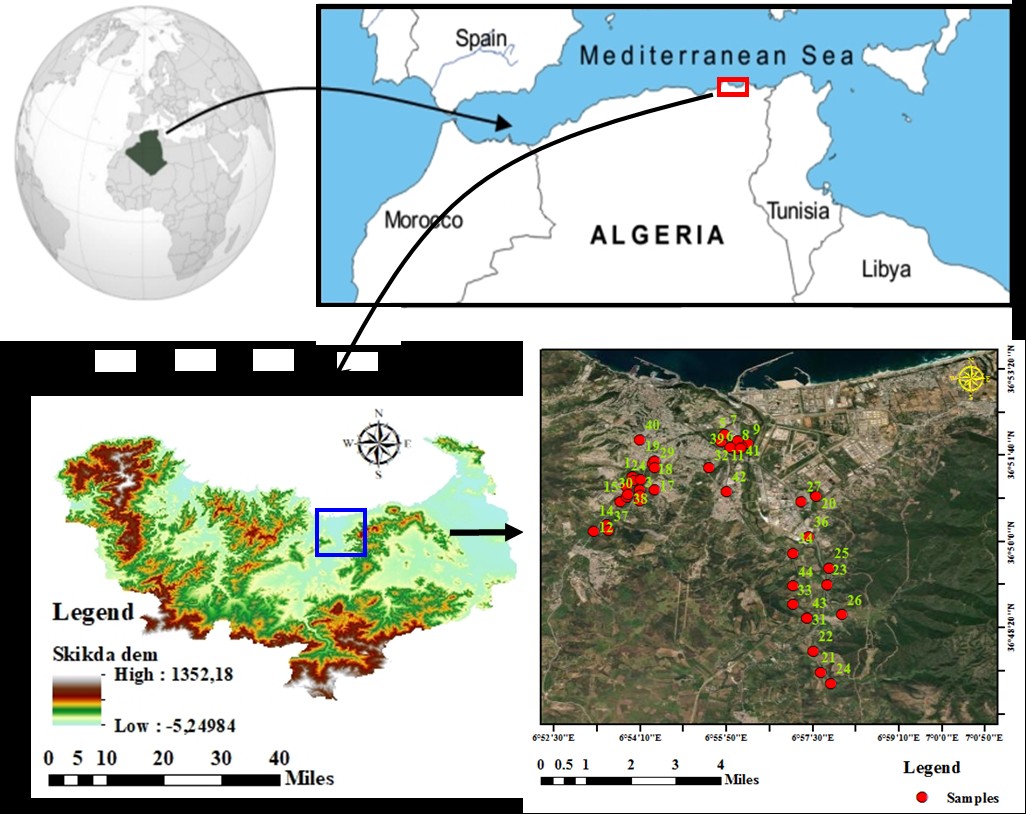

Supplement: Supplementary file 5 — Supplementary Material 5 [file 41598_2025_25461_MOESM5_ESM.jpg]

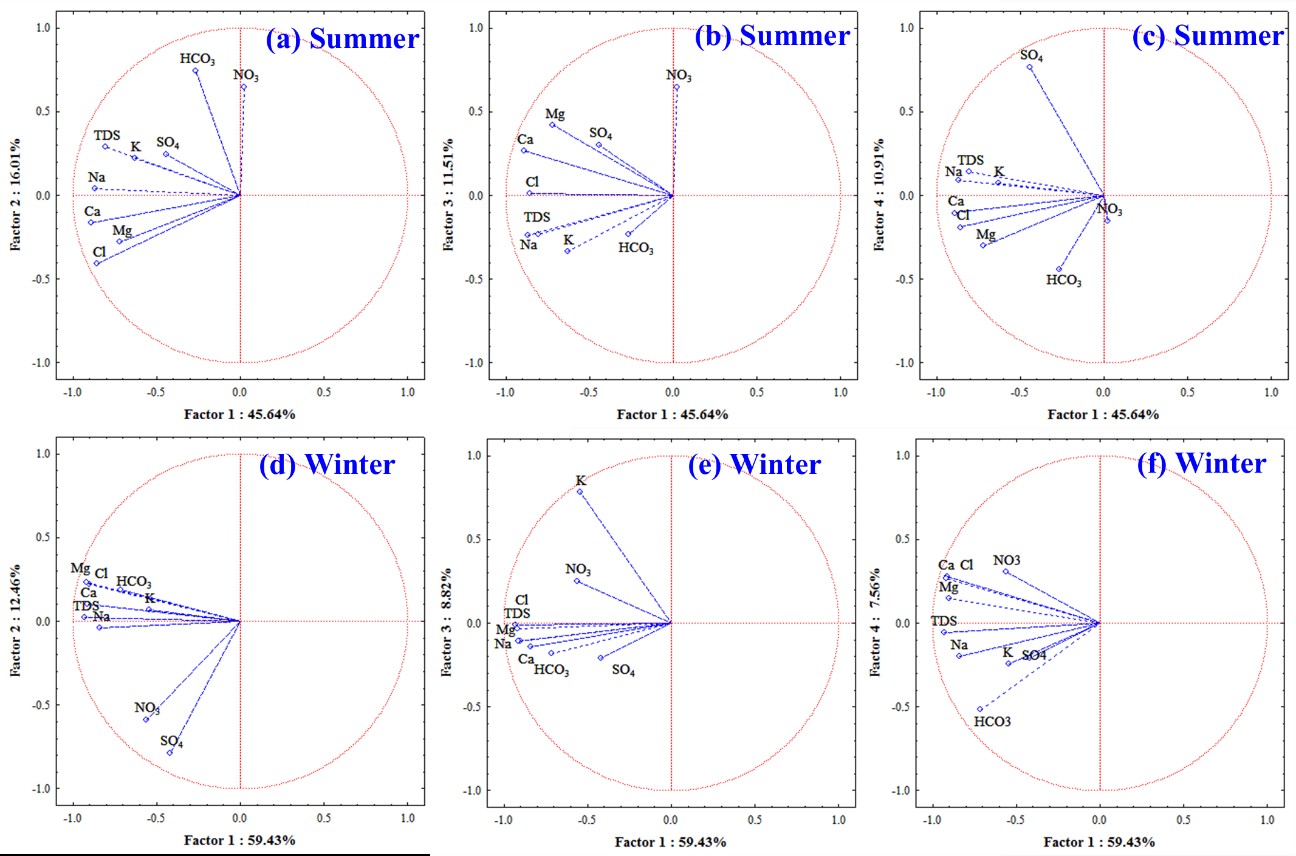

Supplement: Supplementary file 6 — Supplementary Material 6 [file 41598_2025_25461_MOESM6_ESM.jpg]

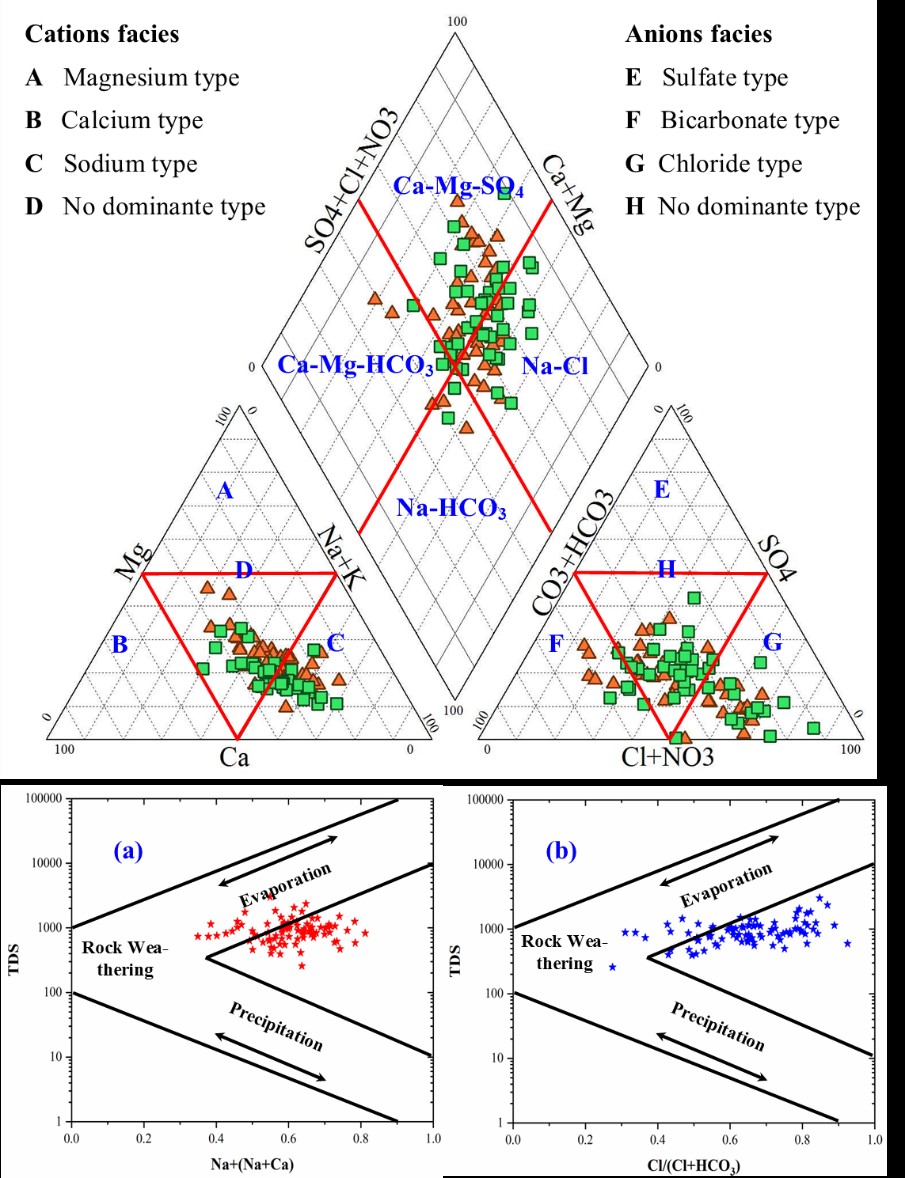

Supplement: Supplementary file 7 — Supplementary Material 7 [file 41598_2025_25461_MOESM7_ESM.jpg]

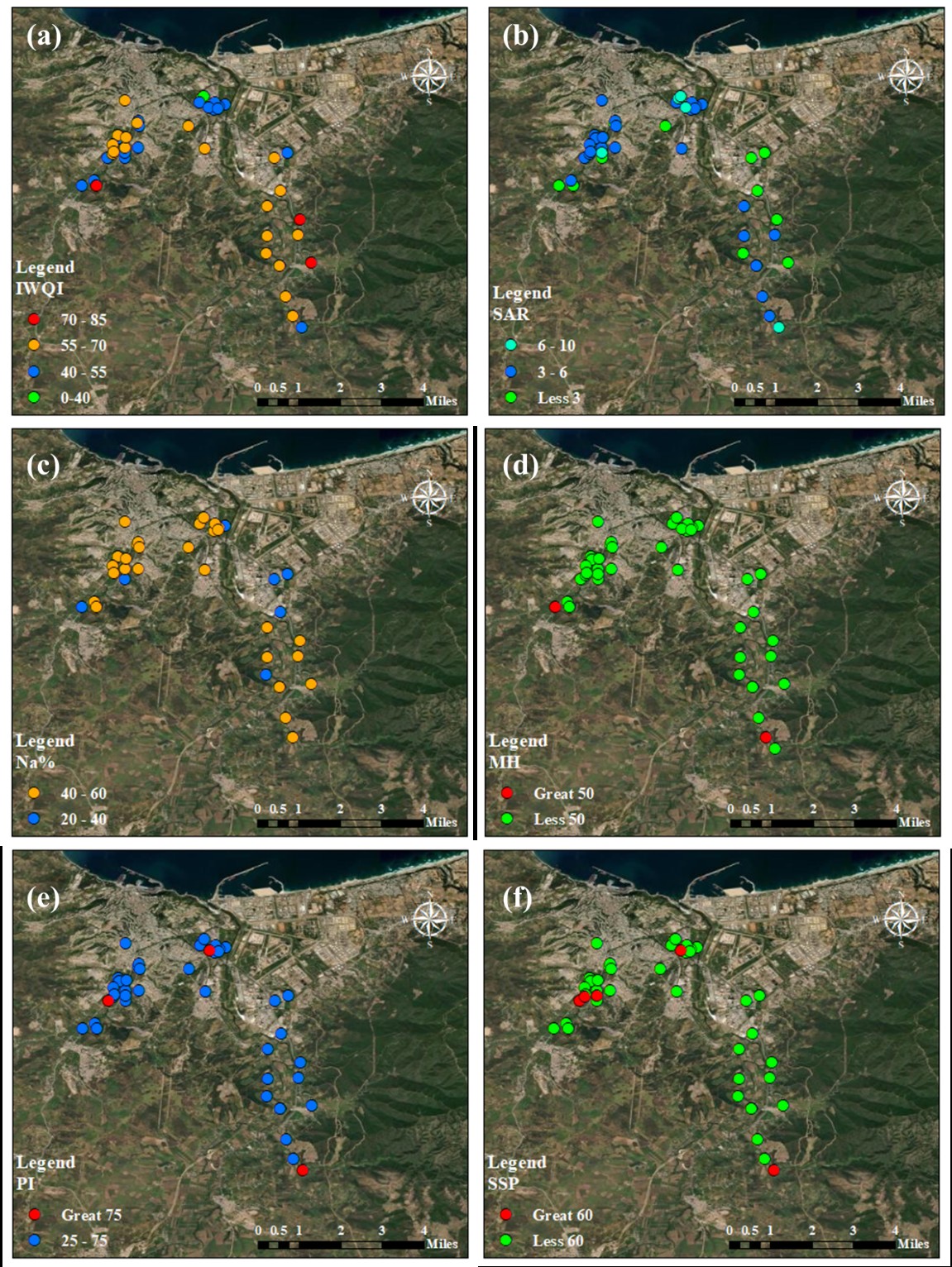

Supplement: Supplementary file 8 — Supplementary Material 8 [file 41598_2025_25461_MOESM8_ESM.jpg]

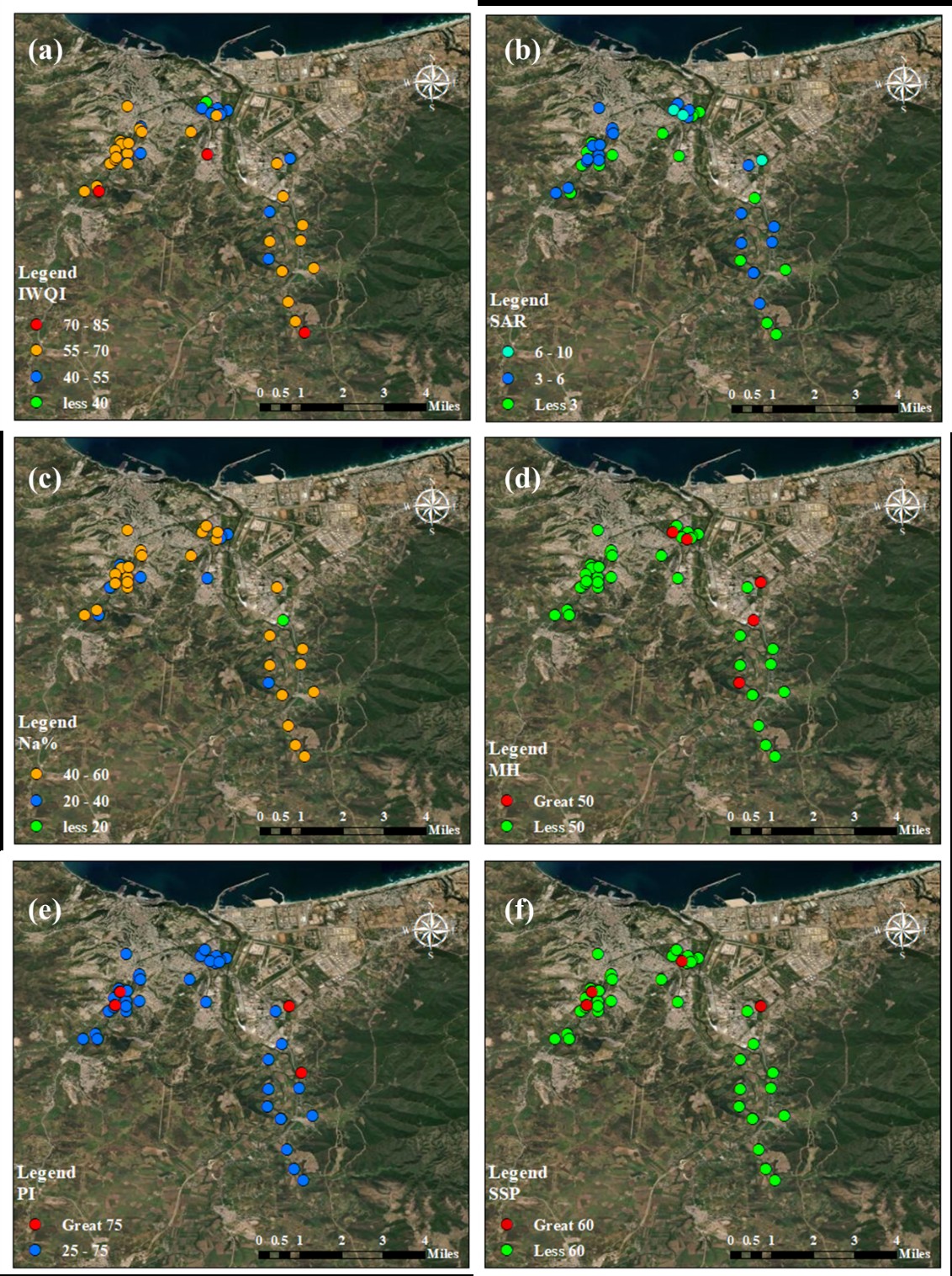

Supplement: Supplementary file 9 — Supplementary Material 9 [file 41598_2025_25461_MOESM9_ESM.jpg]

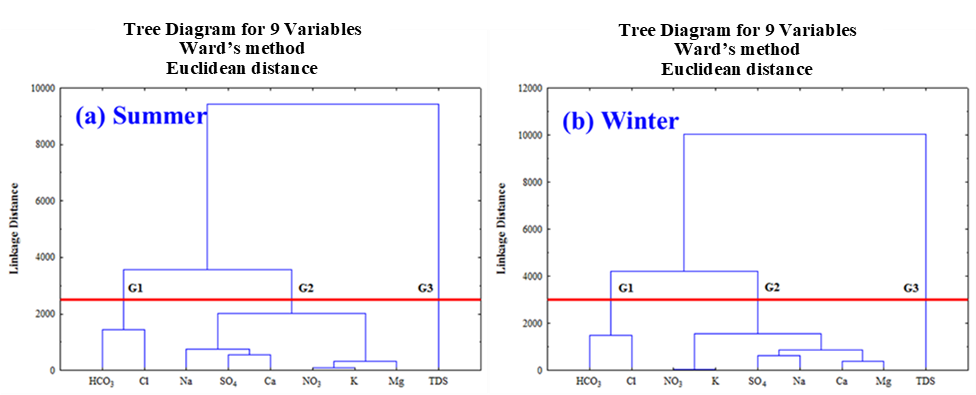

Supplement: Supplementary file 10 — Supplementary Material 10 [file 41598_2025_25461_MOESM10_ESM.tif]

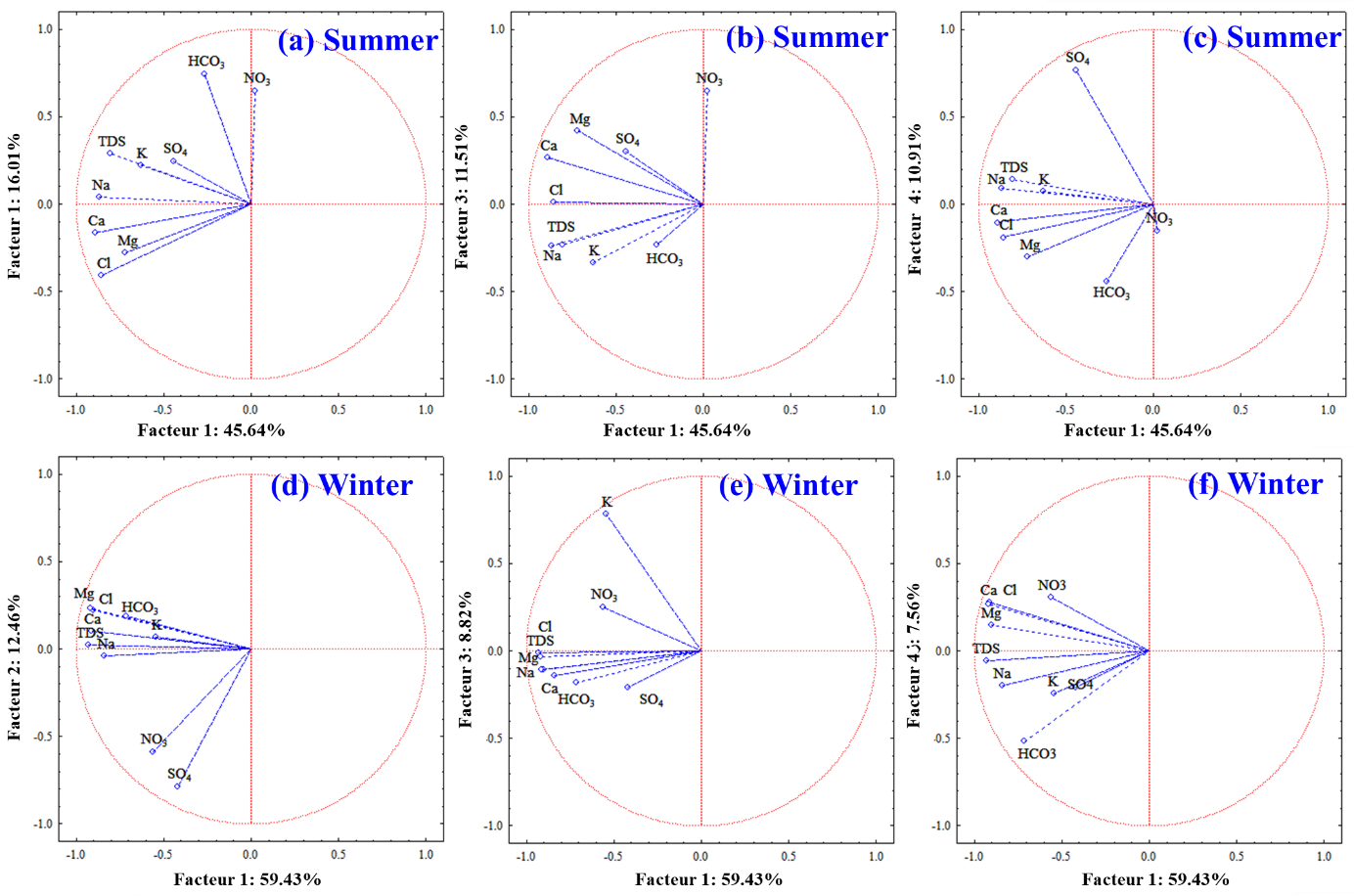

Supplement: Supplementary file 11 — Supplementary Material 11 [file 41598_2025_25461_MOESM11_ESM.tif]

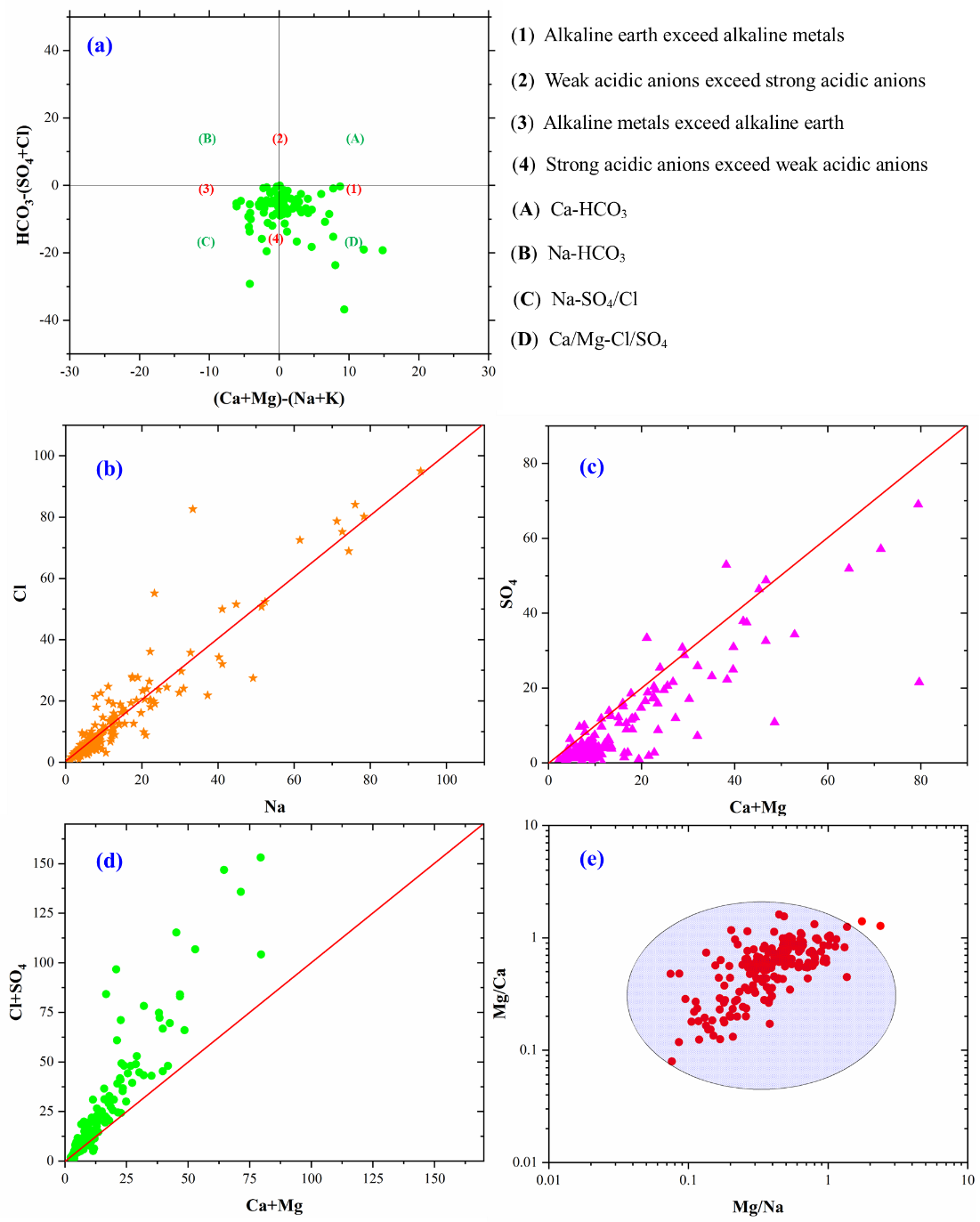

Supplement: Supplementary file 12 — Supplementary Material 12 [file 41598_2025_25461_MOESM12_ESM.tif]

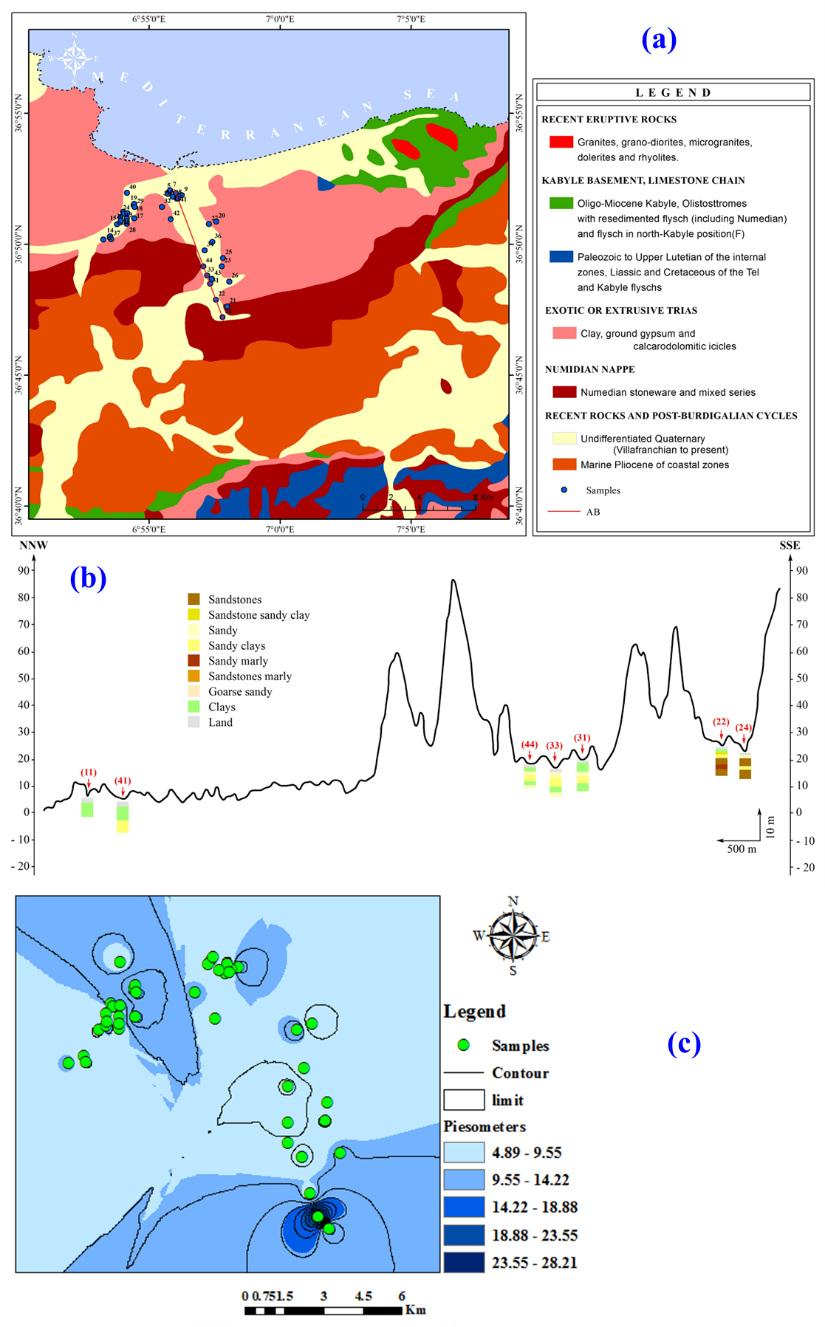

Supplement: Supplementary file 13 — Supplementary Material 13 [file 41598_2025_25461_MOESM13_ESM.tif]

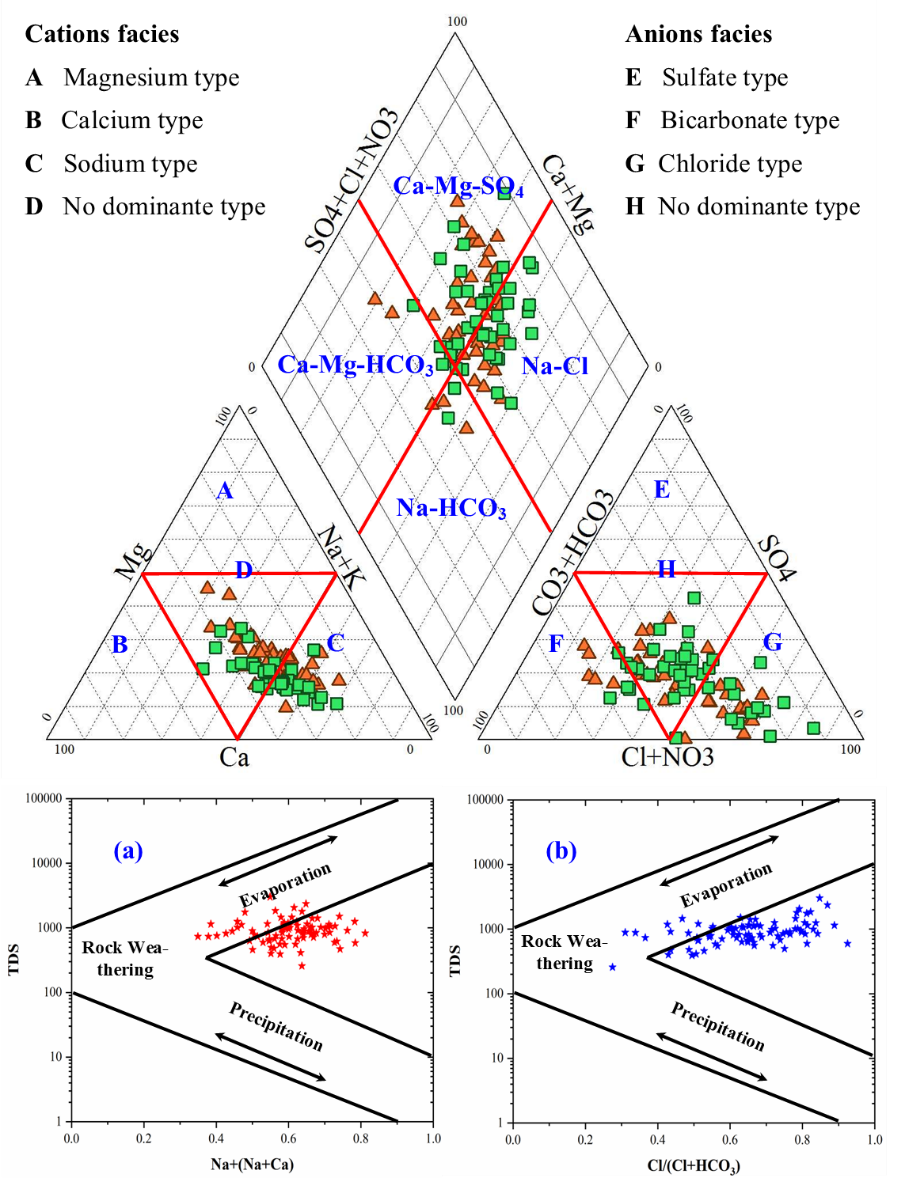

Supplement: Supplementary file 14 — Supplementary Material 14 [file 41598_2025_25461_MOESM14_ESM.tif]

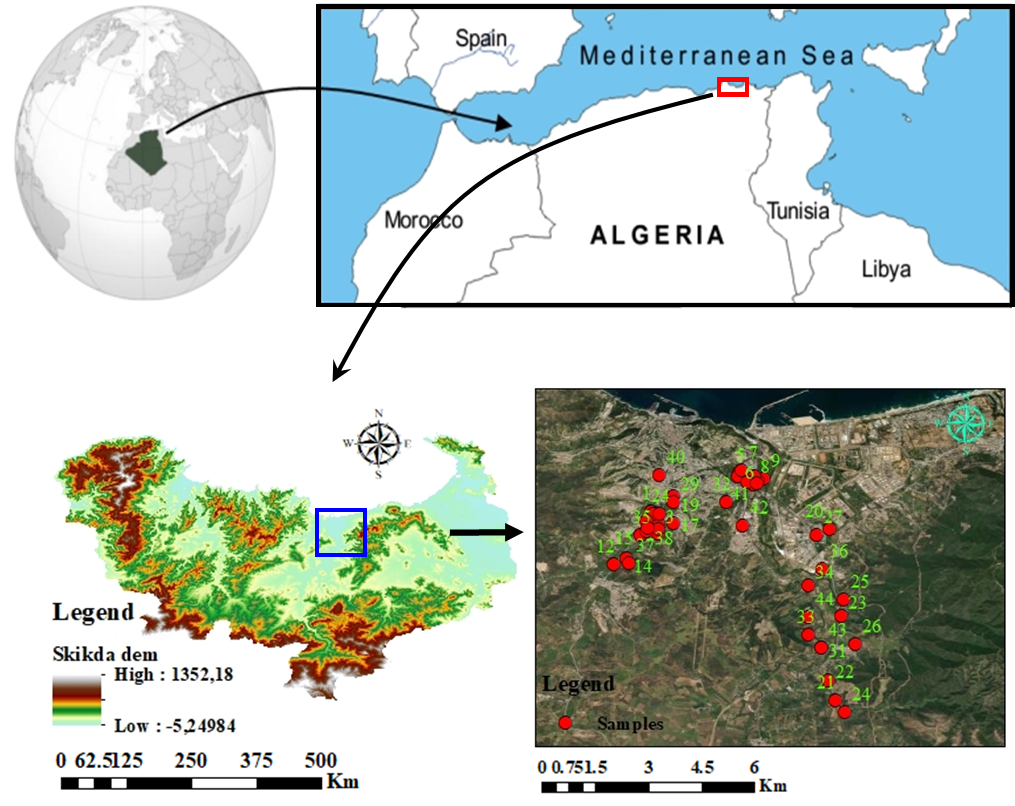

Supplement: Supplementary file 15 — Supplementary Material 15 [file 41598_2025_25461_MOESM15_ESM.tif]

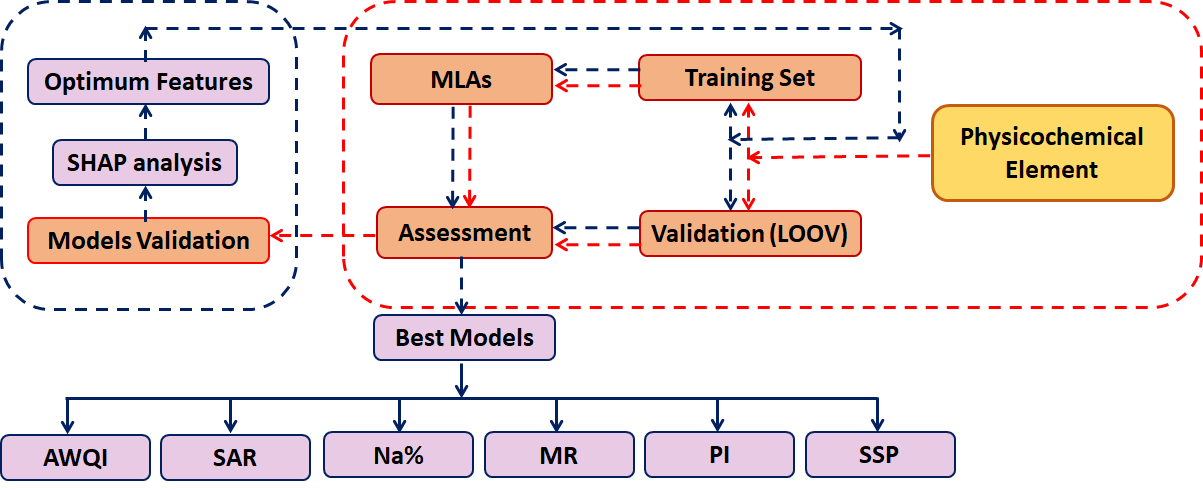

Supplement: Supplementary file 16 — Supplementary Material 16 [file 41598_2025_25461_MOESM16_ESM.tif]

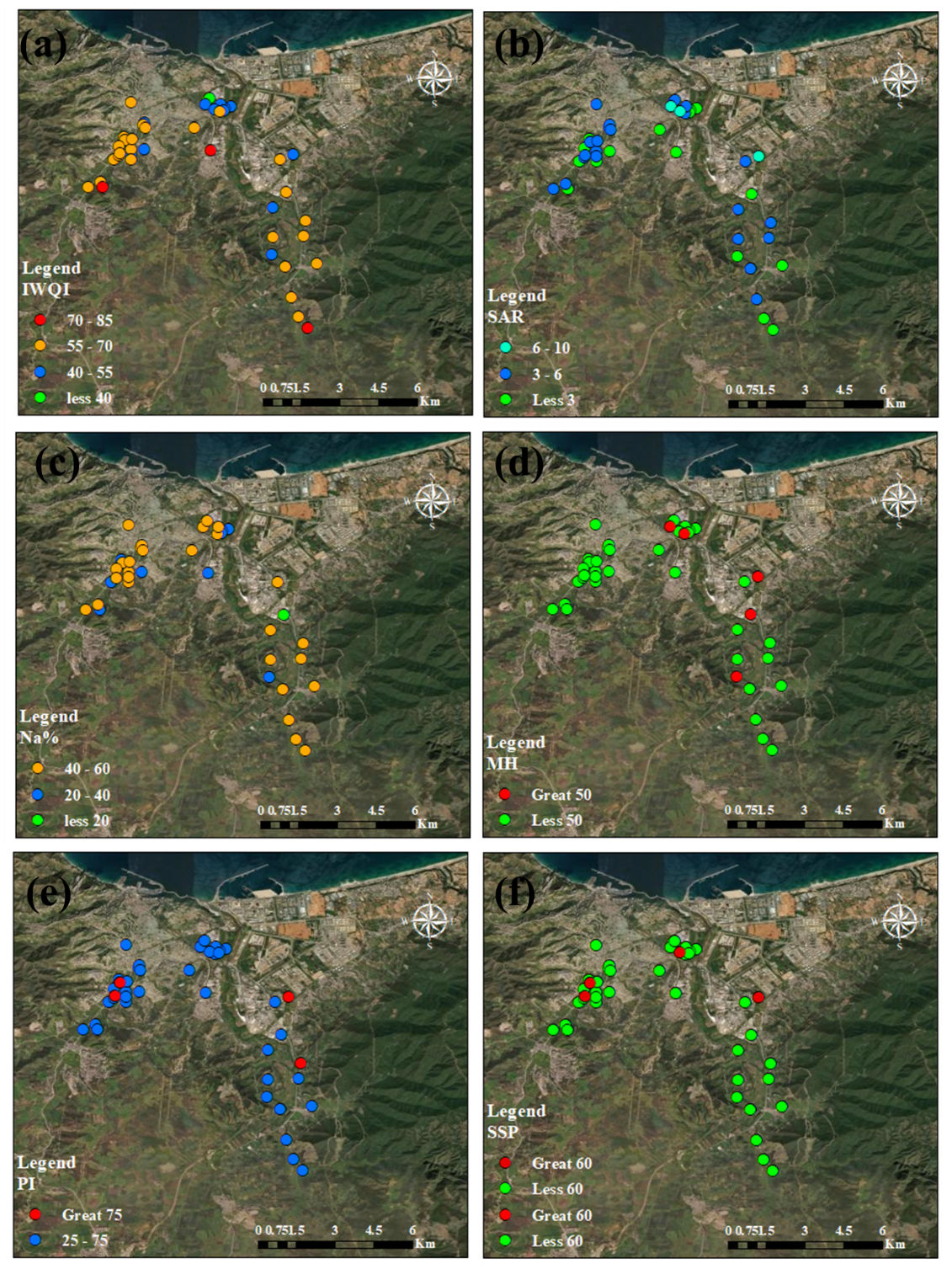

Supplement: Supplementary file 17 — Supplementary Material 17 [file 41598_2025_25461_MOESM17_ESM.tif]

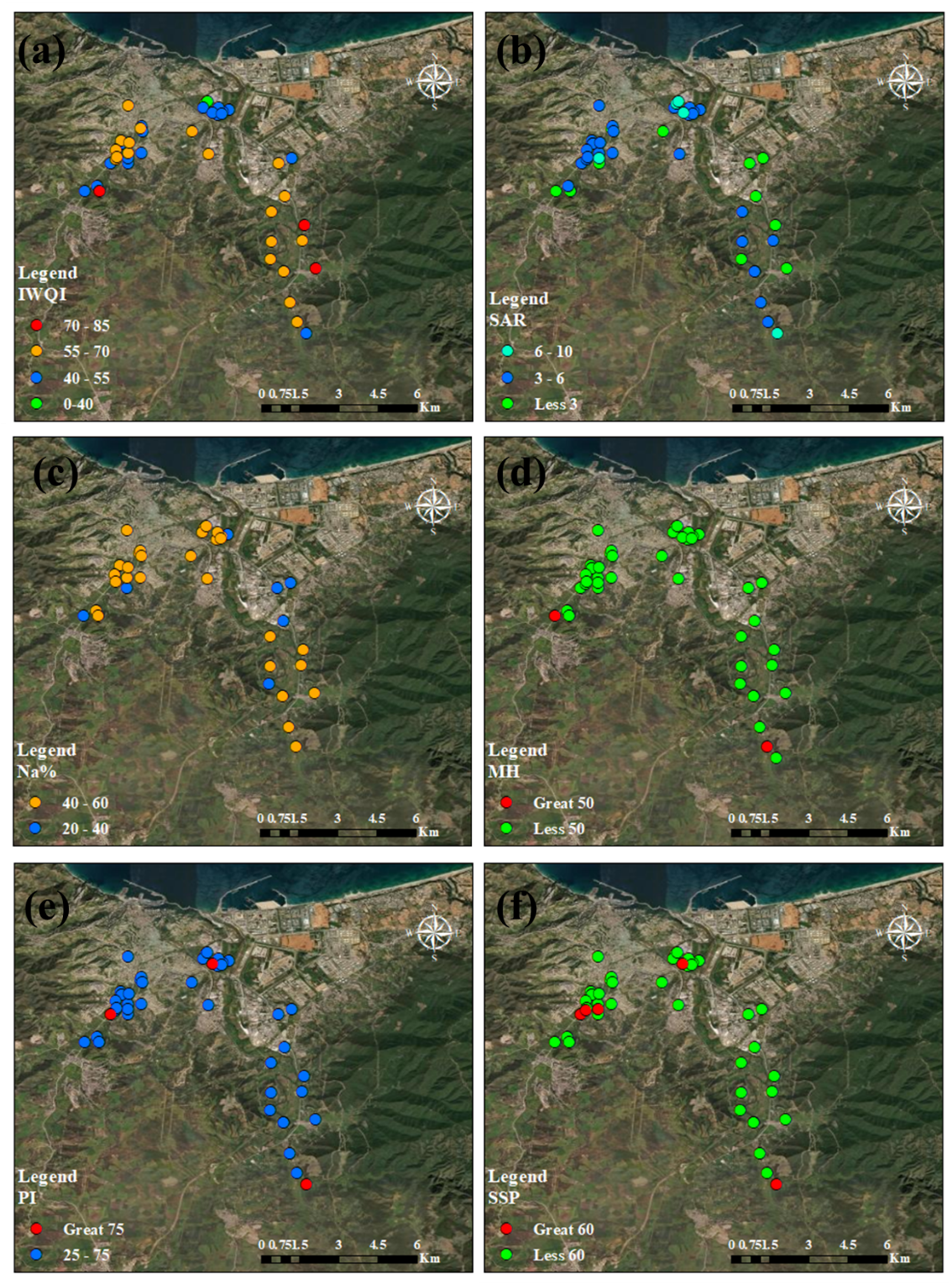

Supplement: Supplementary file 18 — Supplementary Material 18 [file 41598_2025_25461_MOESM18_ESM.tif]
