# Supplementary material for: Integrated GIS-machine learning approach to irrigation water quality assessment in coastal aquifers
Source: Sci Rep. 2026 Jan 28;16:3894. doi: 10.1038/s41598-025-25461-y (PMC12855904; doi:10.1038/s41598-025-25461-y)
Supplement: Supplementary file 19 — Supplementary Material 19 [file 41598_2025_25461_MOESM19_ESM.docx]

**Table S1. Python code for training the machine learning models and selecting the best parameters.**

| # Commented out IPython magic to ensure Python compatibility.  import numpy as np  import pandas as pd  import matplotlib.pyplot as plt  from matplotlib import rcParams  # %matplotlib inline  import seaborn as sns  import shap  shap.initjs()  import sklearn  dataset = pd.read_excel("**dataset.xlsx**")  dataset.head(2)  matrix = daraset.corr()  mask = np.zeros_like(matrix)  mask[np.triu_indices_from(mask)] = True  # Set the figure size and DPI  rcParams['figure.figsize'] = 20, 20  rcParams['figure.dpi'] = 100  # Generate sample data  #data = np.random.rand(10, 10)  #mask = np.triu(np.ones_like(data))  # Create the heatmap  sns.heatmap(  matrix,  center=0,  fmt='.2f',  annot=True,  linewidth=2,  mask=mask,  annot_kws={'size': 12, 'weight': 'bold'}, # Adjust font size and weight here  xticklabels=True,  yticklabels=True,  cbar_kws={'shrink': 0.5, 'aspect': 10}  )  plt.xticks(fontsize=14, weight='bold')  plt.yticks(fontsize=14, weight='bold')  plt.title("Heatmap Flight Data")  plt.show()  from sklearn.model_selection import train_test_split  Y = dataset[Water quality indices]  X = dataset[[various physicochemical element]]  X_train, X_test, Y_train, Y_test = train_test_split(X, Y, test_size = 0.3, random_state = 0)  from sklearn.preprocessing import StandardScaler  scaler = StandardScaler()  X_train = scaler.transform(X_train)  X_test = scaler.transform(X_test)  from sklearn.model_selection import GridSearchCV  from sklearn.metrics import mean_absolute_error, mean_squared_error, r2_score, make_scorer  def nash_sutcliffe_efficiency(y_true, y_pred):  numerator = np.sum((y_true - y_pred) ** 2)  denominator = np.sum((y_true - np.mean(y_true)) ** 2)  return 1 - (numerator / denominator)  def willmott_index(y_true, y_pred):  numerator = np.sum(np.abs(y_pred - y_true))  denominator = np.sum(np.abs(y_pred - np.mean(y_true)) + np.abs(y_true - np.mean(y_true)))  return 1 - (numerator / denominator)  scoring = {  'MAE': make_scorer(mean_absolute_error),  'MSE': make_scorer(mean_squared_error),  'R^2': make_scorer(r2_score),  'RMSE': make_scorer(lambda y, y_pred: np.sqrt(mean_squared_error(y, y_pred))),  'NSE': make_scorer(nash_sutcliffe_efficiency),  'WI': make_scorer(willmott_index)  }  **# Random Forest Model**  from sklearn.ensemble import RandomForestRegressor  Hyper_paramters={'criterion': ['squared_error'], 'max_depth': [1, 2, 3, 4, 5, 6, 7, 8, 9, 10, 11, 12, 13, 14, 15, 16, 17, 18, 19, 20], 'n_estimators' : [1, 2, 3, 4, 5, 6, 7, 8, 9, 10, 11, 12, 13, 14, 15, 16, 17, 18, 19, 20]}  randomforest =GridSearchCV(RandomForestRegressor(random_state = 0),Hyper_paramters,scoring = scoring,n_jobs=-1,  cv=5,return_train_score=False,verbose=0, refit = 'R^2')  randomforest_model = randomforest.fit(X_train,Y_train)  print ("The best paramter combination for randomforest_model: ")  print(randomforest_model.best_params_)  Final_randomforest_model = randomforest_model.best_estimator_  print("The best R squared score for randomforest_model was ")  print(randomforest_model.best_score_)  # Prediction Using the Model  Y_pred = Final_randomforest_model.predict(X_test)  # Calculate metrics for RandomForest  print ("The best paramter combination for randomforest_model: ")  print(randomforest_model.best_params_)  print('Regression Model Performance Metrics:')  mae = mean_absolute_error(Y_test), Y_pred, multioutput = 'uniform_average')  print(f'Mean Absolute Error (MAE): {mae:.4f}')  mse = mean_squared_error(Y_test, Y_pred, multioutput = 'uniform_average')  print('Mean Squared Error: ' + str(mse))  rmse = np.sqrt(mse)  print(f'Root Mean Square Error (RMSE): {rmse:.4f}')  r2 = r2_score(Y_test, Y_pred, multioutput = 'uniform_average')  print(f'Coefficient of Determination (R²): {r2:.4f}')  nse = nash_sutcliffe_efficiency(Y_test, Y_pred)  print(f'Nash-Sutcliffe Efficiency (NSE): {nse:.4f}')  wi = willmott_index(Y_test, Y_pred)  print(f'Willmott Index (WI): {wi:.4f}')  print(list(Y_test))  print(list(Y_pred))  # Assuming you have a list of feature names, replace the example feature_names list below with your actual feature names  feature_names = ['SO4', 'HCO3', 'Cl']  # Get SHAP values  explainer = shap.Explainer(Final_randomforest_model.predict, X_train)  shap_values = explainer(X_train)  # Beeswarm plot  #shap.plots.beeswarm(shap_values)  #shap.summary_plot(shap_values, plot_type='violin')  shap.summary_plot(shap_values, feature_names=feature_names, plot_type='violin')  #shap.plots.beeswarm(shap_values.abs, color="shap_red")  plt.show()  shap.plots.bar(shap_values)  **# AdaBoost Model**  from sklearn.ensemble import AdaBoostRegressor  # Define the Grid search parameters for AdaBoost  Hyper_paramters = {  'n_estimators': [1, 2, 3, 4, 5, 6, 7, 8, 9, 10, 11, 12, 13, 14, 15, 16, 17, 18, 19, 20],  'learning_rate': [0.01, 0.1]  }  # AdaBoost Grid Search  adaboost =GridSearchCV(AdaBoostRegressor(random_state = 0),Hyper_paramters,scoring = scoring,n_jobs=-1,  cv=5,return_train_score=False,verbose=0, refit = 'R^2')  adaboost_model = adaboost.fit(X_train, Y_train)  print ("The best paramter combination for AdaBoost model: ")  print(adaboost_model.best_params_)  Final_adaboost_model = adaboost_model.best_estimator_  print("The best R squared score for AdaBoost model was ")  print(adaboost_model.best_score_)  # Prediction Using the Model  Y_pred = Final_adaboost_model.predict(X_test)  # Calculate metrics for AdaBoost  print ("The best paramter combination for AdaBoost model: ")  print(adaboost_model.best_params_)  print('Regression Model Performance Metrics:')  mae = mean_absolute_error(Y_test, Y_pred, multioutput = 'uniform_average')  print(f'Mean Absolute Error (MAE): {mae:.4f}')  mse = mean_squared_error(Y_test, Y_pred, multioutput = 'uniform_average')  print('Mean Squared Error: ' + str(mse))  rmse = np.sqrt(mse)  print(f'Root Mean Square Error (RMSE): {rmse:.4f}')  r2 = r2_score(Y_test, Y_pred, multioutput = 'uniform_average')  print(f'Coefficient of Determination (R²): {r2:.4f}')  nse = nash_sutcliffe_efficiency(Y_test, Y_pred)  print(f'Nash-Sutcliffe Efficiency (NSE): {nse:.4f}')  wi = willmott_index(Y_test, Y_pred)  print(f'Willmott Index (WI): {wi:.4f}')  print(list(Y_test))  print(list(Y_pred))  # Assuming you have a list of feature names, replace the example feature_names list below with your actual feature names  feature_names = ['SO4', 'HCO3', 'Cl']  # Get SHAP values  explainer = shap.Explainer(Final_adaboost_model.predict, X_train)  shap_values = explainer(X_train)  # Beeswarm plot  #shap.plots.beeswarm(shap_values)  #shap.summary_plot(shap_values, plot_type='violin')  shap.summary_plot(shap_values, feature_names=feature_names, plot_type='violin')  #shap.plots.beeswarm(shap_values.abs, color="shap_red")  plt.show()  shap.plots.bar(shap_values)  **# XGBRegressor Model**  #%pip install xgboost  from xgboost import XGBRegressor  # Define the Grid search parameters for XGBoost  Hyper_paramters = {  'n_estimators': [1, 2, 3, 4, 5, 6, 7, 8, 9, 10, 11, 12, 13, 14, 15, 16, 17, 18, 19, 20],  'learning_rate': [0.01,0.02,0.05],  'max_depth': [1, 2, 3, 4, 5, 6, 7, 8, 9, 10, 11, 12, 13, 14, 15, 16, 17, 18, 19, 20]  }  # XGBoost Grid Search  xgboost =GridSearchCV(XGBRegressor(random_state = 0),Hyper_paramters,scoring = scoring,n_jobs=-1,  cv=5,return_train_score=False,verbose=0, refit = 'R^2')  xgboost_model = xgboost.fit(X_train, Y)  print ("The best paramter combination for XGBoost model: ")  print(xgboost_model.best_params_)  Final_xgboost_model = xgboost_model.best_estimator_  print("The best R squared score for XGBoost model was ")  print(xgboost_model.best_score_)  # Prediction Using the Model  Y_pred = Final_xgboost_model.predict(X_test)  # Calculate metrics for XGBoost  print ("The best paramter combination for XGBoost model: ")  print(xgboost_model.best_params_)  print('Regression Model Performance Metrics:')  mae = mean_absolute_error(Y_test, Y_pred, multioutput = 'uniform_average')  print(f'Mean Absolute Error (MAE): {mae:.4f}')  mse = mean_squared_error(Y_test, Y_pred, multioutput = 'uniform_average')  print('Mean Squared Error: ' + str(mse))  rmse = np.sqrt(mse)  print(f'Root Mean Square Error (RMSE): {rmse:.4f}')  r2 = r2_score(Y_test, Y_pred, multioutput = 'uniform_average')  print(f'Coefficient of Determination (R²): {r2:.4f}')  nse = nash_sutcliffe_efficiency(Y_test, Y_pred)  print(f'Nash-Sutcliffe Efficiency (NSE): {nse:.4f}')  wi = willmott_index(Y_test, Y_pred)  print(f'Willmott Index (WI): {wi:.4f}')  print(list(Y_test))  print(list(Y_pred))  # Assuming you have a list of feature names, replace the example feature_names list below with your actual feature names  feature_names = ['SO4', 'HCO3', 'Cl']  # Get SHAP values  explainer = shap.Explainer(Final_xgboost_model.predict, X_train)  shap_values = explainer(X_train)  # Beeswarm plot  #shap.plots.beeswarm(shap_values)  #shap.summary_plot(shap_values, plot_type='violin')  shap.summary_plot(shap_values, feature_names=feature_names, plot_type='violin')  #shap.plots.beeswarm(shap_values.abs, color="shap_red")  plt.show()  shap.plots.bar(shap_values) |
| --- |
