# Supplementary material for: Integrated GIS-machine learning approach to irrigation water quality assessment in coastal aquifers
Source: Sci Rep. 2026 Jan 28;16:3894. doi: 10.1038/s41598-025-25461-y (PMC12855904; doi:10.1038/s41598-025-25461-y)
Supplement: Supplementary file 20 — Supplementary Material 20 [file 41598_2025_25461_MOESM20_ESM.docx]

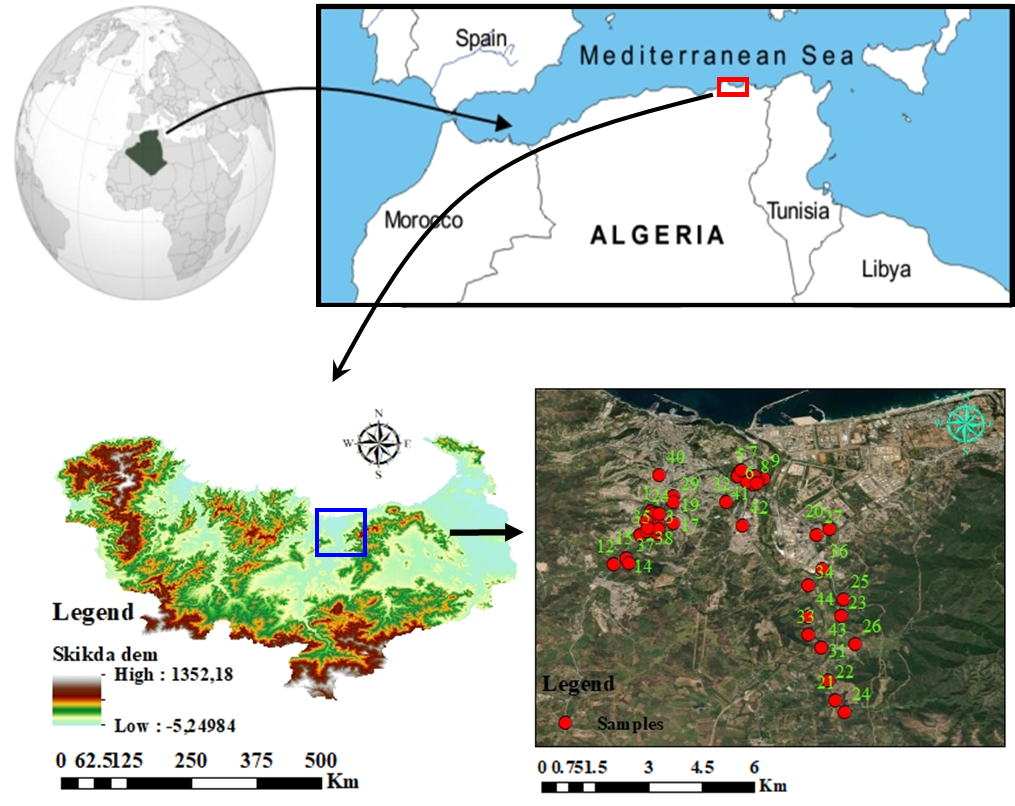


**Figure 1.** The Location map of the study area explains the geographical location, elevation and groundwater points. Map created using ArcGIS Pro 2.8.8 Software (Esri; <https://www.esri.com/arcgis/about-arcgis>).


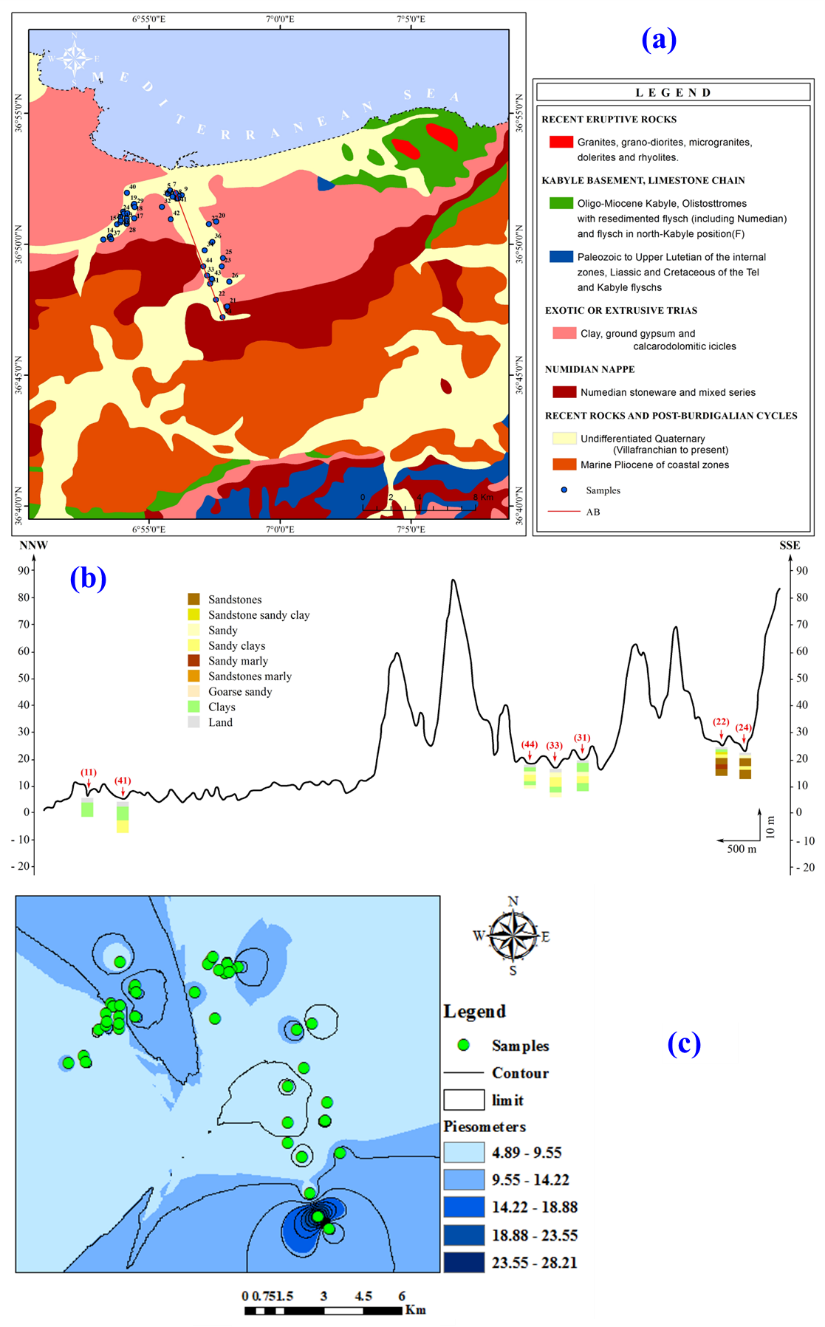


**Figure 2.** General map including: (a) geological categories, (b) cross section in the study region, and (c) piezometric map. Map 2 (c) created using ArcGIS Pro 2.8.8 Software (Esri; <https://www.esri.com/arcgis/about-arcgis>).

**Figure 3.** Flowchart for predicting WQIs, such as IWQI, SAR, Na%, MR, PI, and SSP using different MLAs based on various physicochemical element.


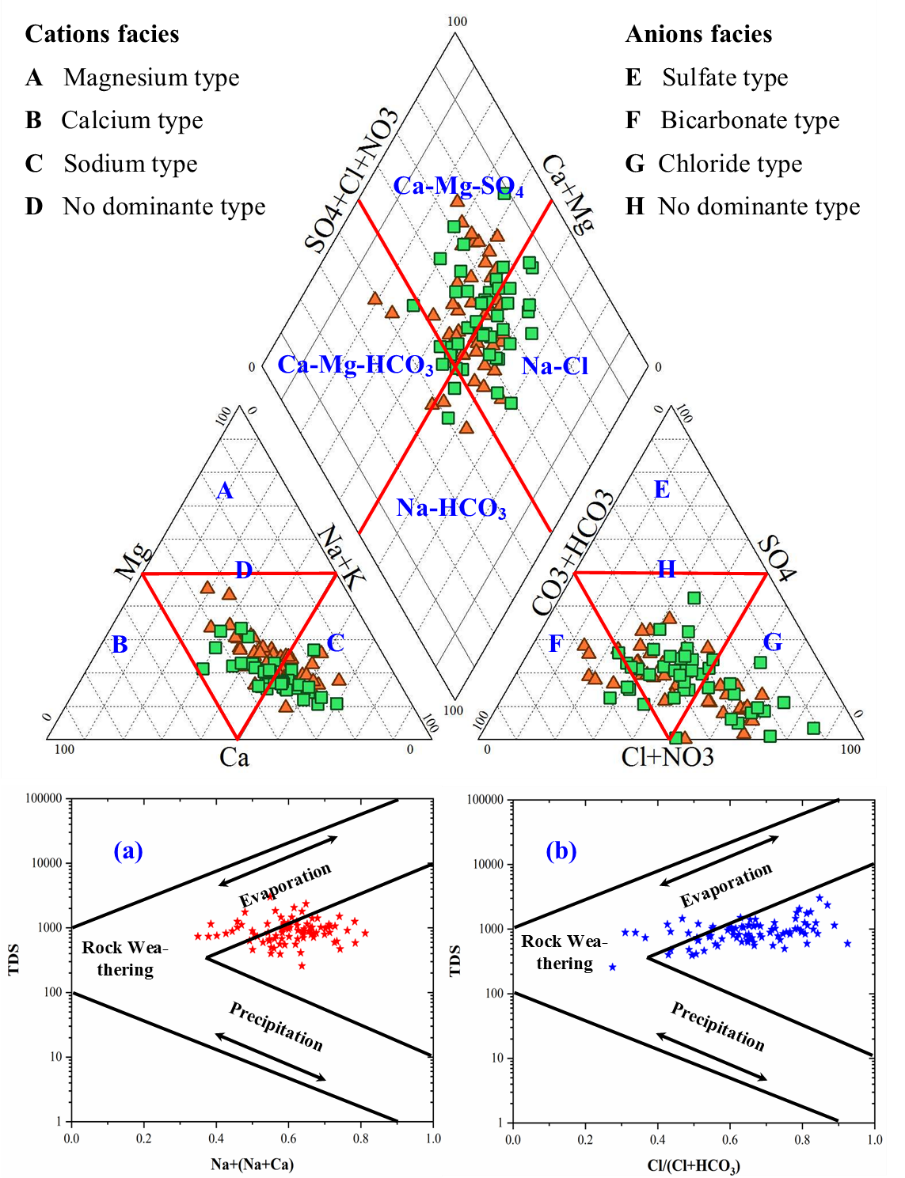


**Figure 4.** Groundwater evolution in Morang based on (a) Piper diagram and (b) Gibbs plots.


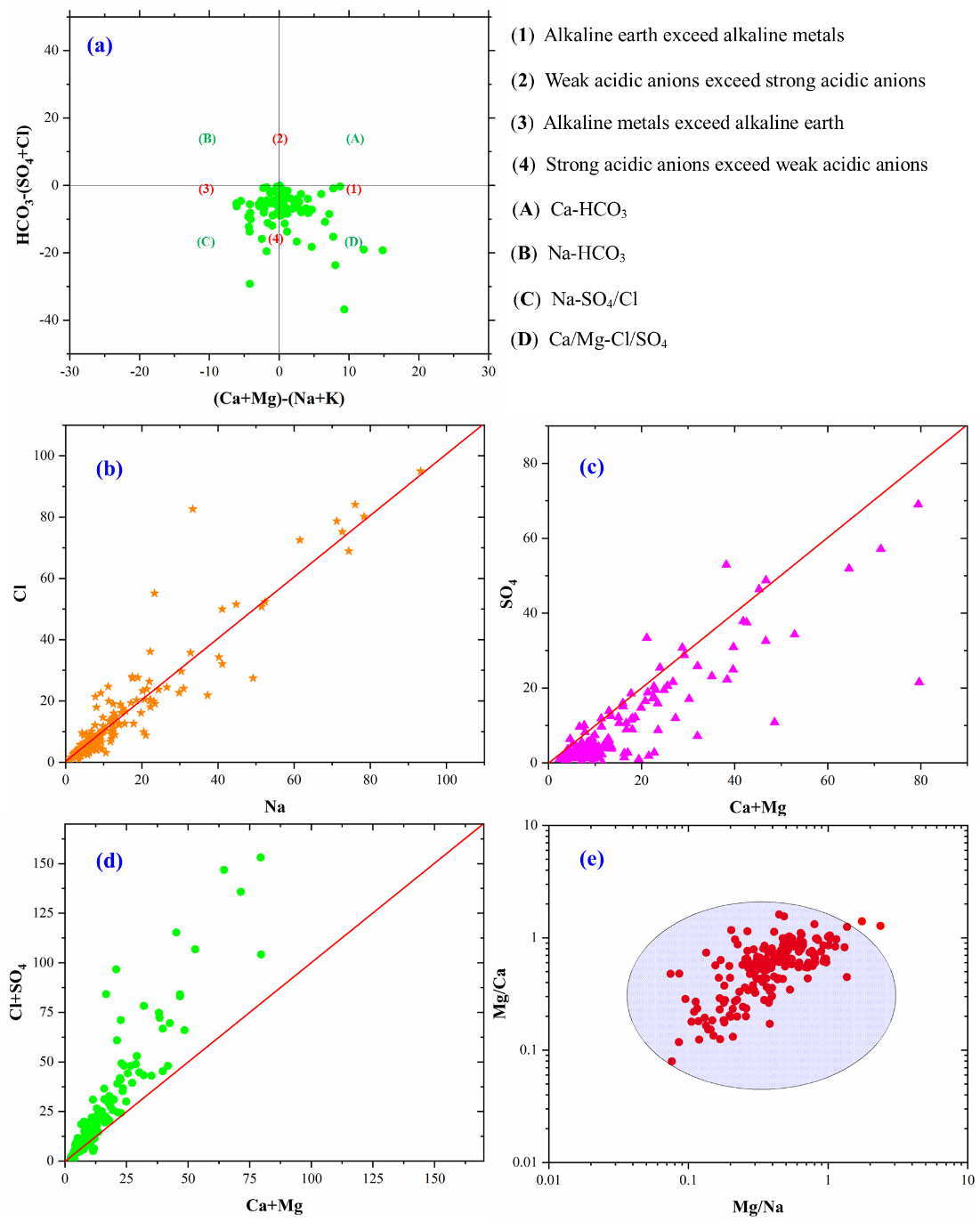


**Figure 5.** Mechanism controlling groundwater chemistry in Morang aquifer system based on ionic ratios: (a) (Ca+Mg) – (Na+K) vs. (HCO_3_) – (SO_4_+Cl), (b) Na vs. Cl, (c) (Ca+Mg) vs. SO_4_ (d) (Ca+Mg) vs. (Cl+SO_4_), and (e) Mg/Na vs. Mg/Ca.


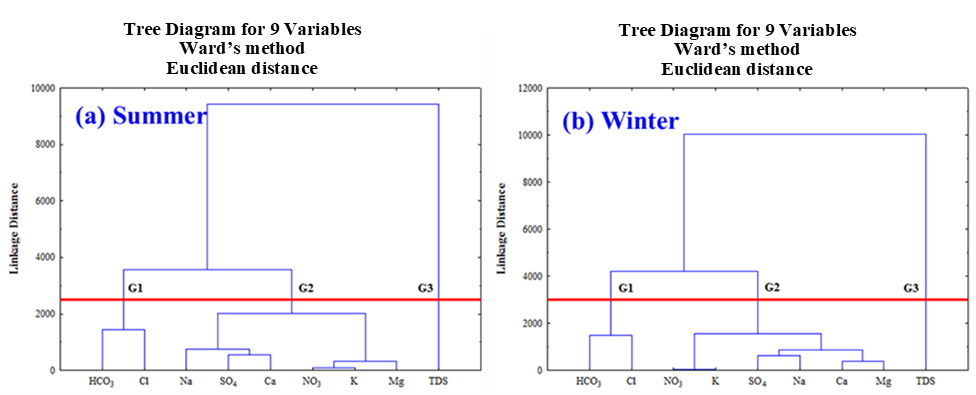


**Figure 6.** The number of clusters or groups extracted from dendrogram branches.


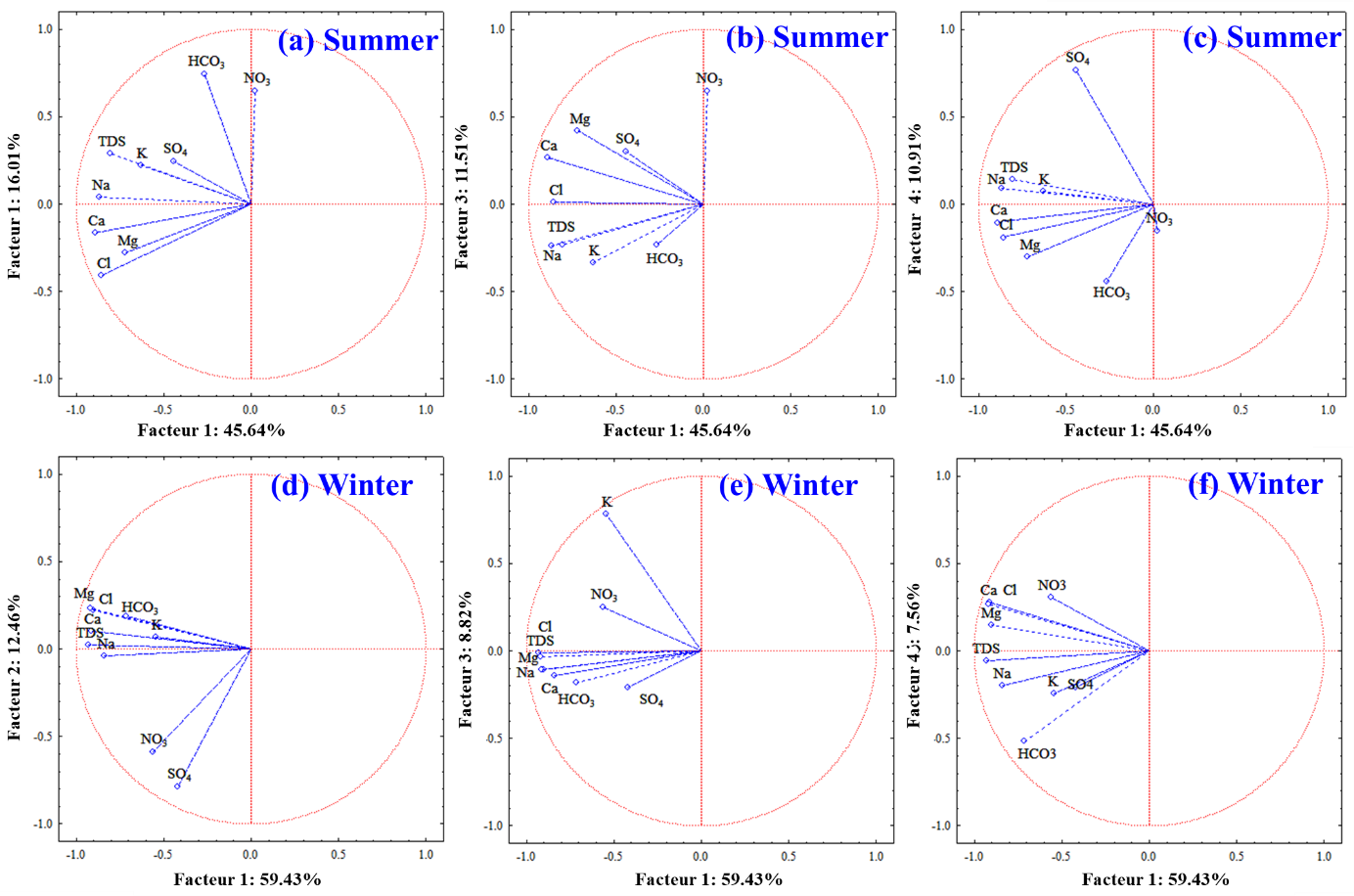


**Figure 7.** Visualization of the three factors or components extracted according to eigenvalue


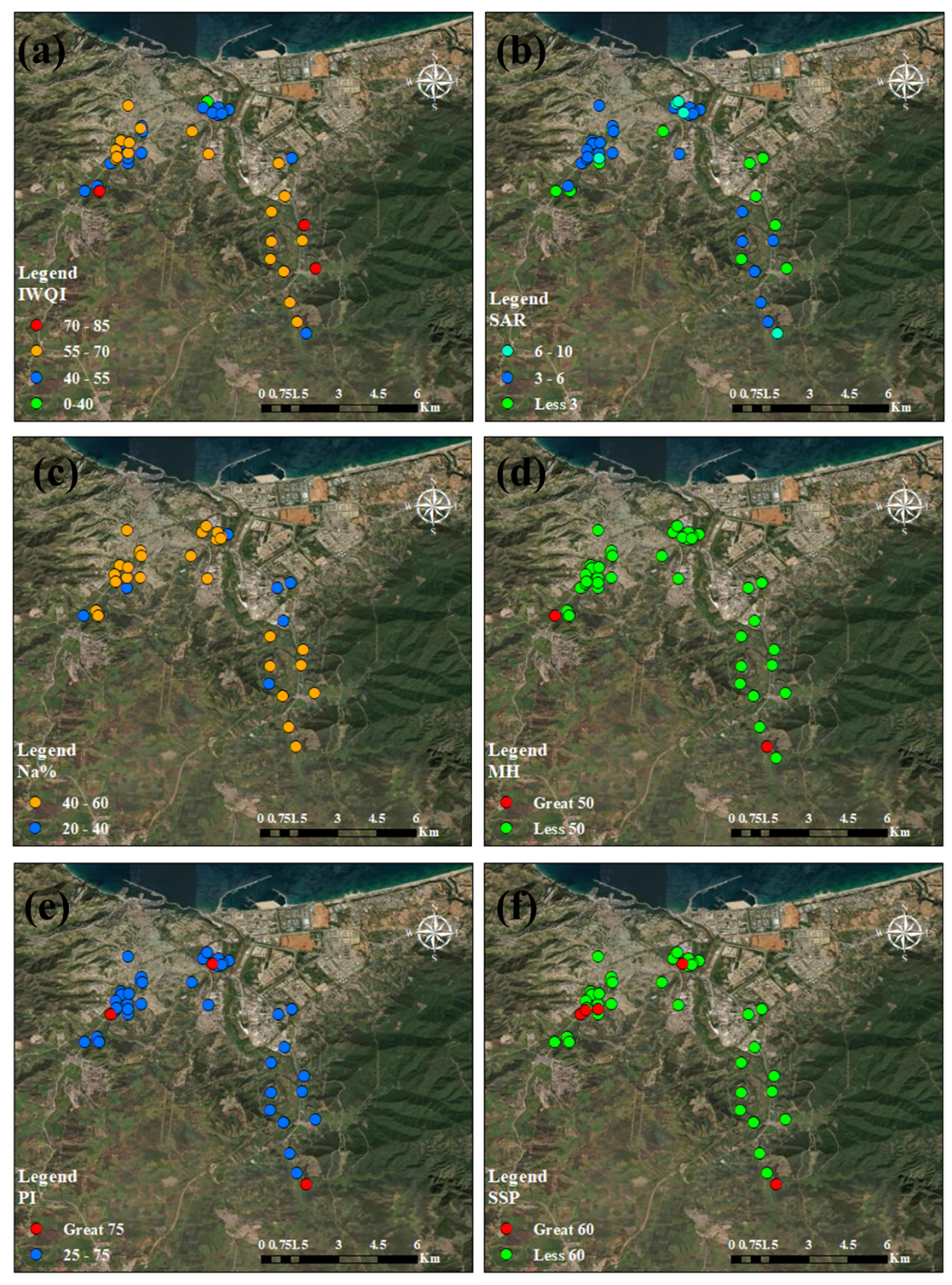


**Figure 8.** Application of GIS interpolation to detect the spatial classes of different indices and contaminated locations for summer period. Map created using ArcGIS Pro 2.8.8 Software (Esri; <https://www.esri.com/arcgis/about-arcgis>).


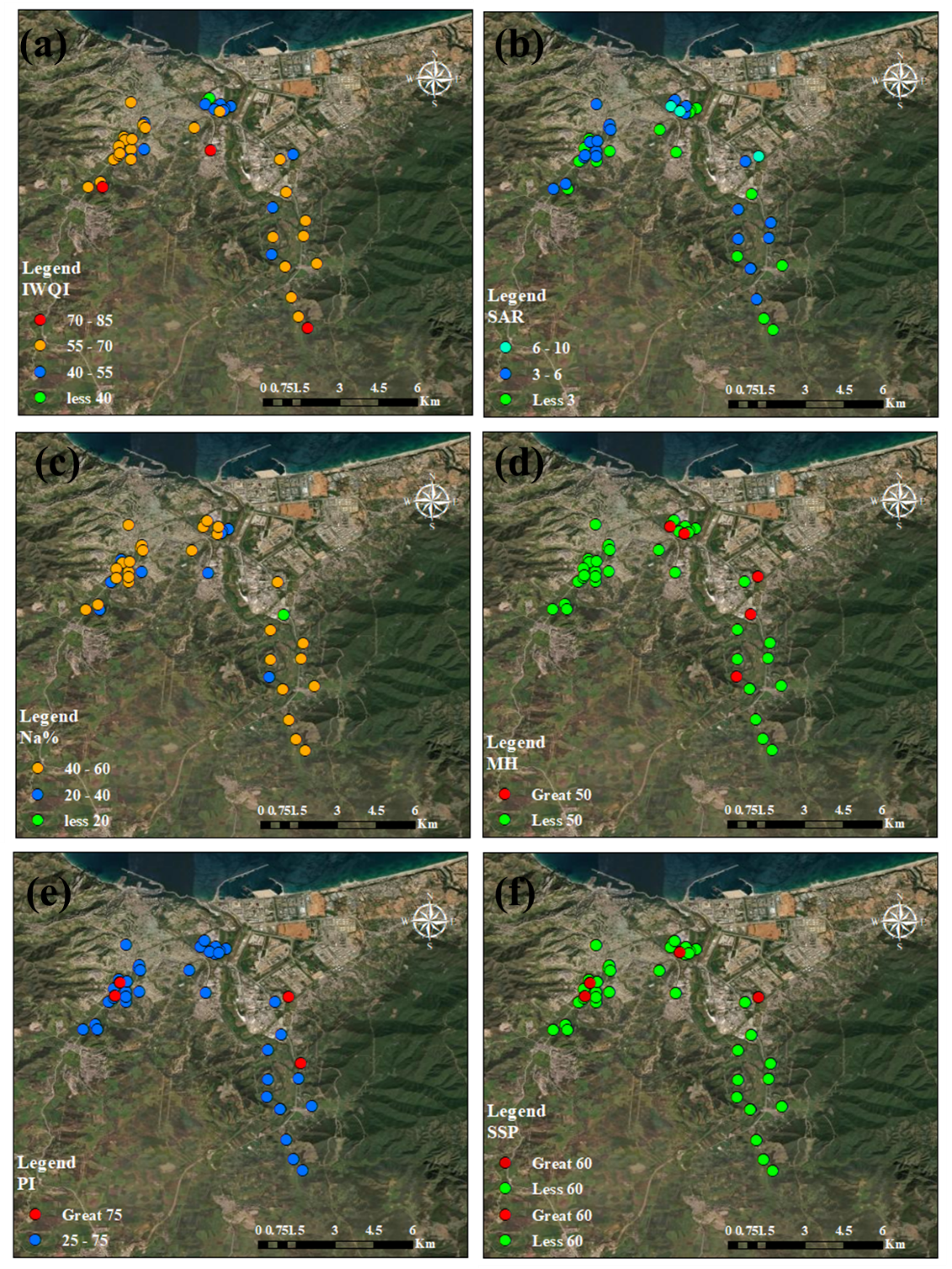


**Figure 9.** Application of GIS interpolation to detect the spatial classes of different indices and contaminated locations for winter period. Map created using ArcGIS Pro 2.8.8 Software (Esri; <https://www.esri.com/arcgis/about-arcgis>).
